# Synthesis, molecular docking study and biological evaluation of new pyrrole scaffolds as potential antitubercular agents for dual targeting of enoyl ACP reductase and dihydrofolate reductase

**DOI:** 10.1371/journal.pone.0303173

**Published:** 2024-05-13

**Authors:** Mater H. Mahnashi, Sravanthi Avunoori, Sanjay Gopi, Ibrahim Ahmed Shaikh, Ahmed Saif, Farkad Bantun, Hani Saleh Faidah, Abdulrahman Ali Alhadi, Jaber Hassan Alshehri, Abdullah Ali Alharbi, Prem Kumar S. R., Shrinivas D. Joshi

**Affiliations:** 1 Department of Pharmaceutical Chemistry, College of Pharmacy, Najran University, Najran, Saudi Arabia; 2 Department of Pharmaceutical Chemistry, Novel Drug Design and Discovery Laboratory, SET’s College of Pharmacy, Sangolli Rayanna Nagar, Dharwad, Karnataka, India; 3 Department of Pharmacology, College of Pharmacy, Najran University, Najran, Saudi Arabia; 4 Department of Clinical Laboratory Sciences, College of Applied Medical Sciences, King Khalid University, Abha, Saudi Arabia; 5 Faculty of Medicine, Department of Microbiology, Umm Al-Qura University, Makkah, Saudi Arabia; 6 Faculty of Medicine, Department of Microbiology, Umm Al-Qura University, Holy Makkah, Kingdom of Saudi Arabia; 7 Microbiology Section Pathology and Laboratory Medicine, Armed Forces Hospital Southern Region, Khamis Mushait, Kingdom of Saudi Arabia; 8 Department of Pharmaceutical Chemistry, MVM College of Pharmacy, Yelahanka, Bengaluru, Karnataka, India; Vignan Pharmacy College, INDIA

## Abstract

In this study, new series of N’-(2-(substitutedphenoxy)acetyl)-4-(1*H*-pyrrol-1-yl)benzohydrazides (**3a-j**) 4-(2,5-dimethyl-1*H*-pyrrol-1-yl)-N’-(2-(substitutedphenoxy)acetyl)benzohydrazides (**5a-j**) were synthesized, characterized and assessed as inhibitors of enoyl ACP reductase and DHFR. Most of the compounds exhibited dual inhibition against the enzymes enoyl ACP reductase and DHFR. Several synthesized substances also demonstrated significant antibacterial and antitubercular properties. A molecular docking analysis was conducted in order to determine the potential mechanism of action of the synthesized compounds. The results indicated that there were binding interactions seen with the active sites of dihydrofolate reductase and enoyl ACP reductase. Additionally, important structural details were identified that play a critical role in sustaining the dual inhibitory activity. These findings were useful for the development of future dual inhibitors. Therefore, this study provided strong evidence that several synthesized molecules could exert their antitubercular properties at the cellular level through multi-target inhibition. By shedding light on the mechanisms through which these compounds exert their inhibitory effects, this research opens up promising avenues for the future development of dual inhibitors with enhanced antibacterial and antitubercular properties. The study’s findings underscore the importance of multi-target approaches in drug design, providing a strong foundation for the design and optimization of novel compounds that can effectively target bacterial infections at the cellular level.

## 1. Introduction

Since it has been around for millennia and continues to be extremely contagious, tuberculosis (TB) is a serious threat to human health [1]. According to estimates, the TB bacteria infect almost a quarter of the world’s population. 1.5 million People die from TB each year, which affects 10 million people worldwide. It ranks as one of the most lethal infectious diseases in the world [2]. The bacterium that causes TB, the *Mycobacterium tuberculosis* (Mtb) has a very impermeable cell wall and grows slowly in acid. Mycobacterium tuberculosis (Mtb) is classified as an opportunistic pathogen, capable of entering a dormant state among macrophages for extended periods of time. Subsequently, it can reactivate in persons with weakened immune systems, particularly those co-infected with the human immunodeficiency virus (HIV). The emergence and proliferation of extensively drug-resistant (XDR) and multi-drug-resistant (MDR) tuberculosis (TB) strains exacerbate the existing problem [3,4]. Isoniazid and rifampicin, two widely used medicines, are still ineffective against MDR-TB germs [5–7]. India, China, and the Russian Federation were responsible for the largest proportions of the world load, with India accounting for 27%, China accounting for 14%, and the Russian Federation accounting for 9% [8–11]. Therefore, there is an urgent medical need for the development of novel chemotypes that are safer, more effective, and work through a variety of pathways.

**Fig 1:**
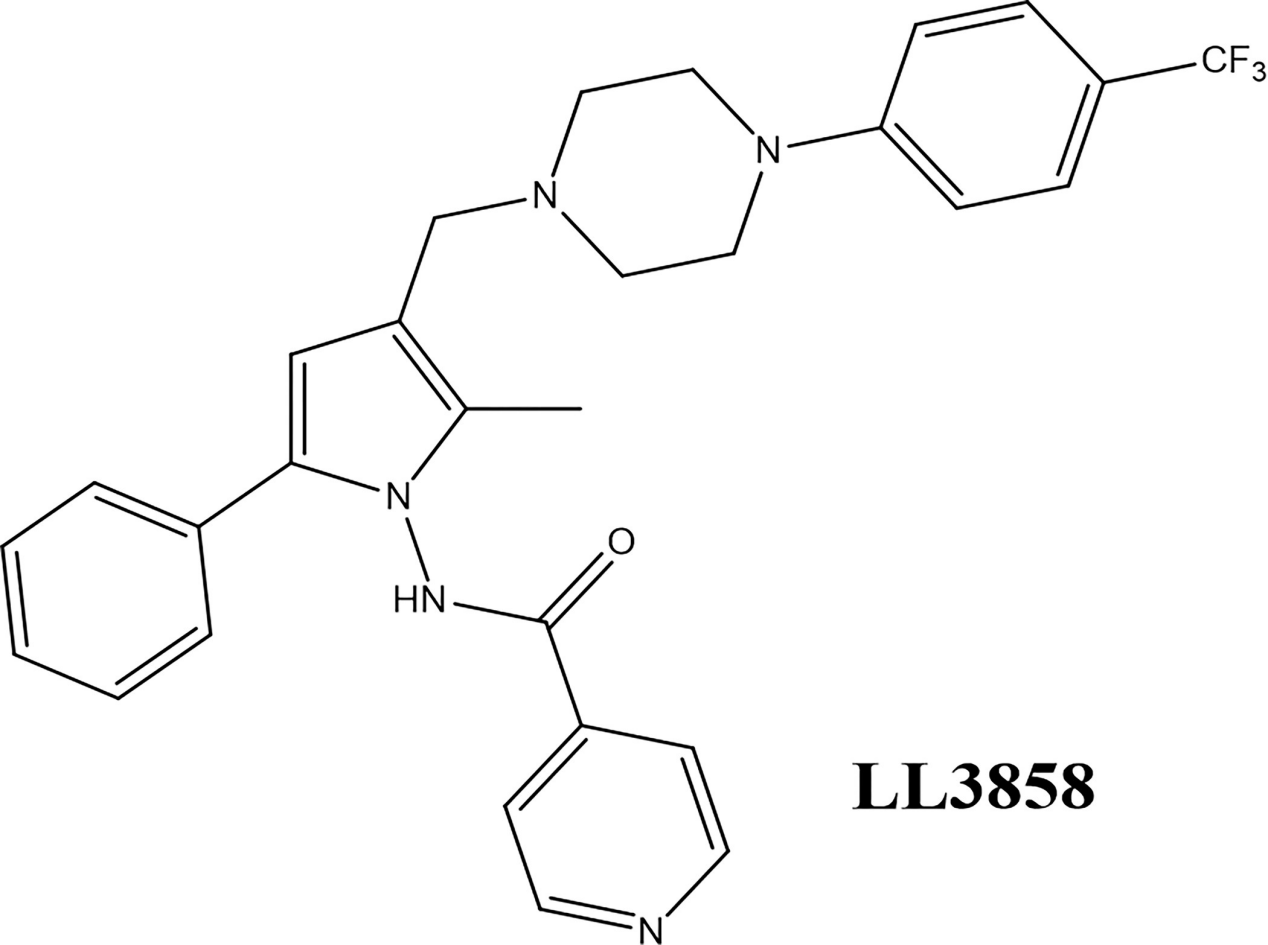
The potent antimycobacterial pyrrole analogue-LL3858.

Numerous pharmaceutical compounds containing nitrogen heterocycles are presently undergoing clinical trials with the aim of treating TB [12]. Pyrroles are notable within this category due to their substantial antimycobacterial activity, as indicated by previous research [13]. The heterocyclic ring template pyrrole, which has a variety of pharmacophores, makes it possible to create a library of enormous lead compounds. The pyrroles-based lead analogue, LL3858 (Fig 1) [14], is presently undergoing phase IIa clinical investigations in India. Lupin Limited firstly revealed the MIC range for LL3858 against Mtb in 2004. It varied from 0.05 to 0.1 μg/mL.

A structural characteristic of the pyrrole ring with advantageous electron characteristics is probably the cause of the improved interaction with enzymes and receptors. To attain the ideal activity profile, this element in the scaffold provides potential for further modification. Further research revealed that compounds with pyrrole based core structures exhibited a range of biological activities, including antimicrobial [15], antiviral [16], anticancer [17], and antimycobacterial [18–20] properties.

Dihydrofolate reductase (DHFR) catalyzes the reduction of dihydrofolate to tetrahydrofolate which couples with thymidylate synthase in the reductive methylation of deoxyuridine to deoxythymidine. Tetrahydrofolate cofactor deficiencies brought on by the suppression of DHFR function result in cell death. As a key target for the development of chemotherapeutic agents against bacterial and parasite diseases as well as TB, DHFR inhibition has long been recognized. Methotrexate and trimethoprim are examples of DHFR inhibitors used in the clinic.

Fatty acid production inhibition is a desirable target for the rational creation of novel anti-tubercular drugs among the various Mycobacteria targets being investigated for antitubercular action. The main element of the cell wall of Mtb is mycolic acid. For the development of new antimycobacterial drugs, fatty acid biosynthesis-related enzymes are regarded as the optimal targets. The fatty acid synthase enzymes FAS-I and FAS-II catalyse the production of fatty acids. In mammals, FAS-I catalyses the synthesis; in Mycobacterium, FAS-I and FAS-II do the catalyzing. Due to this distinction, FAS-II is a desirable target for the development of antitubercular drugs. The FAS-II system contains an essential enzyme known as enoyl-ACP (CoA) reductase (FabI/ENR/InhA) [21]. Isoniazid, the most recommended anti-tubercular drug, targets the inhA structural gene in Mtb as its main target. An enoyl-ACP (CoA) reductase with the specificity for chain elongation in mycolic acid precursors, InhA, was discovered [22]. Different classes of direct inhibitors of InhA enzyme were investigated such as Triclosan and derivatives, GEQ analogues etc.

Our laboratory for Novel Drug Design and Discovery has been conducting research on the potential of DHFR and enoyl-ACP reductase as molecular targets for antitubercular therapies. Specifically, we have been investigating the use of pyrrole pharmacophoric scaffolds that inhibit both DHFR and enoyl-ACP reductase. The aim of our research is to develop novel compounds that can effectively inhibit both targets, thereby exhibiting antitubercular effects.

We have reported the synthesis of pyrrole compounds as antitubercular drugs and enoyl ACP reductase inhibitors in the prior studies [23–26].

In this study, we present the design and synthesis of 20 bioactive pyrrole scaffold-containing dual-target inhibitors of DHFR and enoyl-ACP reductase. Such inhibitors could get around the toxicity, drug-drug interactions, and/or pharmacokinetic drawbacks of using two different medications in combination chemotherapy treatments. Additionally, the price of a single medication may be less than the cost of two different treatments, and it may also increase patient compliance.

## 2. Results and discussion

**Fig 2:**
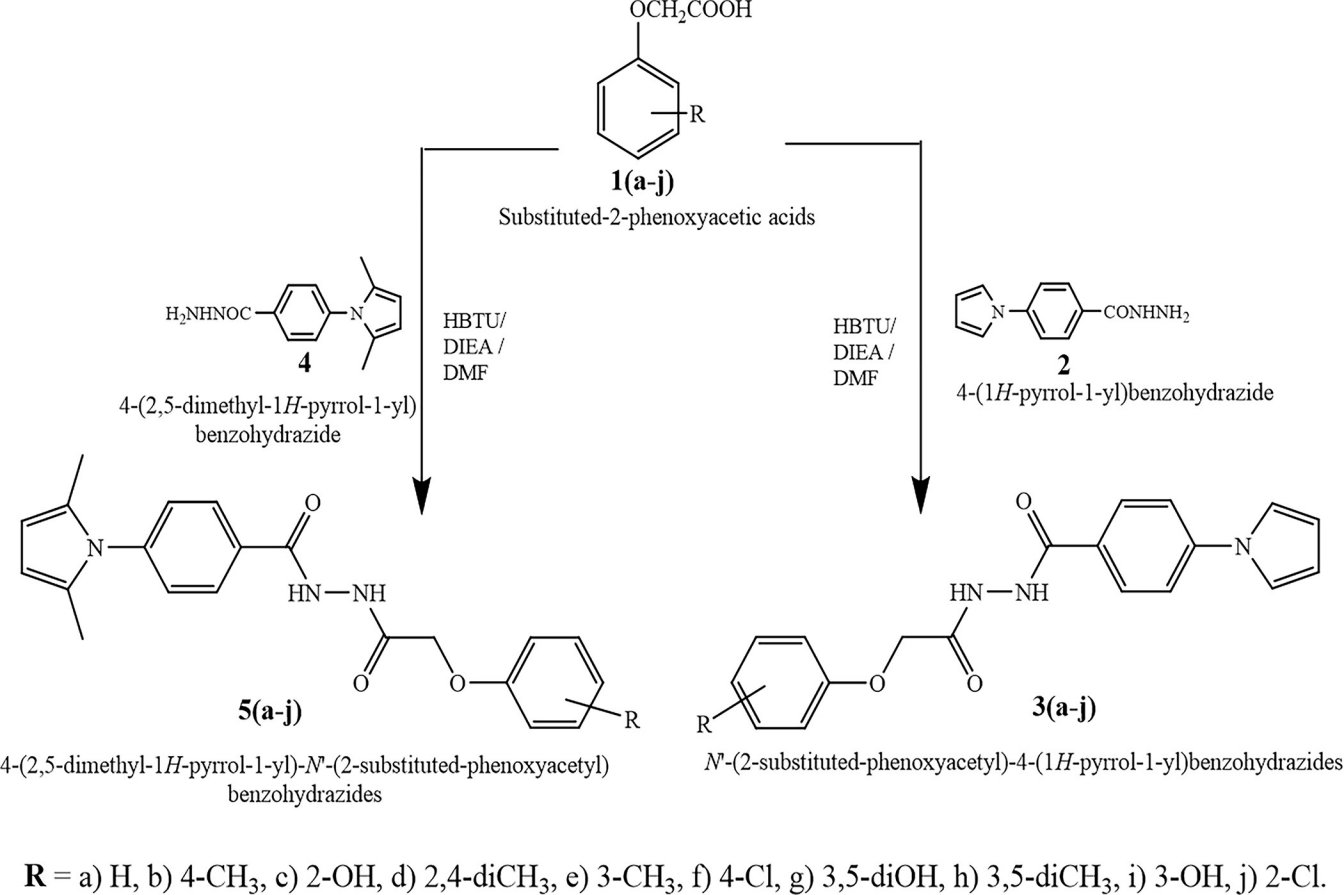
The scheme-1 outlines a synthetic pathway for the production of innovative derivatives of pyrrolyl-benzohydrazides.

According to the methodology described in Scheme 1, the target compounds, specifically 4-(1H-pyrrol-1-yl)-N’-(2-(substitutedphenyloxy)acetyl)benzohydrazides (3a-j) and 4-(2,5-dimethyl-1H-pyrrol-1-yl)-N’-(2-(substitutedphenoxy)acetyl)benzohydrazides(5a-j), were synthesized (Fig 2). This synthesis involved the reaction of 4-pyrrol-1-yl benzoic acid hydrazide 2 or 4-(2,5-dimethyl pyrrol-1-yl)benzohydrazide 4 with substituted phenoxy acetic acids in distilled N’,N’-dimethyl formamide. The reaction was facilitated by the coupling agent 2-(1H-benzotriazol-1-yl)-1,1,3,3-tetramethyluronium hexafluorophosphate and N’,N’-diisopropylethylamine, which acted as a catalytic agent. The reaction was carried out under cold conditions. The structural confirmation of newly reported pyrrolyl-benzohydrazides was achieved by the analysis of spectral data.

**Fig 3:**
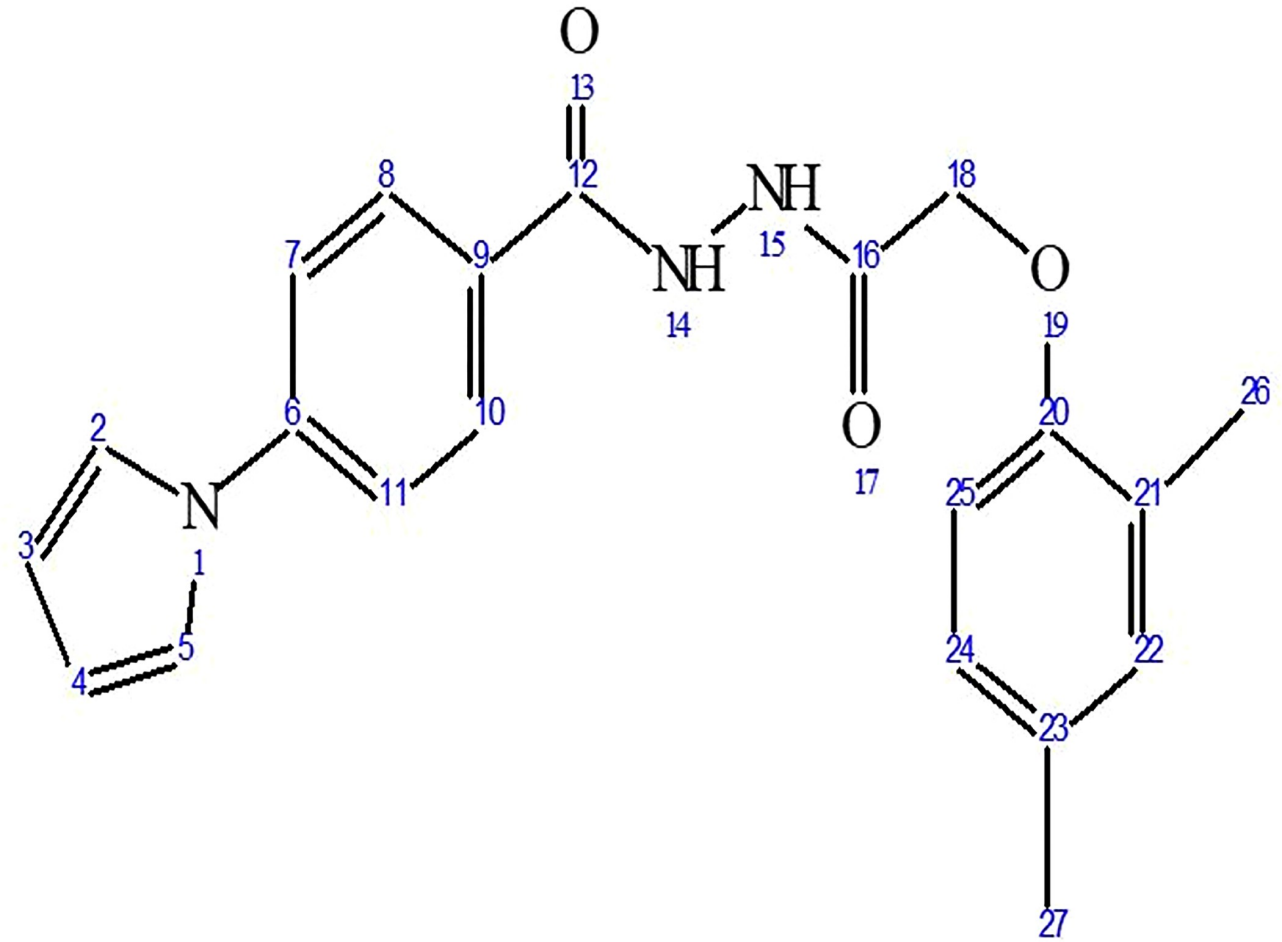
The compound 3d- structural confirmation.

In the ^1^H NMR spectrum of compound **3d**, proton of two -NH appeared as singlets at 10.49 and 10.14 *δ* ppm, two protons of phenyl-C_8_, C_10_ and two protons ofbridging phenyl-C_7_, C_11_ appeared as two doublets at 8.02 *δ* and 7.76 *δ* ppm. Similarly, two protons of pyrrole-C_2_, C_5_ and two protons of phenyl-C_22_, C_24_ appeared as two doublets at 7.51 *δ* and 7.04 *δ* ppm respectively. The proton signals of pyrrole-C_3_ and C_4_ were observed as a triplet at a chemical shift range of 6.33–6.32 *δ* ppm, while the six methyl protons were observed as a singlet at a chemical shift of 2.22 *δ* ppm. The identification of the CH_2_ group was confirmed by the observation of a singlet signal at 4.65 δ ppm. The ^13^C nuclear magnetic resonance (NMR) spectrum of compound **3d** exhibited distinct peaks at 167.44 and 164.61 δ ppm, indicating the presence of two different carbonyl groups. Additionally, shifts corresponding to pyrrole and phenyl carbons were observed throughout the estimated range of δ ppm from 111.19 to 142.27. Moreover, the compound **3d**- structural confirmation was achieved through the examination of its mass spectrum. This analysis revealed a peak at m/z 365.26 (M^+^+2), which corresponds to the compound’s molecular weight calculated from expected molecular formula (Fig 3).

### 2.1. Molecular docking

**Fig 4:**
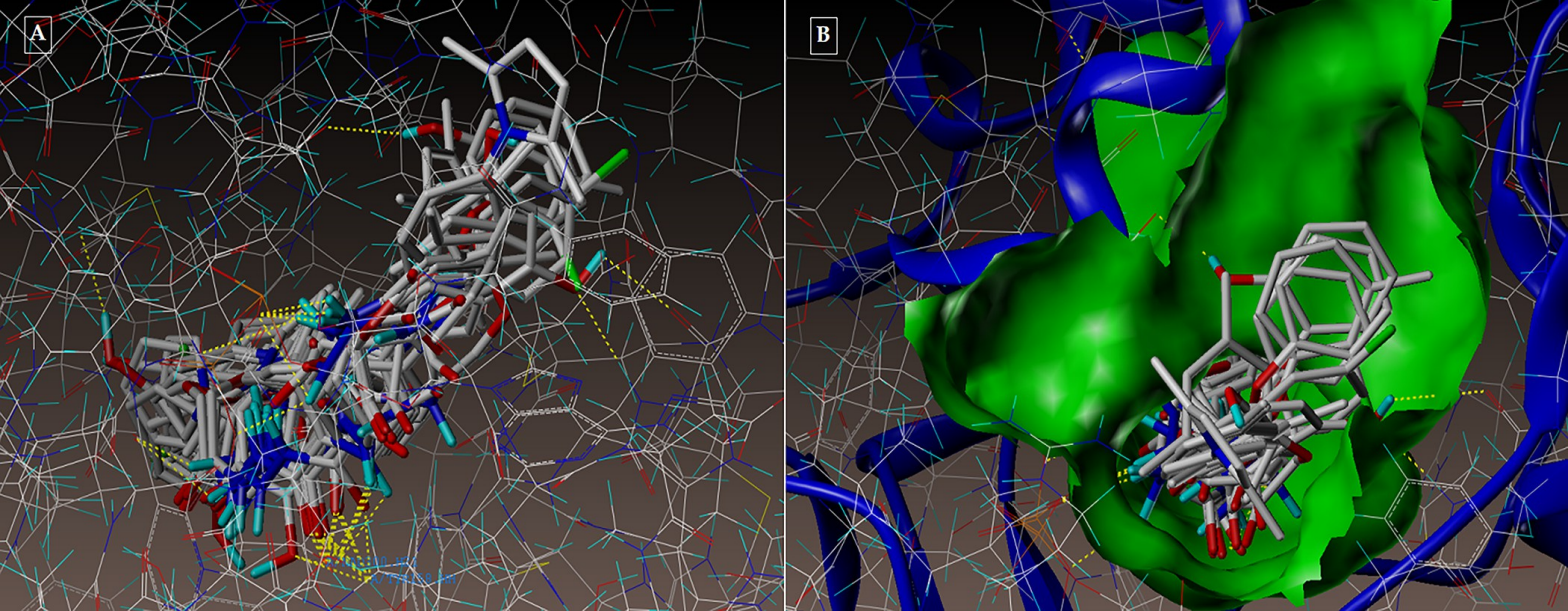
(A-B): Depicting the docked mode of all the compounds within the hypothesized binding pocket of InhA, as represented by PDB: 2NSD.

**Fig 5:**
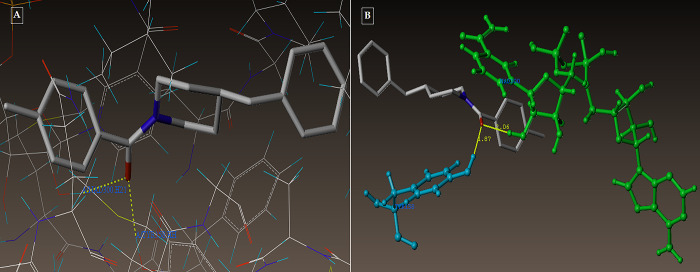
**(A-B):** depicting the 2NSD_ligand docked mode at InhA (A) and 2NSD_ligand 3D docked view (B). Tyr 158, a residue at the binding site, is colored cyan, NAD+ is colored green, and the molecule is colored according to the type of atom.

**Fig 6:**
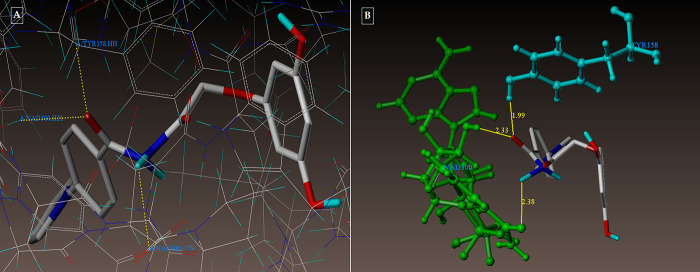
(A-B): (A) Compound 3g docked mode; (B) 3D docked view of the compound 3g. Binding site residues are cyan-colored Tyr 158 amino acid, green-colored co-factor NAD+, and the molecule is colored by atom type.

**Fig 7:**
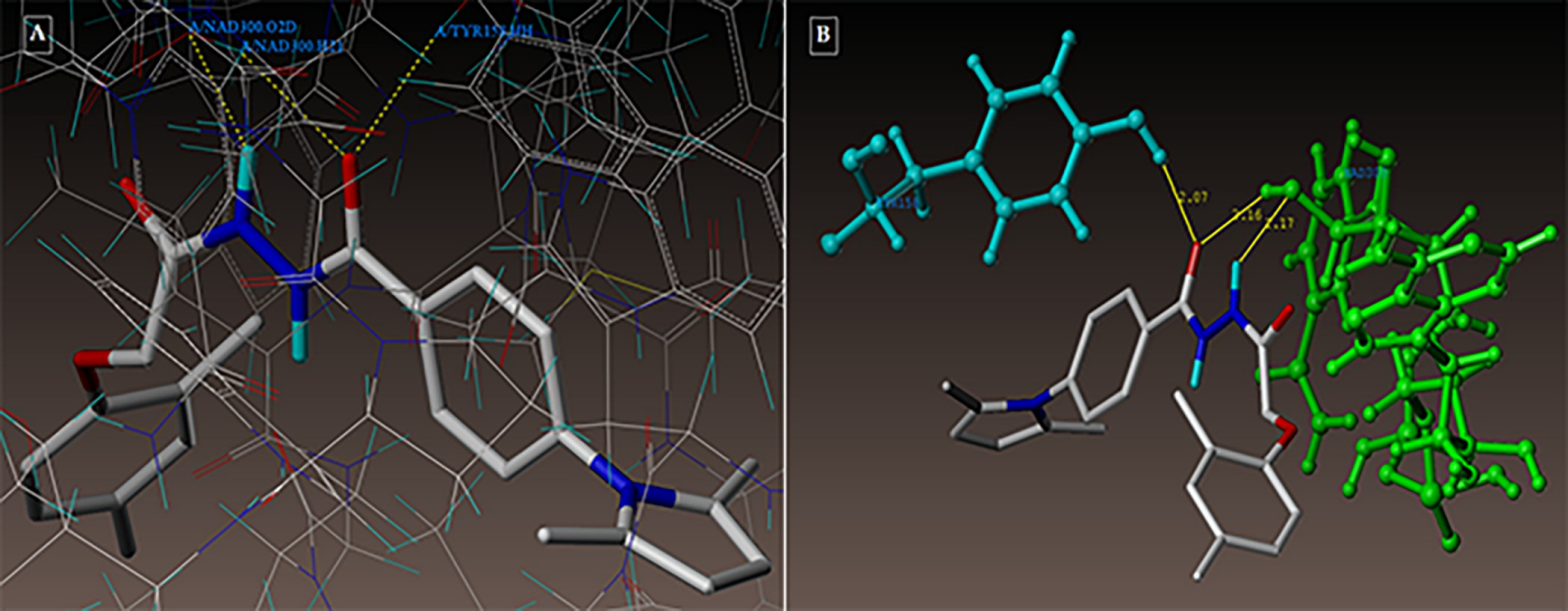
(A-B): (A) Compound 5d in docked mode; (B) Compound 5d in 3D docked view. Tyr 158, a residue at the binding site, is colored cyan, NAD+ is colored green, and the molecule is colored according to the type of atom.

**Fig 8:**
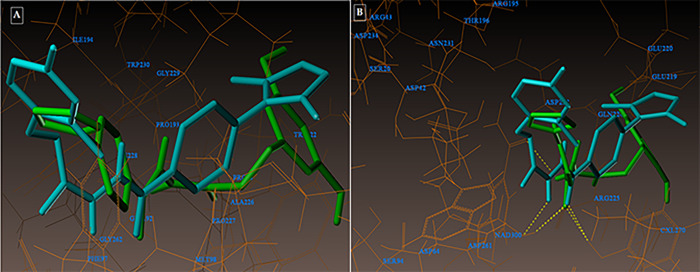
**(A-B):** depicting the hydrophobic and hydrophilic amino acids surrounding the compounds 3g and 5d under consideration.

The active pocket was found to be the area of the complex between the 2NSD_ligand and the 2NSD enoyl-ACP reductase. It was re-docked in order to get the contacts and orientation of the 2NSD_ligand at the active site for comparison with other synthesized compounds (Fig 4A and 4B). The synthetic molecule **3g** binds to the same binding site as 2NSD_ligand, according to the Surflex-Dock docking studies. The oxygen of the carbonyl group in the 2NSD_ligand has shown two H-bond interactions, creating two hydrogen bonds with the OH of the active sites of NAD+ ribose (2.06) and TYR158 (1.87) (Fig 5A and 5B). With the amino acid Tyr 158 and the co-factor NAD+, respectively, the oxygen atom of the carbonyl group has formed two H-bonding connections in the molecules **3g** and **5d** (Figs 6A, 6B, 7A and 7B). Fig 8A and 8B depict the hydrophobic and hydrophilic amino acids that are encircled by the two compounds **3g** and **5d**.

The consensus scores obtained for the compounds, which varied from 8.74 to 5.38, highlight the essentiality of all intermolecular interactions between the ligands and the InhA protein. The electrostatic and van der Waals forces between the protein and ligands exhibited a range of values spanning from -150.08 to -139.12. The Helmholtz free energies of interactions for atom pairs between proteins and ligands exhibited a range of values from -63.09 to -39.60. Additionally, the energies associated with H-bonding, complex formation between the ligand and protein, and internal interactions within the ligand itself ranged from -250.95 to -212.47. Furthermore, values ranging from -46.92 to -32.70 were observed for the ligands, indicating the presence of H-bonding, lipophilic contact, and rotational entropy. The compounds have a preferential binding affinity towards InhA, as indicated by the observed outcomes and supporting references. The 2NSD ligand is a molecule that binds to a specific receptor or protein (Table 1).

**Table 1:** Surflex Docking score in kcal/mol for pyrrole derivatives on the PDB ID: 2NSD.

Compounds	Total Score^a^	Crash Score^b^	Polar Score^c^	D score^d^	PMF Score^e^	G Score^f^	Chem Score^g^
**2NSD_ligand**	9.25	-0.93	1.54	-150.083	-63.091	-250.959	-46.922
3g	8.74	-1.25	1.05	-146.022	-27.104	-225.371	-37.314
5d	8.49	-2.04	1.70	-149.781	-87.633	-280.534	-42.817
3a	8.41	-1.27	1.93	-133.435	-75.786	-254.546	-37.520
3h	7.36	-1.77	1.88	-161.388	-75.365	-260.265	-39.781
3i	7.33	-1.40	3.39	-126.626	-87.862	-199.298	-37.617
5g	7.20	-1.32	2.98	-152.376	-72.938	-232.286	-42.754
3e	7.20	-1.43	1.92	-128.682	-73.370	-236.563	-37.357
3b	7.17	-1.75	1.74	-132.318	-76.891	-255.492	-36.836
3c	7.03	-1.60	1.36	-136.394	-89.992	-246.311	-40.328
3d	6.82	-1.25	1.69	-157.030	-67.521	-254.255	-41.866
5e	6.73	-1.57	2.18	-134.748	-87.875	-215.834	-32.587
3f	6.61	-0.97	0.99	-132.540	-70.899	-222.874	-36.092
5f	6.56	-1.20	2.09	-149.617	-68.155	-231.933	-43.693
3c	6.50	-1.50	1.96	-127.158	-82.426	-220.411	-35.503
5c	6.32	-1.78	0.61	-144.670	-41.953	-269.401	-33.218
5i	6.27	-1.32	1.95	-117.248	-68.432	-203.390	-30.528
5h	6.23	-1.66	0.03	-145.411	-36.648	-218.277	-33.512
5b	6.18	-1.30	1.86	-149.846	-68.844	-233.856	-40.982
5c	6.10	-2.41	1.75	-145.601	-35.947	-248.880	-38.471
5a	5.38	-1.92	0.10	-139.123	-39.609	-212.476	-32.706

^a^CScore The Consensus Score algorithm combines various widely used scoring algorithms to rank the affinity of ligands that are coupled to the active site of a receptor. It then provides the overall score as the output.

^b^Crash-score- The crash-score reveals the improper penetration into the binding point. Crash scores close to 0 are considered positive. Penetration is indicated by negative values.

^c^Polar- denotes the contribution of polar interactions to the overall score. The polar score may be beneficial for filtering out docking findings that do not form any hydrogen bonds.

^d^D-score- Charge and van der Waals interactions between the protein and the ligand are given a D-score.

^e^ PMF-score- The Helmholtz free energy of interactions for protein-ligand atom pairs are indicated by the PMF-score (Potential of Mean Force, PMF).

^f^G-score- The G-score demonstrates hydrogen bonding, complex (ligand-protein), and internal (ligand-ligand) energies.

**Fig 9:**
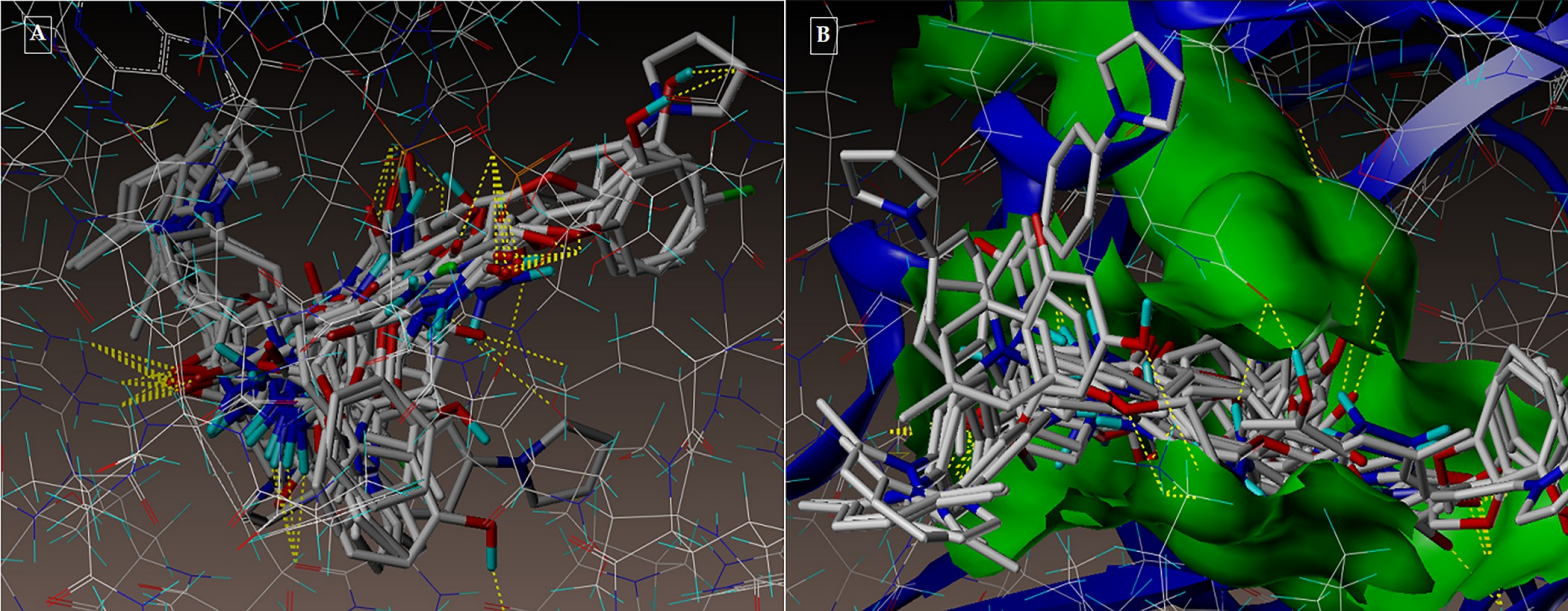
(A-B): The docking mechanism of all the synthesized compounds within the proposed binding pocket of InhA (PDB: 1DF7).

All of the compounds had excellent docking scores (Fig 9A and 9B) against the dihydrofolate reductase forms of Mycobacterium TB, according to a second docking investigation using PDB ID: 1DF7.

**Fig 10:**
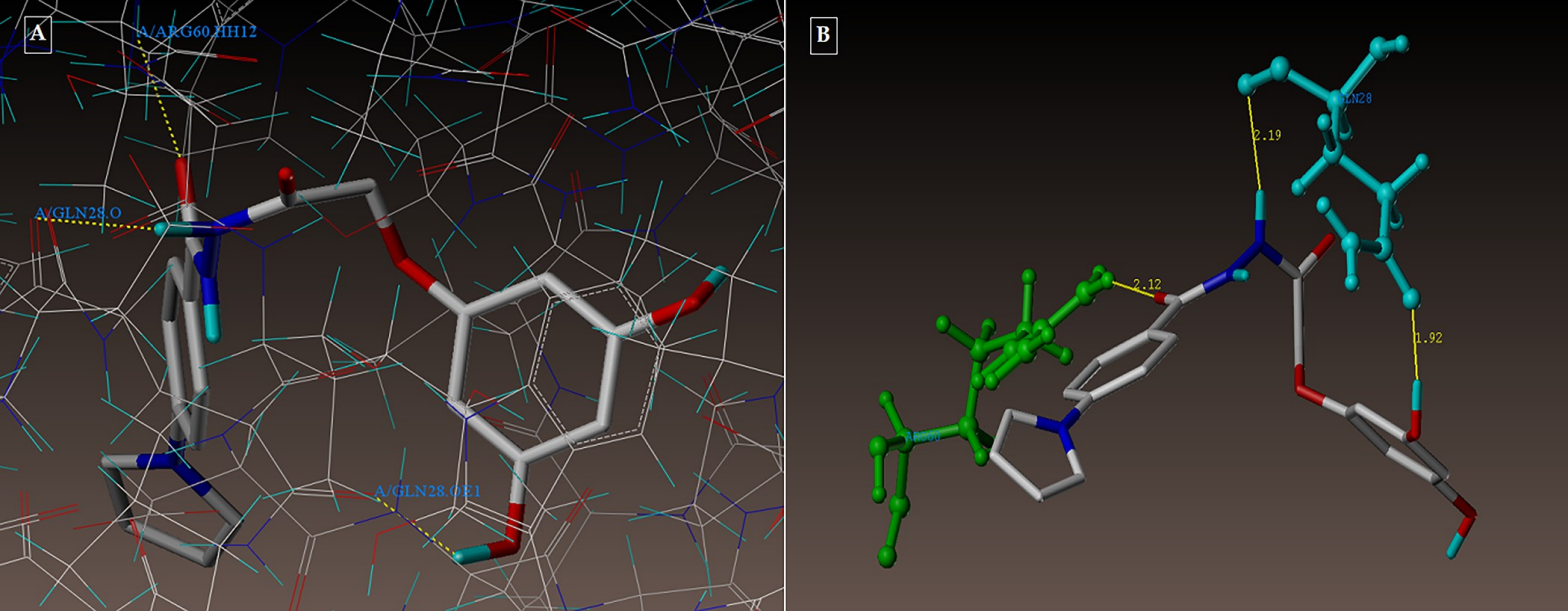
(A-B): (A) Compound 3g docked mode; (B) Compound 3g 3D-docked view. Binding site residues include cyan-colored GLN28 amino acids, green-colored ARG60 amino acids, and a molecule that is colored according to atom type.

**Fig 11:**
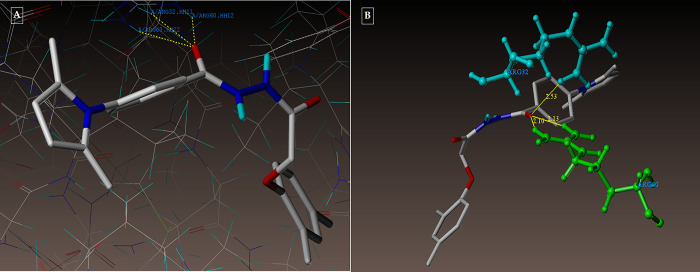
(A-B): (A) Compound 5d docked mode; (B) Compound 5d 3D-docked view. Binding site residues: cyan ARG32 amino acid, green ARG60, and the molecule is colored according to atom type.

**Fig 12:**
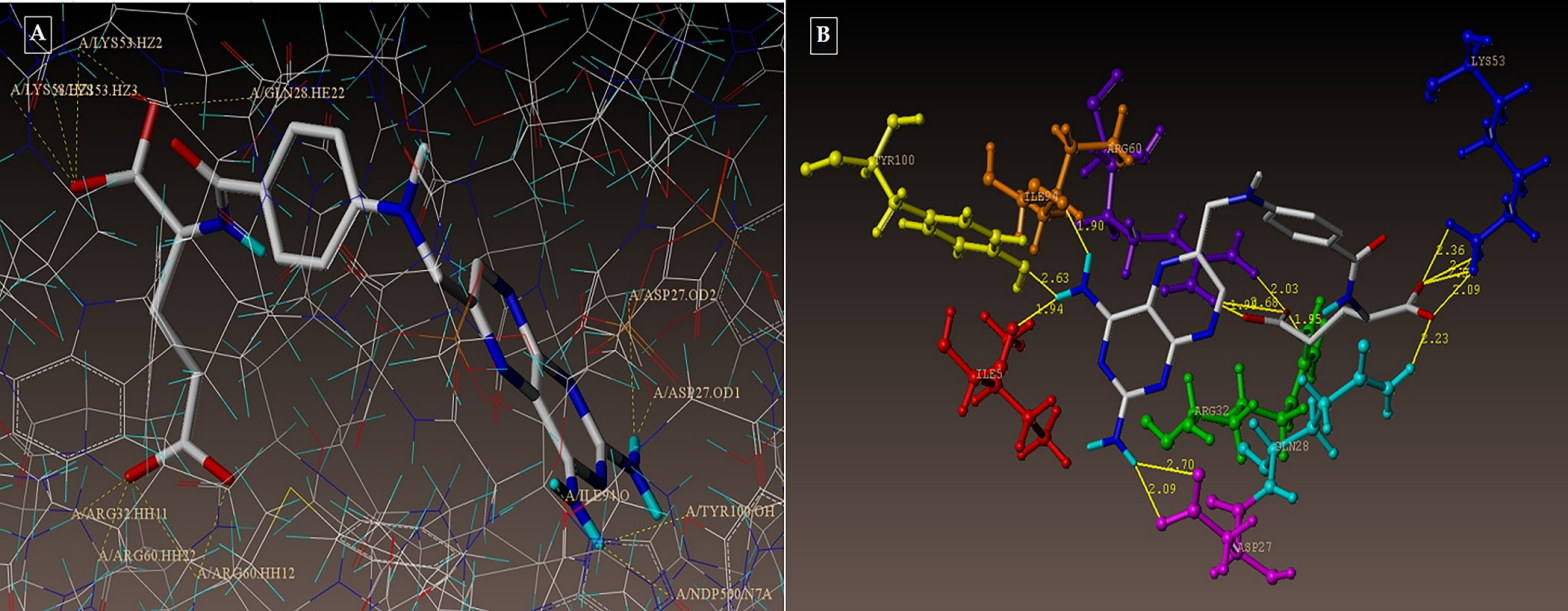
(A-B): (A) 1DF7_ligand docked mode at InhA; (B) 3D-Docked view of 1DF7_ligand.

**Fig 13:**
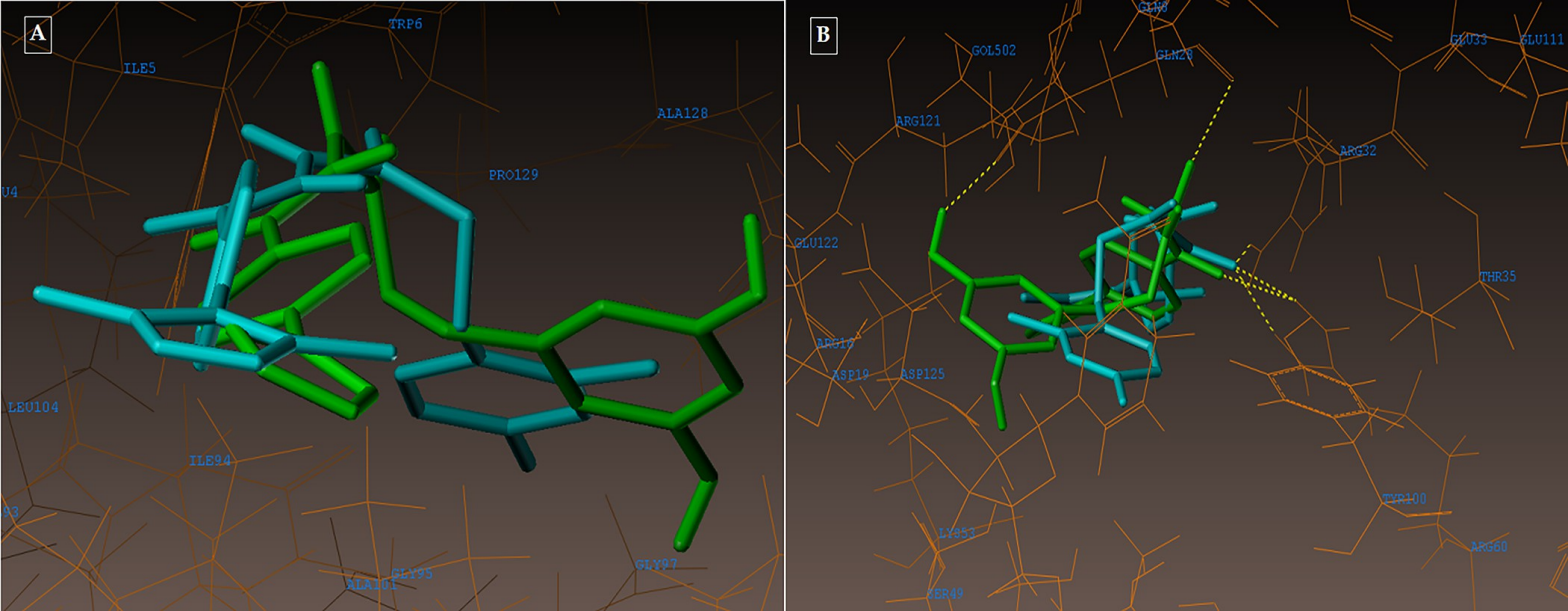
(A-B): depicts hydrophobic and hydrophilic amino acids encompassed by the investigated compounds 3g and 5d.

Compound **3g** forms three hydrogen bonding contacts at the enzyme’s active site, as depicted in Fig 9A and 9B (PDB ID: 1DF7). ARG60’s hydrogen atom is involved in one hydrogen bond raised by the oxygen of the benzohydrazide’s C = O group (-O—H-ARG60, 2.12 Å), while GLN28’s oxygen atom is involved in another (-H—-O- GLN28, 2.19 Å) by the NH group. As seen in Fig 10A and 10B (PDB ID: 1DF7), compound **5d** creates three hydrogen bonds in the enzyme’s active site. These bonds are formed by the hydrogen atoms of ARG60 and ARG32 with the oxygen atom of the benzohydrazide’s C = O group (-O—H-ARG60, 2.10 Å, 2.13; O—H-ARG32, 2.53 Å***)*.** The docked picture of the binding interaction of 1DF7_ligand (methotrexate) with enzyme active sites in Figs 11, 12A and 12B) shows 14 bonding connections. Fig 13A and 13B depict the hydrophobic and hydrophilic amino acids that are encircled by the two compounds **3g** and **5d**.

Table 2 provides a comprehensive summary of the intermolecular forces governing the interaction between ligands and the enzyme. It is noteworthy that all the compounds examined in this study had consensus scores ranging from 7.32 to 5.17. We also noticed that the examined compounds interacted with the amino acid residues (ARG60, ARG32, and GLN28) similarly to the 1DF7_ligand. This implies that molecules engage in comparable interactions with enzymes as ligands do.

**Table 2:** Surflex Docking score in kcal/mol for pyrrole compounds on PDB ID 1DF7.

Compounds	C Score^a^	Crash Score^b^	Polar Score^c^	D Score^d^	PMF Score^e^	G Score^f^	Chem Score^g^
**Ligand**	13.76	-1.32	8.92	-229.875	-138.104	-353.514	-38.494
3g	7.32	-2.07	1.20	-109.823	-53.987	-281.749	-29.195
3d	7.12	-1.77	2.19	-97.018	-33.707	-203.414	-27.765
5d	6.94	-1.54	2.30	-81.176	-71.600	-196.869	-29.565
5h	6.81	-3.49	0.91	-162.681	-55.882	-287.213	-35.102
5e	6.60	-0.71	2.09	-73.410	-73.493	-165.511	-27.557
5c	6.51	-1.91	2.56	-106.916	-58.777	-266.682	-29.689
5g	6.41	-1.49	2.13	-112.100	-74.030	-195.563	-27.656
5b	6.37	-1.05	2.08	-79.071	-74.378	-187.752	-27.485
5a	6.33	-1.83	1.13	-104.436	-48.028	-215.500	-32.044
5f	6.19	-0.86	2.22	-78.012	-70.320	-175.642	-28.680
3j	6.07	-1.30	1.92	-139.635	-49.127	-203.228	-28.440
3a	5.83	-1.35	1.06	-100.241	-48.084	-205.159	-27.672
3i	5.79	-1.29	2.30	-94.615	-63.104	-192.237	-28.573
5j	5.68	-1.52	1.93	-85.746	-61.964	-214.416	-28.200
3c	5.57	-1.26	1.06	-102.870	-58.912	-201.403	-28.472
3h	5.37	-1.25	0.85	-126.595	-58.762	-211.441	-28.895
3b	5.28	-1.42	1.64	-79.274	-40.664	-192.858	-25.161
3e	5.27	-1.71	0.30	-139.544	-53.423	-215.774	-30.484
5i	5.22	-1.82	2.91	-65.090	-51.556	-152.408	-22.674
3f	5.17	-1.61	1.90	-103.230	-49.151	-214.318	-27.011

### 2.2. Antitubercular and antibacterial activities

The findings of the antimicrobial study conducted on *S*. *aureus* (Gram-positive) and *E*. *coli* (Gram-negative) are presented in Table 3, as well as the results of the antitubercular testing of all substances against the *M*. *tuberculosis* H37Rv strain utilizing the MABA method. Its acronym is MIC, and it is used to describe a compound’s specific activities. The compounds displayed good antitubercular activity between 1.6 and 12.5 μg/ml, according to the preliminary antitubercular studies. At MIC 3.12 μg/ml, compounds **3c, 3d, 3g, 3h, 5f, 5g, 5h,** and **5j** displayed greater activity. At MIC 1.6 μg/ml, compounds **5d** and **5e** exhibited the highest level of activity. Compounds shown antibacterial activity (given as MIC) between 1.6 and 100 μg/ml.

**Table 3:** Newly synthesized pyrrole compounds’ preliminary *in vitro* antibacterial, antitubercular, MtDHFR, and enoyl ACP reductase inhibition values.

Compound	*M*. *tuberculosis* H_37_Rv MIC values in μg ml^-1^	*S*. *aureus (*Gram +ve)	*E*. *coli* (Gram–ve)	IC_50_ (μM) MtDHFR	% Inhibition of InhA at 50 μM
		MIC *(*μg/mL*)*	MIC *(*μg/mL*)*		
**3a**	12.5 (37.27)	100 (298.18)	6.25 (99.70)	-	-
**3b**	12.5 (35.77)	100 (286.21)	6.25 (17.88)	-	-
**3c**	3.12 (8.87)	100 (284.60)	3.12 (8.87)	25	46
**3d**	3.12 (8.58)	100 (275.16)	1.6 (4.40)	32	48
**3e**	6.25 (17.88)	100 (286.21)	12.5 (35.77)	-	-
**3f**	6.25 (16.90)	100 (270.40)	12.5 (33.80)	122	-
**3g**	3.12 (8.49)	100 (272.21)	1.6 (4.35)	20	63
**3h**	3.12 (8.58)	100 (275.16)	3.12 (8.58)	19	59
**3i**	12.5 (35.57)	100 (284.60)	12.5 (35.57)	-	-
**3j**	12.5 (33.80)	100 (270.40)	6.25 (16.90)	-	-
**5a**	6.25 (17.19)	100 (275.16)	6.25 (17.19)	98	26
**5b**	12.5 (33.11)	100 (264.94)	12.5 (33.11)	-	-
**5c**	12.5 (32.94)	100 (263.56)	12.5 (32.94)	-	-
**5d**	1.6 (4.09)	100 (255.63)	3.12 (7.97)	56	62
**5e**	1.6 (4.23)	100 (264.94)	3.12 (8.26)	62	66
**5f**	3.12 (7.84)	100 (251.34)	6.25 (15.70)	-	-
**5g**	3.12 (7.89)	100 (252.89)	3.12 (7.89)	-	-
**5h**	3.12 (7.6)	100 (255.44)	3.12 (7.6)	-	-
**5i**	6.25 (16.47)	100 (263.56)	3.12 (8.22)	-	-
**5j**	3.12 (7.84)	100 (251.34)	6.25 (15.70)	-	-
**Pyrazinamide**	3.12	-	-		-
**Streptomycin**	6.25	-	-		-
**Ciprofloxacin**	-	2	2		-
**TMP**	-	-	-	92	-
**TCL**	-	-	-	-	>99

Results are expressed as % InhA inhibition.

### 2.3. MtDHFR inhibitory activity

By observing the fluorescence given off by MtDHFR substrates when stimulated at 340 nm, the *in vitro* MtDHFR inhibitory activity of our newly synthesized compounds was determined (**Table 3**). Since the product (NADP+) is not luminescent, the enzyme’s activity was evaluated by consuming its substrate. Trimethoprim, the assay’s positive control, had an IC_50_ value of 92 μM using this method, which was consistent with the findings from the literature (88 μM). **Table 3** shows that the majority of the investigated compounds were much more effective against MtDHFR. Six of them (**3c, 3d, 3g, 3h, 5d,** and **5e**) had greater inhibition characteristics than trimethoprim.

### 2.4. ADME studies

The Swiss ADME web tool calculated the ADME properties of every synthesised compound, and every molecule complies with Lipinki’s rule of five. All compounds have good synthetic accessibility, moderate solubility, and GI absorption, according to Swiss ADME tests. The BBB is only not crossed by compounds **3c**, **3g**, **3i**, **5c**, **5g** and **5i**. The substances had skin permeability that ranged from -5.61 to -7.06, which was moderate. **Table 4** presents the outcomes.

**Table 4:** Swiss ADME web tool’s ADME properties for synthetic molecules.

Compound	Log P	Molar refractivity	TPSA	HBA	HBD	RB	GI Absorption	BBB Permeant	Log Kp cm/s	Solubility	CYP inhibitor					Lipinski violation	Synthetic accessibility
											1A2	2C19	2C9	2D6	3A4		
**3a**	2.48	92.59	72.36	3	2	8	High	Yes	-6.36	Soluble	No	Yes	Yes	Yes	No	0	2.43
**3b**	2.53	97.55	72.36	3	2	8	High	Yes	-6.18	Soluble	No	Yes	Yes	Yes	Yes	0	2.55
**3c**	1.47	94.61	92.59	4	3	8	High	No	-6.70	Soluble	No	No	No	Yes	No	0	2.53
**3d**	2.78	102.52	72.36	3	2	8	High	Yes	-6.01	Moderately Soluble	No	Yes	Yes	Yes	Yes	0	2.75
**3e**	2.75	97.55	72.36	3	2	8	High	Yes	-6.18	Soluble	Yes	Yes	Yes	Yes	Yes	0	2.56
**3f**	2.96	97.60	72.36	3	2	8	High	Yes	-6.12	Moderately Soluble	Yes	Yes	Yes	Yes	Yes	0	2.48
**3g**	1.89	96.63	112.82	5	4	8	High	No	-7.06	Soluble	No	No	No	Yes	No	0	2.60
**3h**	2.92	102.52	72.36	3	2	8	High	Yes	-6.01	Moderately Soluble	No	Yes	Yes	Yes	Yes	0	2.68
**3i**	1.63	94.61	92.59	4	3	8	High	No	-6.70	Soluble	No	No	No	Yes	No	0	2.57
**3j**	2.55	97.60	72.36	3	2	8	High	Yes	-6.12	Moderately Soluble	Yes	Yes	Yes	Yes	Yes	0	2.57
**5a**	2.56	102.52	72.36	3	2	8	High	Yes	-5.95	Moderately Soluble	No	Yes	Yes	Yes	Yes	0	2.74
**5b**	2.77	107.28	72.36	3	2	8	High	Yes	-5.78	Moderately Soluble	No	Yes	Yes	Yes	Yes	0	2.85
**5c**	2.33	104.54	92.59	4	3	8	High	No	-6.31	Moderately Soluble	No	No	Yes	Yes	No	0	2.83
**5d**	3.62	112.45	72.36	3	2	8	High	Yes	-5.61	Moderately Soluble	No	Yes	Yes	Yes	Yes	0	3.05
**5e**	2.66	107.48	72.36	3	2	8	High	Yes	-5.78	Moderately Soluble	No	Yes	Yes	Yes	Yes	0	2.87
**5f**	3.21	107.53	72.36	3	2	8	High	Yes	-5.72	Moderately Soluble	Yes	Yes	Yes	Yes	Yes	0	2.77
**5g**	2.62	106.56	112.82	5	4	8	High	No	-6.65	Moderately Soluble	Yes	No	Yes	Yes	No	0	2.90
**5h**	3.24	112.45	72.36	3	2	8	High	Yes	-5.61	Moderately Soluble	No	Yes	Yes	Yes	Yes	0	2.99
**5i**	2.65	104.54	92.59	4	3	8	High	No	-6.31	Moderately Soluble	No	No	Yes	Yes	No	0	2.86
**5j**	2.74	107.53	72.36	3	2	8	High	Yes	-5.72	Moderately Soluble	No	Yes	Yes	Yes	Yes	0	2.86

ProTox-II determined the toxicological summaries of all the chemicals, and **Table 5** shows the toxic effects. This data on toxicity demonstrated that none of the molecules shown any toxicity.

**Table 5:** Toxicity studies of synthesized compounds.

Compound code	LD50 mg/kg	Hepatotoxicity	Carcinogenicity	Immunotoxicity	Mutagenicity	Cytotoxicity	Aryl hydrocarbon Receptor	Androgen Receptor (AR)	Androgen Receptor Ligand Binding Domain	Aromatase	Estrogen Receptor Ligand Binding Domain	Peroxisome Proliferator Activated Receptor Gamma	Nuclear factor	Heat shock factor response element	Mitochondrial Membrane Potential	Phosphoprotein	ATPase family AAA domain containing protein 5
**3a**	400	Active	Active	Inactive	Inactive	Inactive	Inactive	Inactive	Inactive	Inactive	Inactive	Inactive	Inactive	Inactive	Inactive	Inactive	Inactive
**3b**	400	Active	Active	Inactive	Inactive	Inactive	Inactive	Inactive	Inactive	Inactive	Inactive	Inactive	Inactive	Inactive	Inactive	Inactive	Inactive
**3c**	375	Active	Inactive	Inactive	Inactive	Inactive	Inactive	Inactive	Inactive	Inactive	Inactive	Inactive	Inactive	Inactive	Inactive	Inactive	Inactive
**3d**	400	Active	Active	Inactive	Inactive	Inactive	Inactive	Inactive	Inactive	Inactive	Inactive	Inactive	Inactive	Inactive	Inactive	Inactive	Inactive
**3e**	400	Active	Active	Inactive	Inactive	Inactive	Inactive	Inactive	Inactive	Inactive	Inactive	Inactive	Inactive	Inactive	Inactive	Inactive	Inactive
**3f**	375	Active	Inactive	Inactive	Inactive	Inactive	Inactive	Inactive	Inactive	Inactive	Inactive	Inactive	Inactive	Inactive	Inactive	Inactive	Inactive
**3g**	1000	Active	Inactive	Inactive	Inactive	Inactive	Inactive	Inactive	Inactive	Inactive	Inactive	Inactive	Inactive	Inactive	Inactive	Inactive	Inactive
**3h**	400	Active	Inactive	Inactive	Inactive	Inactive	Inactive	Inactive	Inactive	Inactive	Inactive	Inactive	Inactive	Inactive	Inactive	Inactive	Inactive
**3i**	1000	Active	Inactive	Inactive	Inactive	Inactive	Inactive	Inactive	Inactive	Inactive	Inactive	Inactive	Inactive	Inactive	Inactive	Inactive	Inactive
**3j**	375	Active	Inactive	Inactive	Inactive	Inactive	Inactive	Inactive	Inactive	Inactive	Inactive	Inactive	Inactive	Inactive	Inactive	Inactive	Inactive
**5a**	1600	Active	Active	Inactive	Inactive	Inactive	Inactive	Inactive	Inactive	Inactive	Inactive	Inactive	Inactive	Inactive	Inactive	Inactive	Inactive
**5b**	1600	Active	Inactive	Inactive	Inactive	Inactive	Inactive	Inactive	Inactive	Inactive	Inactive	Inactive	Inactive	Inactive	Inactive	Inactive	Inactive
**5c**	475	Active	Inactive	Inactive	Inactive	Inactive	Inactive	Inactive	Inactive	Inactive	Inactive	Inactive	Inactive	Inactive	Inactive	Inactive	Inactive
**5d**	475	Active	Inactive	Inactive	Inactive	Inactive	Inactive	Inactive	Inactive	Inactive	Inactive	Inactive	Inactive	Inactive	Inactive	Inactive	Inactive
**5e**	475	Active	Inactive	Inactive	Inactive	Inactive	Inactive	Inactive	Inactive	Inactive	Inactive	Inactive	Inactive	Inactive	Inactive	Inactive	Inactive
**5f**	278	Active	Inactive	Inactive	Inactive	Inactive	Inactive	Inactive	Inactive	Inactive	Inactive	Inactive	Inactive	Inactive	Inactive	Inactive	Inactive
**5g**	475	Active	Inactive	Inactive	Inactive	Inactive	Inactive	Inactive	Inactive	Inactive	Inactive	Inactive	Inactive	Inactive	Inactive	Inactive	Inactive
**5h**	475	Active	Inactive	Inactive	Inactive	Inactive	Inactive	Inactive	Inactive	Inactive	Inactive	Inactive	Inactive	Inactive	Inactive	Inactive	Inactive
**5i**	475	Active	Inactive	Inactive	Inactive	Inactive	Inactive	Inactive	Inactive	Inactive	Inactive	Inactive	Inactive	Inactive	Inactive	Inactive	Inactive
**5j**	1700	Active	Inactive	Inactive	Inactive	Inactive	Inactive	Inactive	Inactive	Inactive	Inactive	Inactive	Inactive	Inactive	Inactive	Inactive	Inactive

The study explores pyrrole-based compounds that act as dual inhibitors and their impact on current tuberculosis (TB) treatment, especially in relation to resistance mechanisms. TB is a significant global health issue, and the development of drug-resistant strains presents a significant obstacle to successful treatment. Approaching this challenge involves targeting multiple essential enzymes involved in the survival and replication of M. tuberculosis, the bacterium responsible for TB [14,19].

Dual inhibitors offer a significant advantage by targeting two distinct enzymes, enoyl ACP reductase and DHFR, essential for bacterial growth. By inhibiting both enzymes, dual inhibitors disrupt multiple metabolic pathways, making it more challenging for the bacterium to develop resistance through mutations in a single target.

Resistance mechanisms in TB usually result from mutations in the genes that encode the targeted enzymes, resulting in decreased drug binding affinity or changed enzyme activity. Using dual inhibitors can be beneficial in addressing resistance by providing a stronger defense against resistance development. Changes that provide resistance to one enzyme may not necessarily provide resistance to the other enzyme targeted by the dual inhibitor. By using this approach, the chances of the bacterium becoming resistant to the combination therapy are minimized, which could be a successful method to address drug-resistant strains [14,27].

In addition, the study’s molecular docking analysis offers valuable information on how the synthesized compounds interact with the active sites of enoyl ACP reductase and DHFR. By comprehending these interactions, we are able to guide the design and enhancement of upcoming dual inhibitors with improved effectiveness and specificity. Researchers have pinpointed crucial structural details that support dual inhibitory activity, paving the way for the development of more potent compounds against TB that are less susceptible to resistance [28].

The results of this study demonstrate the prospect of dual inhibitors as a potential approach in TB treatment. By focusing on multiple crucial enzymes and minimizing the chances of resistance emergence, dual inhibitors provide a valuable approach to address drug-resistant TB strains. This study highlights the significance of using multiple targets in drug design and lays the groundwork for creating new compounds with improved antibacterial and antitubercular properties.

## 3. Experimental section

### 3.1. Chemicals

Chemicals were procured from Spectrochem Pvt Ltd, Sigma Aldrich, and S. D. Fine Chem. Ltd for the purpose of synthesizing the mentioned compounds. Both the recrystallization procedure and the distillation method were employed for the purification of various compounds and solvents.

### 3.2. Instruments

The synthesized compounds’ melting points were determined using the SHITAL-Digital programmable melting point apparatus (SSI-22(B)) and occasionally with the Thiles Tube. These values are reported without any corrections. Infrared spectra were acquired using KBr pellets on the Bruker-T spectrophotometer. The chemical shifts in this study were quantified in terms of δ values (parts per million) for both the ^*1*^H NMR and ^*13*^C NMR analyses. These measurements were conducted using Bruker Avance IIINMR400/100 MHz equipment, with dimethylsulfoxide (DMSO-d6) serving as the solvent and tetramethylsilane (TMS) used as the internal standard. The nuclear magnetic resonance (NMR) spectra can be categorized into several signal patterns, including singlet (s), doublet (d), doublet of doublet (dd), triplet (t), quartet (q), and multiplet (m). The mass spectra of all the compounds obtained with the MS**-**Waters SynaptG2 instrument exhibited data that was consistent with the anticipated molecular structure. The experimental procedure involved the implementation of analytical thin layer chromatography (TLC) utilizing Silica Gel GF as the stationary phase, while the mobile phase’s progression was monitored through the utilization of an ultraviolet lamp.

### 3.3. General procedure for the synthesis of pyrrolylbenzohydrazide derivatives 3(a-j) and 5(a-j)

4-pyrrol-1-yl benzoic acid hydrazide 2/ 4-(2,5-dimethyl pyrrol-1-yl)benzohydrazide 4 [29] (0.0018 mol) was dissolved in 20 ml of dry DMF. Subsequently, in a low temperature environment, a solution containing 0.0019 moles of substituted-2-phenoxy acetic acids (1a-j) was introduced. Hexafluorophosphate benzotriazole urea (HBTU) (0.87 grams, 0.0023 moles) and diisopropylethylamine (DIEA) (0.93 milliliters, 0.0053 moles) were introduced into the aforementioned combination and subjected to vigorous agitation for a duration of 24 to 30 hours at a temperature of 25°C. A solution containing 25% aqueous sodium chloride (NaCl) was employed to terminate the reaction, followed by the extraction of the resulting combination using ethyl acetate in three separate aliquots of 15 ml each. The ethyl acetate extract that was collected underwent a washing process using a saturated solution of Na_2_CO_3_ (20 ml) and 1N HCl (15 ml). Subsequently, a 10 ml solution of sodium chloride (NaCl) with a concentration of 25% was introduced into the procedure. After the ethyl acetate extract was dried using anhydrous sodium sulphate, it was concentrated using a rota-vapour. The resultant residue underwent purification by column chromatography, employing an eluent mixture of ethyl acetate and petroleum ether in a ratio of 6:4. The supplementary file contains all relevant spectra.

#### 3.3.1. Synthesis of (3a): N’-(2-phenoxyacetyl)-4-(1H-pyrrol-1-yl) benzohydrazide

White amorphous solid. (Yield 65%). M.p 138–140°C; FTIR (KBr-cm^-^): 3425 (NH), 3239 (NH), 2919 (Ar-C = CH), 1697 (C = O), 1652 (C = O).

^1^H NMR (8 mg-DMSO-d_6_, 400 MHz, T-18.85°C, *δ* ppm): 10.30 (1H, s, CONH), 10.26 (1H, s, NHCO), 8.00 (2H, d, *J* = 8.76 Hz, bridging phenyl-C_8_, C_10_-H), 7.78 (2H, d, *J* = 8.56 Hz, bridging phenyl-C_7_, C_11_-H), 7.51 (2H, d, *J* = 2.24 Hz, pyrrole-C_2_, C_5_-H), 7.35–7.30 (2H, m, phenyl-C_22_, C_24_-H), 7.05–6.90 (3H, m, phenyl-C_21_, C_23,_ C_25_-H), 6.32 (2H, d, *J* = 2.12 Hz, pyrrole-C_3_, C_4_-H), 4.69 (2H, d, *J* = 6.04 Hz, CH_2_-H).

^13^C NMR (10 mg-DMSO-d_6_, 400 MHz, T-18.85°C, *δ* ppm): 167.44 (-NHCO), 164.61 (-CONH), 156.58 (phenyl-C_20_), 143.60 (bridging phenyl-C_6_), 129.50 (bridging phenyl-C_8_, C_10_^)^, 129.37 (bridging phenyl-C_7_, C_11_^)^, 129.17 (phenyl-C_22_, C_24_), 127.67 (bridging phenyl-C_9_), 121.33 (phenyl-C_23_), 116.86 (pyrrole-C_2_, C_5_^)^, 115.72 (phenyl-C_21_, C_25_), 106. 18 (pyrrole-C_3_, C_4_^)^, 66.58 (-CH_2_).

Mass (ESI- *m/z*) = found 335.2440 [M^+^]; Calcd. 335.36.

CHN Anal. For C_19_H_17_N_3_O_3_: Calcd. C, 68.05; H, 5.11; N, 12.53; Found: C, 68.01; H, 5.04; N, 12.49.

#### 3.3.2. Synthesis of (3b): 4-(1H-pyrrol-1-yl)-N’-(2-(p-tolyloxy)acetyl)benzohydrazide

White amorphous solid. (Yield 59%). M.p 162–165°C; FTIR (KBr-cm^-^): 3381 (NH), 3239 (NH), 2922 (Ar-C = CH), 1691(C = O), 1650 (C = O).

^1^H NMR (8 mg-DMSO-d_6_, 400 MHz, T-18.85°C, *δ* ppm): 10.21 (1H, s, CONH), 10.10 (1H, s, NHCO), 8.03 (2H, d, *J* = 8.32 Hz, bridging phenyl-C_8_, C_10_-H), 7.64 (2H, d, *J* = 8.28 Hz, bridging phenyl-C_7_, C_11_-H), 7.36 (2H, d, *J* = 7.04 Hz, pyrrole-C_2_, C_5_-H), 7.08 (2H, d, *J* = 10.44 Hz, phenyl-C_22_, C_24_-H), 6.92 (2H, d, *J* = 8.04 Hz, phenyl-C_21_ C_25_-H), 6.30 (2H, s, pyrrole-C_3_, C_4_-H), 4.61 (2H, s, CH_2_-H), 2.24 (3H, s, CH_3_-H).

^13^C NMR (10 mg-DMSO-d_6_, 400 MHz, T-18.85°C, *δ* ppm): 167.29, (-NHCO), 164.89 (-CONH), 155.63 (phenyl-C_20_), 141.30 (bridging phenyl-C_6_), 129.99 (bridging phenyl-C_8_, C_10_), 128.49 (bridging phenyl-C_7_, C_11_), 127.91 (phenyl-C_22_, C_24_), 127.51 (bridging phenyl-C_9_), 121.35 (phenyl-C_23_), 116.71 (pyrrole-C_2_, C_5_), 114.58 (phenyl-C_21_, C_25_), 106.47 (pyrrole-C_3_, C_4_), 66.25 (-CH_2_), 20.05 (-CH_3_).

Mass (ESI- *m/z*) = Found 349.7444 [M^+^]; Calcd. 349.39.

CHN Anal. For C_20_H_19_N_3_O_3_: Calcd. C, 68.75; H, 5.48; N, 12.03; Found: C, 68.69; H, 5.41; N, 12.01.

#### 3.3.3. Synthesis of (3c): N’-(2-(2-hydroxyphenoxy)acetyl)-4-(1H-pyrrol-1-yl)benzohydrazide

White amorphous solid. (Yield 75%). M.p 182–185°C; FTIR (KBr-cm^-^): 3402 (OH), 3398 (NH), 3279 (NH), 2924 (Ar-C = CH), 1681 (C = O), 1643 (C = O).

^1^H NMR (8 mg-DMSO-d_6_, 400 MHz, T-18.85°C, *δ* ppm): 10.53 (1H, s, CONH), 10.45 (1H, s, NHCO), 9.00 (1H, s, OH), 8.03 (2H, d, *J* = 8.76 Hz, bridging phenyl-C_8_, C_10_-H), 7.77 (2H, d, *J* = 8.80 Hz, bridging phenyl-C_7_, C_11_-H), 7.52 (2H, d, *J* = 2.24 Hz, pyrrole-C_2_, C_5_-H), 7.04 (1H, d, *J* = 7.56 Hz, phenyl-C_22_), 6.86–6.77 (3H, m, phenyl-C_23,_ C_24_, C_25_-H), 6.30 (2H, d, *J* = 2.16 Hz, pyrrole-C_3_, C_4_-H), 4.68 (2H, s, CH_2_-H).

^13^C NMR (10 mg-DMSO-d_6_, 400 MHz, T-18.85°C, *δ* ppm): 167.00 (-NHCO), 164.86 (-CONH), 146.14 (phenyl-C_21_), 145.29 (phenyl-C_20_), 142.34 (bridging phenyl-C_6_), 129.15 (bridging phenyl-C_8_, C_10_), 128.49 (bridging phenyl-C_7_, C_11_), 122.15 (phenyl-C_22_, C_24_), 119.32 (bridging phenyl-C_9_), 118.97 (phenyl-C_23_), 116.05 (pyrrole-C_2_, C_5_), 113.47 (phenyl-C_25_), 111.23 (pyrrole-C_3_, C_4_), 67.11 (-CH_2_).

Mass (ESI- *m/z*) = Found 352.1259 [M^+^ + H]; Calcd. 351.36).

CHN Anal. For C_19_H_17_N_3_O_4_: Calcd. C, 64.95; H, 4.88; N, 11.96; Found: C, 64.90; H, 4.79; N, 11.87.

#### 3.3.4. Synthesis of (3d): N’-(2-(2,4-dimethylphenoxy)acetyl)-4-(1H-pyrrol-1yl)benzohydrazide

White amorphous solid. (Yield 59%). M.p 190–192°C; FTIR (KBr-cm^-^): 3391 (NH), 3207 (NH), 2922 (Ar-C = CH), 1694 (C = O), 1613 (C = O).

^1^H NMR (8 mg-DMSO-d_6_, 400 MHz, T-18.85°C, *δ* ppm): 10.49 (1H, s, CONH), 10.14 (1H, s, NHCO), 8.02 (2H, d, *J* = 8.72 Hz, bridging phenyl-C_8_, C_10_-H), 7.76 (2H, d, *J* = 8.76 Hz, bridging phenyl-C_7_, C_11_-H), 7.51 (2H, d, *J* = 2.2 Hz, pyrrole-C_2_, C_5_-H), 7.04 (2H, d, *J* = 9.08 Hz, phenyl-C_22_, C_24_-H), 6.86 (1H, d, *J* = 2.2 Hz, phenyl-C_25-_H), 6.33–6.32 (2H, t, *J* = 2.16 Hz, *J* = 2.12 Hz, pyrrole-C_3_, C_4_-H), 4.65 (2H, s, CH_2_-H), 2.22 (6H, s, di-CH_3-_H).

^13^C NMR (10 mg-DMSO-d_6_, 400 MHz, T-18.85°C, *δ* ppm): 167.44 (-NHCO), 164.61 (-CONH), 153.89 (phenyl-C_20_), 142.27 (bridging phenyl-C_6_), 131.24 (phenyl-C_23_), 129.63 (bridging phenyl-C_8_, C_10_), 129.14 (bridging phenyl-C_7_, C_11_), 128.54 (phenyl-C_22_, C_24_), 126.95 (phenyl-C_21_), 125.96 (bridging phenyl-C_9_), 118.96 (pyrrole-C_2_, C_5_), 118.42 (phenyl-C_25_), 111.69 (pyrrole-C_3_, C_4_), 66.58 (CH_2_), 20,04 (phenyl-CH_3_), 16.05 (phenyl-CH_3_).

Mass (ESI- *m/z*) = Found 365.2626 [M^+^ + 2]; Calcd. 363.42.

CHN Anal. For C_21_H_21_N_3_O_3_: Calcd. C, 69.41; H, 5.82; N, 11.56; Found: C, 69.39; H, 5.76; N, 11.54.

#### 3.3.5. Synthesis of (3e): 4-(1H-pyrrol-1-yl)-N’-(2-(m-tolyloxy)acetyl)benzohydrazide

White amorphous solid. (Yield 77%). M.p 176–178°C; FTIR (KBr-cm^-^): 3357 (NH), 3236 (NH), 2922 (Ar-C = CH), 1694 (C = O), 1650 (C = O).

^1^H NMR (8 mg-DMSO-d_6_, 400 MHz, T-18.85°C, *δ* ppm): 10.47 (1H, s, CONH), 10.25 (1H, s, NHCO), 8.01 (2H, d, *J* = 8.60 Hz, bridging phenyl-C_8_, C_10_-H), 7.78 (2H, d, *J* = 8.44 Hz, bridging phenyl-C_7_, C_11_-H), 7.52 (1H, d, *J* = 1.72 Hz, phenyl-C_25-_H), 7.20 (2H, d, *J* = 2.48 Hz, pyrrole-C_2_, C_5_-H), 6.88–6.74 (3H, m, phenyl-C_21_, C_22_, C_23_-H), 6.33 (2H, d, *J* = 1.72 Hz, pyrrole-C_3_, C_4_-H), 4.75 (2H, s, CH_2_-H), 2.28 (3H, s, CH_3-_H).

^13^C NMR (10 mg-DMSO-d_6_, 400 MHz, T-18.85°C, *δ* ppm): 167.26 (-NHCO), 164.67 (-CONH), 157.74 (phenyl-C_20_), 141.91 (bridging phenyl-C_6_), 129.61 (bridging phenyl-C_8_, C_10_), 128.55 (bridging phenyl-C_7_, C_11_), 123.03 (phenyl-C_22_, C_24_), 121.96 (bridging phenyl-C_9_), 121.09 (C-23), 115.36 (pyrrole-C_2_, C_5_), 111.77 (phenyl-C_21,_ C_25_), 110.57 (pyrrole-C_3_, C_4_), 66.04 (CH_2_), 21.09 (phenyl-CH_3_).

Mass (ESI- *m/z*) = Found 349.2064 [M^+^]; Calcd. 349.39.

CHN Anal. For C_20_H_19_N_3_O_3_: Calcd. C, 68.75; H, 5.48; N, 12.03; Found: C, 68.67; H, 5.46; N, 12.04.

#### 3.3.6. Synthesis of (3f): N’-(2-(4-chlorophenoxy)acetyl)-4-(1H-pyrrol-1-yl)benzohydrazide

White amorphous solid. (Yield 68%). M.p 161–163°C; FTIR (KBr-cm^-^): 3373 (NH), 3257 (NH), 2915 (Ar-C = CH), 1723 (C = O), 1649 (C = O).

^1^H NMR (8 mg-DMSO-d_6_, 400 MHz, T-18.85°C, *δ* ppm): 10.43 (1H, s, CONH), 10.26 (1H, s, NHCO), 8.00 (2H, d, *J* = 8.00 Hz, bridging phenyl-C_8_, C_10_-H), 7.75 (2H, d, *J* = 5.12 Hz, bridging phenyl-C_7_, C_11_-H), 7.48 (2H, d, *J* = 4.28 Hz, phenyl-C_22_, C_24_-H), 7.29 (2H, d, *J* = 5.16 Hz, pyrrole-C_2_, C_5_-H), 7.00–6.96 (3H, m, phenyl-C_21_, C_21_, C_25_-H) 6.34 (2H, s, pyrrole-C_3_, C_4_-H), 4.65 (2H, s, CH_2_-H).

^13^C NMR (10 mg-DMSO-d_6_, 400 MHz, T-18.85°C, *δ* ppm): 166.90 (-NHCO), 164.58 (-CONH), 156.58 (phenyl-C_20_), 143.89 (bridging phenyl-C_6_), 130.90 (phenyl-C_22,_ C_24_), 129.20 (bridging phenyl-C_8_, C_10_), 128.19 (phenyl-C_21,_ C_25_), 124.98 (bridging phenyl-C_7_, C_11_), 122.97 (bridging phenyl-C_9_), 121.03 (phenyl-C_22_), 116.57 (pyrrole-C_2_, C_5_), 110.25 (pyrrole-C_3_, C_4_), 66.35 (CH_2_).

Mass (ESI- *m/z*) = Found 371.0744 [M^+^ +2]; Calcd. 369.81.

CHN Anal. For C_19_H_16_ClN_3_O_3_: Calcd. C, 61.71; H, 4.36; N, 11.36; Found: C, 61.69; H, 4.32; N, 11.30.

#### 3.3.7. Synthesis of (3g): N’-(2-(3,5-dihydroxyphenoxy)acetyl)-4-(1H-pyrrol-1- yl) benzohydrazide

White amorphous solid. (Yield 69%). M.p 160–162°C; FTIR (KBr-cm^-^): 3367 (NH), 3220 (NH), 2923 (Ar-C = CH), 1702 C = O), 1621 C = O).

^1^H NMR (8 mg-DMSO-d_6_, 400 MHz, T-19.95°C, *δ* ppm): 10.46 (1H, s, CONH), 10.22 (1H, s, NHCO), 9.46 (1H, s, OH), 8.99 (1H, s, OH), 7.96 (1H, s, phenyl-C_21_-H), 7.90 (1H, s, phenyl-C_25_-H), 7.75 (2H, d, *J* = 4.56 Hz, bridging phenyl-C_8_, C_10_-H), 7.68 (2H, d, *J* = 7.76 Hz, bridging phenyl-C_7_, C_11_-H), 7.47 (2H, d, *J* = 2.2 Hz, pyrrole-C_2_, C_5_-H), 6.31 (2H, d, *J* = 2.16 Hz, pyrrole-C_3_, C_4_-H), 5.68 (1H, s, phenyl-C_23_-H), 4.51 (2H, s, CH_2_-H).

^13^C NMR (10 mg-DMSO-d_6_, 400 MHz, T-19.95°C, *δ* ppm): 167.09 (-NHCO), 164.45 (-CONH), 158.87 (phenyl-C_20_), 157.64 (phenyl-C_22,_ C_24_), 141.83 (bridging phenyl-C_6_), 129.35 (bridging phenyl-C_8_, C_10_), 129.18 (CH, C-7, C-11), 126.64 (bridging phenyl-C_9_), 118.93 (bridging phenyl-C_7_, C_11_), 111.14 (pyrrole-C_3_, C_4_), 110.22 (phenyl-C_23_), 94.01 (phenyl-C_21,_ C_25_), 66.35 (CH_2_).

Mass (ESI- *m/z*) = found 368.0277 [M^+^ + H]; Calcd. 367.36.

CHN Anal. For C_19_H_17_N_3_O_5_: Calcd. C, 62.12; H, 4.66; N, 11.44; Found: C, 62.09; H, 4.61; N, 11.42.

#### 3.3.8. Synthesis of (3h):N’-(2-(3,5-dimethylphenoxy)acetyl)-4-(1H-pyrrol-1-yl) benzohydrazide

White amorphous solid. (Yield 72%). M.p 166–168°C; FTIR (KBr-cm^-^): 3400 (NH), 3264 (NH), 2918 (Ar-C = CH), 1720 (C = O), 1600 C = O).

^1^H NMR (8 mg-DMSO-d_6_, 400 MHz, T-19.15°C, *δ* ppm): 10.50 (1H, s, CONH), 10.26 (1H, s, NHCO), 8.03 (2H, d, *J* = 7.04 Hz, bridging phenyl-C_8_, C_10_-H), 7.76 (2H, d, *J* = 8.84 Hz, bridging phenyl-C_7_, C_11_-H), 7.51 (2H, d, *J* = 2.28 Hz, pyrrole-C_2_, C_5_-H), 6.67 (2H, s, phenyl-C_21_, C_25_-H), 6.62 (1H, s, phenyl-C_23_-H), 6.33 (2H, d, *J* = 2.24 Hz, pyrrole-C_3_, C_4_-H), 4.64 (2H, s, CH_2_-H), 2.23 (6H, s, di-CH_3_-H).

^13^C NMR (10 mg-DMSO-d_6_, 400 MHz, T-19.15°C, *δ* ppm): 167.37 (-NHCO), 164.71 (-CONH), 157.74 (phenyl-C_20_), 142.27 (phenyl-C_22_, C_24_), 138.62 (bridging phenyl-C_6_), 129.16 (bridging phenyl-C_8_, C_10_), 128.53 (bridging phenyl-C_7_, C_11_), 122.82 (bridging phenyl-C_9_), 118.94 (pyrrole-C_2_, C_5_), 119.41 (pyrrole-C_3_, C_4_), 112.45 (phenyl-C_23_), 111.21(phenyl-C_21_, C_25_), 65.99(CH_2_), 21.03 (di-CH_3_).

Mass (ESI- *m/z*) = Found 363.1623 [M^+^]; Calcd. 363.42.

CHN Anal. For C_21_H_21_N_3_O_3_: Calcd. C, 69.41; H, 5.82; N, 11.56; Found: C, 69.40; H, 5.79; N, 11.52.

#### 3.3.9. Synthesis of (3i): N’-(2-(3-hydroxyphenoxy)acetyl)-4-(1H-pyrrol-1-yl)benzohydrazide

White amorphous solid. (Yield 67%). M.p 173–175°C; FTIR (KBr-cm^-^): 3387 (NH), 3277 (NH), 2924 (Ar-C = CH), 1723 (C = O), 1603 (C = O).

^1^H NMR (8 mg-DMSO-d_6_, 400 MHz, T-19.15°C, *δ* ppm): 10.52 (1H, s, CONH), 10.26 (1H, s, NHCO), 9.01 (1H, s, OH-H), 8.03 (2H, d, *J* = 7.14 Hz, bridging phenyl-C_8_, C_10_-H), 7.76 (2H, d, *J* = 8.24 Hz, bridging phenyl-C_7_, C_11_-H), 7.51 (2H, d, *J* = 2.24 Hz, pyrrole-C_2_, C_5_-H), 6.66–6.51 (4H, m, phenyl-C_21_,C_23_, C_24_, C_25_-H), 6.33 (2H, d, *J* = 2.24, pyrrole-C_3_, C_4_-H), 4.85 (2H, s, CH_2_-H).

^13^C NMR (10 mg-DMSO-d_6_, 400 MHz, T-19.15°C, *δ* ppm): 167.25 (-NHCO), 164.90 (-CONH), 157.74 (phenyl-C_20_), 141.31(bridging phenyl-C_6_), 131.35 (phenyl-C_24_), 130.11 (phenyl-C_22_), 129.17 (bridging phenyl-C_7_, C_11_), 128.83 (bridging phenyl-C_8_, C_10_), 128.15 (bridging phenyl-C_9_), 121.97 (pyrrole-C_2_, C_5_), 111.78 (pyrrole-C_3_, C_4_), 108.32 (phenyl-C_23_), 106.66 (phenyl-C_25_), 102.12 (phenyl-C_21_), 66.3 (CH_2_).

Mass (ESI- *m/z*) = Found 352.2195 [M^+^ + H]; Calcd. 351.36.

CHN Anal. For C_19_H_17_N_3_O_4_: Calcd. C, 64.95; H, 4.88; N, 11.96; Found: C, 64.91; H, 4.81; N, 11.89.

#### 3.3.10. Synthesis of (3j): N’-(2-(2-chlorophenoxy)acetyl)-4-(1H-pyrrol-1-yl)benzohydrazide

White amorphous solid. (Yield 65%). M.p 156–158°C; FTIR (KBr-cm^-^): 3355 (NH), 3269 (NH), 2917 (Ar-C = CH), 1714 (C = O), 1647 (C = O).

^1^H NMR (8 mg-DMSO-d_6_, 400 MHz, T-18.85°C, *δ* ppm): 10.55 (1H, s, CONH), 10.28 (1H, s, NHCO), 7.99 (2H, d, *J* = 4.28 Hz, bridging phenyl-C_8_, C_10_-H), 7.76 (2H, d, *J* = 5.64 Hz, bridging phenyl-C_7_, C_11_-H), 7.49 (2H, d, *J* = 5.16 Hz, pyrrole-C_2_, C_5_-H), 7.45–6.97 (4H, m, phenyl-C_22_,C_23_, C_24_, C_25_-H), 6.32 (2H, d, *J* = 2.16 Hz, pyrrole-C_3_, C_4_-H), 4.83 (2H, s, CH_2_-H).

^13^C NMR (10 mg-DMSO-d_6_, 400 MHz, T-18.85°C, *δ* ppm): 166.74 (-NHCO), 164.64 (-CONH), 153.37 (phenyl-C_20_), 142.30 (bridging phenyl-C_6_), 130.04 (phenyl-C_22,_ C_24_), 129.16 (C-8, bridging phenyl-C_8_, C_10_), 128.41 (phenyl-C_21_), 128.19 (bridging phenyl-C_7_, C_11_), 122.22 (bridging phenyl-C_9_), 121.49 (phenyl-C_23_), 118.95 (pyrrole-C_2_, C_5_), 114.22 (phenyl-C_23_), 111.21 (pyrrole-C_3_, C_4_), 66.59 (CH_2_).

Mass (ESI- *m/z*) = Found 371.5759 [M^+^ +2]; Calcd. 369.81.

CHN Anal. For C_19_H_16_ClN_3_O_3_: Calcd. C, 61.71; H, 4.36; N, 11.36; Found: C, 61.70; H, 4.32; N, 11.32.

#### 3.3.11. Synthesis of (5a): 4-(2,5-dimethyl-1H-pyrrol-1-yl)-N’-(2-phenoxyacetyl) benzohydrazide

Yellow crystalline solid. (Yield 70%). M.p 140–142°C. FTIR (KBr-cm^-^): 3411 (NH), 3227 (NH), 2923 (Ar-C = CH), 1696 (C = O), 1647 (C = O).

^1^H NMR (8 mg-DMSO-d_6_, 400 MHz, T-19.75°C, *δ* ppm): 10.58 (1H, s, CONH), 10.35 (1H, s, NHCO), 8.05 (2H, d, *J* = 8.48 Hz, bridging phenyl-C_8_, C_10_-H), 7.43 (2H, d, *J* = 8.44 Hz, bridging phenyl-C_7_, C_11_-H), 7.36–7.29 (2H, m, phenyl-C_24_, C_26_-H), 7.06–6.93 (3H, m, phenyl-C_23_, C_25_, C_27_-H), 5.86 (2H, d, *J* = 4.88 Hz, pyrrole-C_3_, C_4_-H), 4.69 (2H, s, CH_2_-H), 2.01 (6H, s, diCH_3_-H).

^13^C NMR (10 mg-DMSO-d_6_, 400 MHz, T-19.75°C, *δ* ppm): 167.22 (-NHCO), 164.93 (-CONH), 157.67 (phenyl-C_22_), 141.32 (bridging phenyl-C_6_), 131.30 (phenyl-C_24,_ C_26_), 129.47 (bridging phenyl-C_7_, C_11_), 128.51 (bridging phenyl-C_8_, C_10_), 127.96 (bridging phenyl-C_9_), 121.26 (pyrrole-C_2_, C_5_), 114.71 (phenyl-C_25_), 114.40 (CH, phenyl-C_23,_ C_27_), 106.48 (CH, pyrrole-C_3_, C_4_), 66.05 (CH_2_), 12.85 (pyrrole-diCH_3_).

Mass (ESI- *m/z*) = Found 363.1381 [M^+^]; Calcd. 363.42.

CHN Anal. For C_21_H_21_N_3_O_3_: Calcd. C, 69.41; H, 5.82; N, 11.56; Found: C, 69.40; H, 5.78; N, 11.55.

#### 3.3.12. Synthesis of (5b):4-(2,5-dimethyl-1H-pyrrol-1-yl)-N’-(2-(p-tolyloxy)acetyl) benzohydrazide

Yellow crystalline solid. (Yield 65%). M.p 164–166°C. FTIR (KBr-cm^-^): 3391 (NH), 3190 (NH), 2920 (Ar-C = CH), 1698 (C = O), 1653 (C = O).

^1^H NMR (8 mg-DMSO-d_6_, 400 MHz, T-22.15°C, *δ* ppm): 10.54 (1H, s, CONH), 10.29 (1H, s, NHCO), 8.03 (2H, d, *J* = 6.72 Hz, bridging phenyl-C_8_, C_10_-H), 7.43 (2H, d, *J* = 8.44 Hz, bridging phenyl-C_7_, C_11_-H), 7.14 (2H, d, *J* = 8.32 Hz, phenyl-C_24_, C_26_-H), 6.94 (2H, d, *J* = 8.56 Hz, phenyl-C_23_, C_27_-H), 5.84 (2H, s, pyrrole-C_3_, C_4_-H), 4.63 (2H, s, CH_2_-H), 2.24 (3H, s, phenyl-CH_3_-H), 2.03 (6H, s, pyrrole-diCH_3_-H).

^13^C NMR (10 mg-DMSO-d_6_, 400 MHz, T-22.15°C, *δ* ppm): 167.29 (-NHCO), 164.89 (-CONH), 155.63 (phenyl-C_22_), 141.30 (bridging phenyl-C_6_), 131.32 (phenyl-C_24,_ C_26_), 129.99 (bridging phenyl-C_7_, C_11_), 129.78 (bridging phenyl-C_8_, C_10_), 128.49 (bridging phenyl-C_9_), 127.51 (pyrrole-C_2_, C_5_), 114.58 (phenyl-C_25_), 112.14 (phenyl-C_23_, C_27_), 106.47 (pyrrole-C_3_, C_4_), 66.2 (CH_2_), 12.85 (phenyl-C_25_), 20.05 (phenyl-diCH_3_), 12.85 (pyrrole-diCH_3_).

Mass (ESI- *m/z*) = Found 377.1119 [M^+^]; Calcd. 377.44.

CHN Anal. For C_22_H_23_N_3_O_3_: Calcd. C, 70.01; H, 6.14; N, 11.13; Found: C, 70.01; H, 6.14; N, 11.13.

#### 3.3.13. Synthesis of (5c) 4-(2,5-dimethyl-1H-pyrrol-1-yl)-N’-(2-(2-hydroxyphenoxy) acetyl) benzohydrazide

Yellow crystalline solid. (Yield 60%). M.p 180–182°C. FTIR (KBr-cm^-^): 3403 (OH), 3312 (NH), 3273 (NH), 2922 (Ar-C = CH), 1651 (C = O), (C = O).

^1^H NMR (8 mg-DMSO-d_6_, 400 MHz, T-21.85°C, *δ* ppm): 10.61 (1H, s, CONH), 10.33 (1H, s, NHCO), 8.99 (1H, s, OH), 8.03 (2H, d, *J* = 8.76 Hz, bridging phenyl-C_8_, C_10_-H), 7.52 (2H, d, *J* = 6.24 Hz, bridging phenyl-C_7_, C_11_-H), 7.04–6.85 (4H, m, phenyl-C_24_, C_25,_ C_26_, C_27_-H), 6.31 (2H, s, pyrrole-C_3_, C_4_), 4.78 (2H, s, CH_2_-H), 1.84 (6H, s, pyrrole-diCH_3_-H).

^13^C NMR (10 mg-DMSO-d_6_, 400 MHz, T-21.85°C, *δ* ppm): 167.06 (-NHCO), 165.13 (-CONH), 146.10 (phenyl-C_22_), 145.26 (bridging phenyl-C_6_), 128.52 (phenyl-C_24_), 128.01 (bridging phenyl-C_7_, C_11_), 127.53 (CH, bridging phenyl-C_8_, C_10_), 122.19 (bridging phenyl-C_9_), 119.36 (pyrrole-C_2_, C_5_), 116.08 (phenyl-C_25_, C_26_), 113.43 (phenyl-C_23_, C_27_), 106.50 (pyrrole-C_3_, C_4_), 67.06 (CH_2_), 12.82 (pyrrole-diCH_3_).

Mass (ESI- *m/z*) = Found 380.1251 [M^+^ + H]; Calcd. 379.42.

CHN Anal. For C_21_H_21_N_3_O_4_: Calcd. C, 66.48; H, 5.58; N, 11.08; Found: C, 66.48; H, 5.58; N, 11.08.

#### 3.3.14. Synthesis of (5d): 4-(2,5-dimethyl-1H-pyrrol-1-yl)-N’-(2-(2,4-dimethylphenoxy) acetyl) benzohydrazide

Yellow crystalline solid. (Yield 55%). M.p 186–188°C. FTIR (KBr-cm^-^): 3350 (NH), 3226 (NH), 2921 (Ar-C = CH), 1700 (C = O), 1653 (C = O).

^1^H NMR (8 mg-DMSO-d_6_, 400 MHz, T-22.15°C, *δ* ppm): 10.57 (1H, s, CONH), 10.20 (1H, s, NHCO), 8.04 (2H, d, *J* = 8.46 Hz, bridging phenyl-C_8_, C_10_-H), 7.42 (2H, d, *J* = 8.52 Hz, bridging phenyl-C_7_, C_11_-H), 6.98–6.84 (3H, m, phenyl-C_24_, C_26_, C_27_-H), 5.84 (2H, s, pyrrole-C_3_, C_4_), 4.65 (2H, s, phenyl-C_20_-H), 2.22 (3H, s, phenyl-C_29_-CH_3-_H), 2.18 (3H, s, phenyl- C_28-_CH_3-H_), 1.99 (6H, s, pyrrole-diCH_3_-H).

^13^C NMR (10 mg-DMSO-d_6_, 400 MHz, T-22.15°C, *δ* ppm): 167.46 (-NHCO), 164.84 (CONH), 153.89 (phenyl-C_24_), 141.30 (bridging phenyl-C_6_), 131.32 (phenyl-C_26_), 129.66 (bridging phenyl-C_7_, C_11_), 128.50 (bridging phenyl-C_8_, C_10_), 127.95 (bridging phenyl-C_9_), 126.95 (phenyl-C_22_), 125.97 (pyrrole-C_2_, C_5_), 114.71 (phenyl-C_25_), 111.71 (phenyl-C_23,_ C_27_), 106.47 (pyrrole-C_3_, C_4_), 66.58 (CH_2_), 20.03 (phenyl-C_28_ -CH_3_), 16.05 (phenyl-C_29_ -CH_3-_H), 12.85 (pyrrole-diCH_3-_H).

Mass (ESI- *m/z*) = Found 391.2626 [M^+^]; Calcd. 391.19.

CHN Anal. For C_23_H_25_N_3_O_3_: Calcd. C, 70.57; H, 6.44; N, 10.73; Found: C, 70.57; H, 6.44; N, 10.73.

#### 3.3.15. Synthesis of (5e): 4-(2,5-dimethyl-1H-pyrrol-1-yl)-N’-(2-(m-tolyloxy) acetyl) benzohy drazide

Yellow crystalline solid. (Yield 67%). M.p 170–172°C; FTIR (KBr-cm^-^): 3378 (NH), 3223 (NH), 2921 (Ar-C = CH), 1697 (C = O), 1649 (C = O).

^1^H NMR (8 mg-DMSO-d_6_, 400 MHz, T-22.45°C, *δ* ppm): 10.57 (1H, s, CONH), 10.32 (1H, s, NHCO), 8.11 (2H, d, *J* = 8.48 Hz, bridging phenyl-C_8_, C_10_-H), 7.45 (2H, d, *J* = 8.52 Hz, bridging phenyl-C_7_, C_11_-H), 7.22–6.74 (4H, m, phenyl-C_24_, C_25_, C_26_, C_27_-H), 5.84 (2H, s, pyrrole-C_3_, C_4_), 4.67 (2H, s, CH_2_-H), 2.31 (3H, s, phenyl- C_28-_CH_3-_H), 2.02 (6H, s, pyrrole-diCH_3-_H).

^13^C NMR (10 mg-DMSO-d_6_, 400 MHz, T-22.45°C, *δ* ppm): 167.25 (NHCO), 164.90 (CONH), 157.74 (phenyl-C_22_), 141.31 (bridging phenyl-C_6_), 138.98 (phenyl-C_24_, C-_26_), 131.35 (bridging phenyl-C_7_, C_11_), 130.11 (bridging phenyl-C_8_, C_10_), 129.17 (bridging phenyl-C_9_), 121.97 (pyrrole-C_2_, C_5_), 115.36 (phenyl-C_25_), 111.78 (phenyl-C_23,_ C_27_), 106.66 (pyrrole-C_3_, C_4_), 66.05 (CH_2_), 21.07 (phenyl-C_28_ -CH_3_), 12.85 (pyrrole-diCH_3-_H).

Mass (ESI- *m/z*) = Found 377.4952 [M^+^]; Calcd. 377.44.

CHN Anal. For C_22_H_23_N_3_O_3_: Calcd. C, 70.01; H, 6.14; N, 11.13; Found: C, 70.01; H, 6.14; N, 11.13.

#### 3.3.16. Synthesis of (5f): N’-(2-(4-chlorophenoxy)acetyl)-4-(2,5-dimethyl-1H-pyrrol-1-yl) ben zohydrazide

Yellow crystalline solid. (Yield 78%). M.p 156–158°C. FTIR (KBr-cm^-^): 3385 (NH), 3248 (NH), 2922 (Ar-C = CH), 1681 (C = O), 1647 (C = O).

^1^H NMR (8 mg-DMSO-d_6_, 400 MHz, T-25.51°C, *δ* ppm): 10.55 (1H, s, CONH), 10.33 (1H, s, NHCO), 8.03 (2H, d, *J* = 8.48 Hz, bridging phenyl-C_8_, C_10_-H), 7.43 (2H, d, *J* = 8.92 Hz, bridging phenyl-C_7_, C_11_-H), 7.40 (2H, d, *J* = 5.52 Hz, phenyl-C_24_, C_26_-H), 7.08 (2H, d, *J* = 6.76 Hz, phenyl-C_23_, C_27_-H), 5.84 (2H, s, pyrrole-C_3_, C_4_), 4.70 (2H, s, CH_2_-H), 2.00 (6H, s, pyrrole-diCH_3-_H).

^13^C NMR (10 mg-DMSO-d_6_, 400 MHz, T-25.51°C, *δ* ppm): 166.90 (NHCO), 164.92 (CONH), 157.57 (phenyl-C_22_), 141.32 (bridging phenyl-C_6_), 131.30 (phenyl-C_24_, C-_26_), 129.20 (bridging phenyl-C_7_, C_11_), 128.50 (bridging phenyl-C_8_, C_10_), 127.97 (bridging phenyl-C_9_), 127.51 (pyrrole-C_2_, C_5_), 125.01 (phenyl-C_25_), 116.52 (phenyl-C_23,_ C_27_), 106.48 (pyrrole-C_3_, C_4_), 66.35 (CH_2_), 12.84 (pyrrole-diCH_3-_H).

Mass (ESI- *m/z*) = Found 399.7788 [M^+^ +2]; Calcd. 397.86.

CHN Anal. For C_19_H_16_ClN_3_O_3_: Calcd. C, 63.40; H, 5.07; Cl, 8.91; N, 10.56; Found: C, 63.40; H, 5.07; Cl, 8.91; N, 10.56.

#### 3.3.17. Synthesis of (5g):N’-(2-(3,5-dihydroxyphenoxy)acetyl)-4-(2,5-dimethyl-1H-pyrrol-1-yl) benzohydrazide

Yellow crystalline solid. (Yield 88%). M.p 160–162°C; FTIR (KBr-cm^-^): 3355 (NH), 3224 (NH), 2923 (Ar-C = CH), 1711 (C = O), 1643 (C = O).

^1^H NMR (8 mg-DMSO-d_6_, 500 MHz, T-26.05°C, *δ* ppm): 10.30 (1H, s, CONH), 10.20 (1H, s, NHCO), 8.97 (2H, s, OH), 8.07 (2H, d, *J* = 8.80 Hz, bridging phenyl-C_8_, C_10_-H), 7.98 (2H, d, *J* = 7.66 Hz, bridging phenyl-C_7_, C_11_-H), 7.48 (2H, d, *J* = 8.5 Hz, phenyl-C_23_, C_27_-H), 7.01 (1H, s, phenyl-C_25_-H), 5.93 (2H, s, pyrrole-C_3_, C_4_), 4.34 (2H, s, CH_2_-H), 1.99 (6H, s, pyrrole-diCH_3-_H).

^13^C NMR (10 mg-DMSO-d_6_, 500 MHz, T-26.65°C, *δ* ppm): 166.21 (NHCO), 165.10 (CONH), 159.02 (phenyl-C_22_), 158.11 (bridging phenyl-C_6_), 140.40 (phenyl-C_24_), 130.59 (phenyl-C_26_), 129.26 (bridging phenyl-C_7_, C_11_), 128.61 (bridging phenyl-C_8_, C_10_), 127.99 (bridging phenyl-C_9_), 126.54 (pyrrole-C_2_, C_5_), 123.78 (phenyl-C_25_), 107.15 (phenyl-C_23,_ C_27_), 100.72 (pyrrole-C_3_, C_4_), 61.38 (CH_2_), 13.28 (pyrrole-diCH_3-_H).

Mass (ESI- *m/z*) = Found 396.2720 [M^+^ + H]; Calcd. 395.42.

CHN Anal. For C_21_H_21_N_3_O_5_: Calcd. C, 63.79; H, 5.35; N, 10.63; Found: C, 63.79; H, 5.35; N, 10.63.

#### 3.3.18. Synthesis of (5h): 4-(2,5-dimethyl-1H-pyrrol-1-yl)-N’-(2-(3,5-dimethylphenoxy) acetyl) benzohydrazide

Yellow solid. (Yield 76%). M.p 158–160°C. FTIR (KBr-cm^-^): 3318 (NH), 3235 (NH), 2920 (Ar-C = CH), 1649 (C = O), 1604 (C = O).

^1^H NMR (8 mg-DMSO-d_6_, 500 MHz, T-26.05°C, *δ* ppm): 10.55 (1H, s, CONH), 10.28 (1H, s, NHCO), 8.05 (2H, d, *J* = 8.50 Hz, bridging phenyl-C_8_, C_10_-H), 7.44 (2H, d, *J* = 7.55 Hz, bridging phenyl-C_7_, C_11_-H), 6.61 (2H, d, *J* = 8.5 Hz, phenyl-C_23_, C_27_-H), 6.58 (1H, s, phenyl-C_25_-H), 5.86 (2H, d, *J* = 4.80 Hz, pyrrole-C_3_, C_4_), 4.64 (2H, s, CH_2_-H), 2.26 (6H, s, phenyl-diCH_3-_H), 1.99 (6H, s, pyrrole-diCH_3-_H).

^13^C NMR (10 mg-DMSO-d_6_, 500 MHz, T-26.65°C, *δ* ppm): 167.80 (NHCO), 165.77 (CONH), 155.28 (phenyl-C_22_), 148.01 (bridging phenyl-C_6_), 141.81 (phenyl-C_24_), 130.61 (phenyl-C_26_), 129.61 (bridging phenyl-C_7_, C_11_), 128.66 (bridging phenyl-C_8_, C_10_), 127.11 (bridging phenyl-C_9_), 126.54 (pyrrole-C_2_, C_5_), 123.30 (phenyl-C_25_), 112.65 (phenyl-C_23,_ C_27_), 106.97 (pyrrole-C_3_, C_4_), 66.55 (CH_2_), 15.07 (phenyl-diCH_3-_H),14.61 (pyrrole-diCH_3-_H).

Mass (ESI- *m/z*) = Found 391.7207 [M^+^]; Calcd. 391.47.

CHN Anal. For C_23_H_25_N_3_O_3_: Calcd. C, 70.57; H, 6.44; N, 10.73; Found: C, 70.57; H, 6.44; N, 10.73.

#### 3.3.19. Synthesis of (5i): 4-(2,5-dimethyl-1H-pyrrol-1-yl)-N’-(2-(3-hydroxyphenoxy) acetyl) benzohydrazide

Yellow crystalline solid. (Yield 70%). M.p 124–126°C; FTIR (KBr-cm^-^): 3433 (OH), 3321 (NH), 3278 (NH), 2922 (Ar-C = CH), 1697 (C = O), 1652 (C = O).

^1^H NMR (8 mg-DMSO-d_6_, 500 MHz, T-26.05°C, *δ* ppm): 10.59 (1H, s, CONH), 10.29 (1H, s, NHCO), 9.50 (1H, s, OH), 8.07 (2H, d, *J* = 8.50 Hz, bridging phenyl-C_8_, C_10_-H), 7.42 (2H, d, *J* = 8.50 Hz, bridging phenyl-C_7_, C_11_-H), 7.11–6.92 (4H, m, phenyl-C_23_, C_25_, C_26_, C_27_-H), 5.87 (2H, d, *J* = 5.28 Hz, pyrrole-C_3_, C_4_), 4.62 (2H, s, CH_2_-H), 2.03 (6H, s, pyrrole-diCH_3-_H).

^13^C NMR (10 mg-DMSO-d_6_, 500 MHz, T-26.65°C, *δ* ppm): 167.68 (NHCO), 165.38 (CONH), 159.02 (phenyl-C_22_), 158.82 (phenyl-C_24_), 151.29 (bridging phenyl-C_6_), 141.83 (phenyl-C_26_), 130.61 (bridging phenyl-C_7_, C_11_), 128.68 (bridging phenyl-C_8_, C_10_), 113.68 (bridging phenyl-C_9_), 112.39 (pyrrole-C_2_, C_5_), 109.28 (phenyl-C_25_), 107.18 (phenyl-C_23_), 106.70 (phenyl-C_27_), 102.40 (pyrrole-C_3_, C_4_), 66.59 (CH_2_), 13.34 (pyrrole-diCH_3-_H).

Mass (ESI- *m/z*) = Found 381.2321 [M^+^+H]; Calcd. 379.42.

CHN Anal. For C_21_H_21_N_3_O_4_: Calcd. C, 66.48; H, 5.58; N, 11.08; Found: C, 66.48; H, 5.58; N, 11.08.

#### 3.3.20. Synthesis of (5j): N’-(2-(2-chlorophenoxy)acetyl)-4-(2,5-dimethyl-1H-pyrrol-1-yl) ben zohydrazide

Yellow crystalline solid. (Yield 55%). M.p 172–174°C. FTIR (KBr-cm^-^): 3390 (NH), 3243 (NH), 2928 (Ar-C = CH), 1679 (C = O), 1648 (C = O).

^1^H NMR (8 mg-DMSO-d_6_, 500 MHz, T-26.05°C, *δ* ppm): 10.41 (1H, s, CONH), 10.23 (1H, s, NHCO), 8.07 (2H, d, *J* = 8.28 Hz, bridging phenyl-C_8_, C_10_-H), 7.42 (2H, d, *J* = 8.22 Hz, bridging phenyl-C_7_, C_11_-H), 7.30–7.28 (2H, m, phenyl-C_24_, C_26_-H), 7.04–6.98 (2H, m, phenyl-C_25_, C_27_-H), 5.84 (2H, s, pyrrole-C_3_, C_4_), 4.66 (2H, s, CH_2_-H), 2.04 (6H, s, pyrrole-diCH_3-_H).

^13^C NMR (10 mg-DMSO-d_6_, 500 MHz, T-26.65°C, *δ* ppm): 166.41 (NHCO), 165.92 (CONH), 156.18 (phenyl-C_22_), 144.40 (phenyl-C_25_), 141.34 (bridging phenyl-C_6_), 132.16 (phenyl-C_24_, C_26_), 129.44 (bridging phenyl-C_7_, C_11_), 128.20 (bridging phenyl-C_8_, C_10_), 127.83 (bridging phenyl-C_9_), 122.36 (pyrrole-C_2_, C_5_), 118.71 (phenyl-C_23_), 116.26 (phenyl-C_27_), 106.48 (pyrrole-C_3_, C_4_), 66.65 (CH_2_), 12.95 (pyrrole-diCH_3-_H).

Mass (ESI- *m/z*) = Found 399.6951 [M^+^ +2]; Calcd. 397.86).

CHN Anal. For C_21_H_20_ClN_3_O_3_: Calcd. C, 63.40; H, 5.07; Cl, 8.91; N, 10.56; Found: C, 63.40;

H, 5.07; Cl, 8.91; N, 10.56;

### 3.4. Molecular docking using Surflex-Dock

The study employed the patented Sybyl-X 2.0 search tool and Surflex-Dock for molecular docking analysis. The aim of this study was to provide a comprehensive understanding of the molecular interactions between chemicals and the active sites of the ENR enzyme and DHFR enzyme [30]. This work provides a thorough analysis that can be applied to enhance the future optimization of molecular architectures. The crystallographic structures of enoyl acyl carrier protein reductase InhA, in complex with N-(4-methylbenzoyl)-4-benzylpiperidine (PDB ID 2NSD, resolution of 1.9 Å by X-ray diffraction), and dihydrofolate reductase of Mycobacterium tuberculosis, bound to NADPH and methotrexate, were obtained from the Brookhaven Protein Database (PDB) located at *http*:*//www*.*rcsb*.*org/pdb*. The ligands and protein employed in our docking methods were produced using the known Sybyl-X 2.0 standard protocol [31,32]. The inclusion of hydrogen atoms was necessary in order to establish the accurate configuration and tautomeric states. Subsequently, the structural model underwent energy minimization via the Tripos force field, incorporating a distance-dependent dielectric function. Partial atomic charges were then computed using the AM-BER7F9902 method. Lastly, the model was purged of water molecules. The molecular geometry of CP was later refined to achieve minimal energy by the utilization of the Powell energy minimization method. This process involved employing the Tripos force field together with Gasteiger-Hückel charges. Subsequently, the CP molecule was individually inserted into the binding pocket to facilitate the investigation of docking and scoring. In order to ascertain the interactions between the ligand and protein, the highest-ranking posture and protein were imported into the working environment. The MOLCAD application, which is a tool for molecular computer-aided design, was utilized to depict the manner in which the protein and ligand bind together.

### 3.5. ADMET studies

The toxicities were predicted using ProTox-II, and the corresponding results are shown in **Table 5**. Additionally, the Molecular ADME properties were estimated using the in silico Swiss ADME online tool [33–36], and the results are presented in **Table 4**.

### 3.6. MTT-based cytotoxicity activity

The cytotoxic activity (IC_50_) of selected compounds against A549 (lung adenocarcinoma) MV cell-lines was evaluated by performing cellular conversion of MTT [3-(4,5-dimethylthiazo-2-yl)-2,5-diphenyl-tetrazolium bromide] into a formazan product. This evaluation was conducted up to a concentration of 50 mg/mL using the Promega Cell Titer 96 non-radioactive cell proliferation assay, with cisplatin serving as the positive control. Cytotoxicity is commonly quantified by determining the IC50 value, which represents the quantity of a substance that reduces the optical density of treated cells by 50% compared to untreated cells, as measured by the MTT experiment. The IC_50_ values presented in Table 6 are the mean values ± standard error of the mean (SEM) obtained from three separate and independent measurements.

**Table 6:** Shows the *in vitro* cytotoxicity activity of selected drugs against human lung cancer (A549) cell lines, and MV cell lines (IC_50_ in μg/mL).

Compound	IC_50_ (μM)^a^	
	MV cell-lines ^b^	A549 ^c^
**3a**	-	-
**3b**	-	
**3c**	223±0.7	224±0.6
**3d**	216±0.4	212±0.4
**3e**	-	-
**3f**		
**3g**	216±0.5	216±0.5
**3h**	219±0.6	219±0.1
**3i**		
**3j**		
**5a**		
**5b**	-	-
**5c**	-	-
**5d**	226±0.5	222±0.5
**5e**	220±0.2	219±0.3
**5f**		
**5g**		
**5h**		
**5i**		
**5j**		
**INH**	>450	>450

^*a*^ *Values are the means ± SEM of three independent experiments*.

^*b*^ *Mammalian Vero cell-lines (NCCS-Pune*, *INDIA)*.

^*c*^ *A549 (lung adenocarcinoma) cell-lines (NCCS-Pune*, *INDIA)*.

### 3.6. Antitubercular activity

The efficacy of the newly synthesized compounds was assessed against the M. tuberculosis strain H37Rv using the Microplate Alamar Blue test (MABA). The obtained data, including the minimum inhibitory concentration (MIC) values, are presented in **Table 3** [37].

### 3.7. Antibacterial activity

The antibacterial inhibitory effects of all compounds were assessed using the broth microdilution method, with ciprofloxacin serving as the reference medication. The study focused on comparing the inhibitory effects against *S*. *aureus* (Gram-positive) and *E*. *coli* (Gram-negative) bacteria [38,39]. The antibacterial activity data, including the minimum inhibitory concentration (MIC) values, was summarized in Table 3.

## 4. Conclusion

The antitubercular and enzyme inhibitory effects of a set of 20 newly synthesized pyrrolyl-benzohydrazide derivatives were assessed. All the compounds had moderate to good potency against tuberculosis, as shown by MICs ranging from 1.6 to 12.5 μg/ml. The derivatives were subjected to molecular docking analysis, revealing that these newly identified inhibitors exhibited a close match inside the binding site of both the ENR-enzyme and DHFR enzyme, similar to the 2NSD_ligand and 1DF7_ligand. *In vitro* assays indicated that the compounds **3c, 3d, 3g, 3h, 5a, 5d** and **5e**, have significant enzyme inhibitory action (against both enzymes). It is therefore proposed that chemical scaffolds have generated novel single molecules that exert antitubercular activity, at least partly through targeting DHFR and ENR-enzymes. We anticipate that the analogues disclosed in this work will aid global efforts to identify prospective lead compounds for further development of the novel entities with dual DHFR and ENR-enzyme inhibitory properties.

## 5. Future implications

There are several important steps to consider for advancing the development of dual inhibitors in tuberculosis treatment. These steps may involve in vivo studies to evaluate pharmacokinetics, efficacy, and safety; enhancing compound properties to improve potency and selectivity; exploring new biological targets for dual inhibition; addressing drug resistance and developing countermeasures; assessing toxicity and safety profiles; studying the pharmacodynamics and pharmacokinetics of dual inhibitors; and performing preclinical efficacy studies in appropriate animal models. These research directions aim to advance the development of effective and safe dual inhibitors, addressing drug resistance and improving treatment outcomes for tuberculosis.

## Supporting information

S1 Graphical abstract (TIF)

S1 File The supplementary files contains the spectral data for different synthesized compounds. (DOC)
