# Supplementary figures and images for: Synthesis, molecular docking study and biological evaluation of new pyrrole scaffolds as potential antitubercular agents for dual targeting of enoyl ACP reductase and dihydrofolate reductase
Source: PLoS One. 2024 May 13;19(5):e0303173. doi: 10.1371/journal.pone.0303173 (PMC11090339; doi:10.1371/journal.pone.0303173)

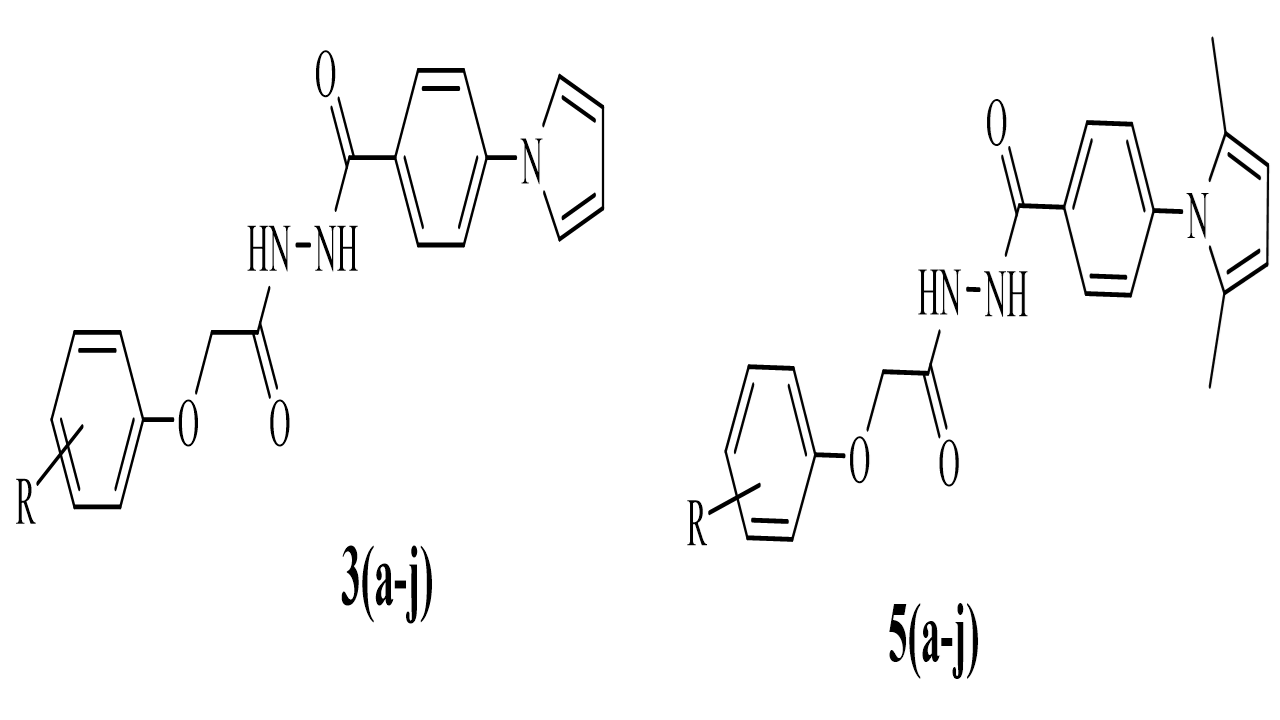

Supplement: S1 Graphical abstract — (TIF) [file pone.0303173.s001.tif]
