# Supplementary material for: Synthesis, molecular docking study and biological evaluation of new pyrrole scaffolds as potential antitubercular agents for dual targeting of enoyl ACP reductase and dihydrofolate reductase
Source: PLoS One. 2024 May 13;19(5):e0303173. doi: 10.1371/journal.pone.0303173 (PMC11090339; doi:10.1371/journal.pone.0303173)

Synthesis, Molecular Docking Study and Biological Evaluation of New Pyrrole Scaffolds as Potential Antitubercular Agents for Dual Targeting of Enoyl ACP Reductase and Dihydrofolate Reductase

Mater H. Mahnashi^1^, Sravanthi Avunoori^2^, Sanjay Gopi^2^, Ibrahim Ahmed Shaikh^3^, Ahmed Saif^4^, Farkad Bantun^5^, Hani Saleh Faidah^6^, Abdulrahman Ali Alhadi^7^, Jaber Hassan Alshehri^8^, Abdullah Ali Alharbi^8^, Prem Kumar S. R^9^, Shrinivas D. Joshi^2^*

^1^Department of Pharmaceutical Chemistry, College of Pharmacy, Najran University, Najran 66462, Saudi Arabia, [matermaha@gmail.com](mailto:matermaha@gmail.com)

^2^Novel Drug Design and Discovery Laboratory, Department of Pharmaceutical Chemistry, SET’s College of Pharmacy, Sangolli Rayanna Nagar, Dharwad-580 002, Karnataka, India; [sravanthi.avunoori@gmail.com](mailto:sravanthi.avunoori@gmail.com), [prempharma77@gmail.com](mailto:prempharma77@gmail.com),

^3^ Department of Pharmacology, College of Pharmacy, Najran University, Najran 66462, Saudi Arabia, [i.ibrahimshaikh09@gmail.com](mailto:i.ibrahimshaikh09@gmail.com)

^4^ Department of Clinical Laboratory Sciences, College of Applied Medical Sciences, King Khalid University,

Abha, Saudi Arabia; [amsaif8080@gmail.com](mailto:amsaif8080@gmail.com)

^5^ Department of Microbiology, Faculty of Medicine, Umm Al-Qura University, Makkah, Saudi Arabia; [fmbantun@uqu.edu.sa](mailto:fmbantun@uqu.edu.sa)

^6^ Department of Microbiology Faculty of Medicine Umm Al-Qura University, P.O. Box 715 Holy Makkah 21955 Kingdom of Saudi Arabia; [hsfaidah@uqu.edu.sa](mailto:hsfaidah@uqu.edu.sa)

^7^ Department of Microbiology Faculty of Medicine Umm Al-Qura University, P.O. Box 715 Holy Makkah 21955 Kingdom of Saudi Arabia; [abd.rah999@hotmail.com](mailto:abd.rah999@hotmail.com)

^8^ Microbiology Section Pathology and Laboratory Medicine, Armed Forces Hospital Southern Region,

Kingdom of Saudi Arabia; [jaber.sh.16@gmail.com](mailto:jaber.sh.16@gmail.com); [aaha1415@hotmail.com](mailto:aaha1415@hotmail.com)

^9^ Department of Pharmaceutical Chemistry, MVM College of Pharmacy, Yelahanka, Bengaluru-560 064, Karnataka, India

*****Corresponding Author: **Dr. Shrinivas D. Joshi,** Professor and Head, Dept. of Pharmaceutical Chemistry, SET’s College of Pharmacy, S. R. Nagar, Dharwad-580002, Karnataka; Mobile: +91 9986151953; E-mail: shrinivasdj@rediffmail.com

SPECTRUM 01: IR SPECTRUM OF COMPOUND 3A


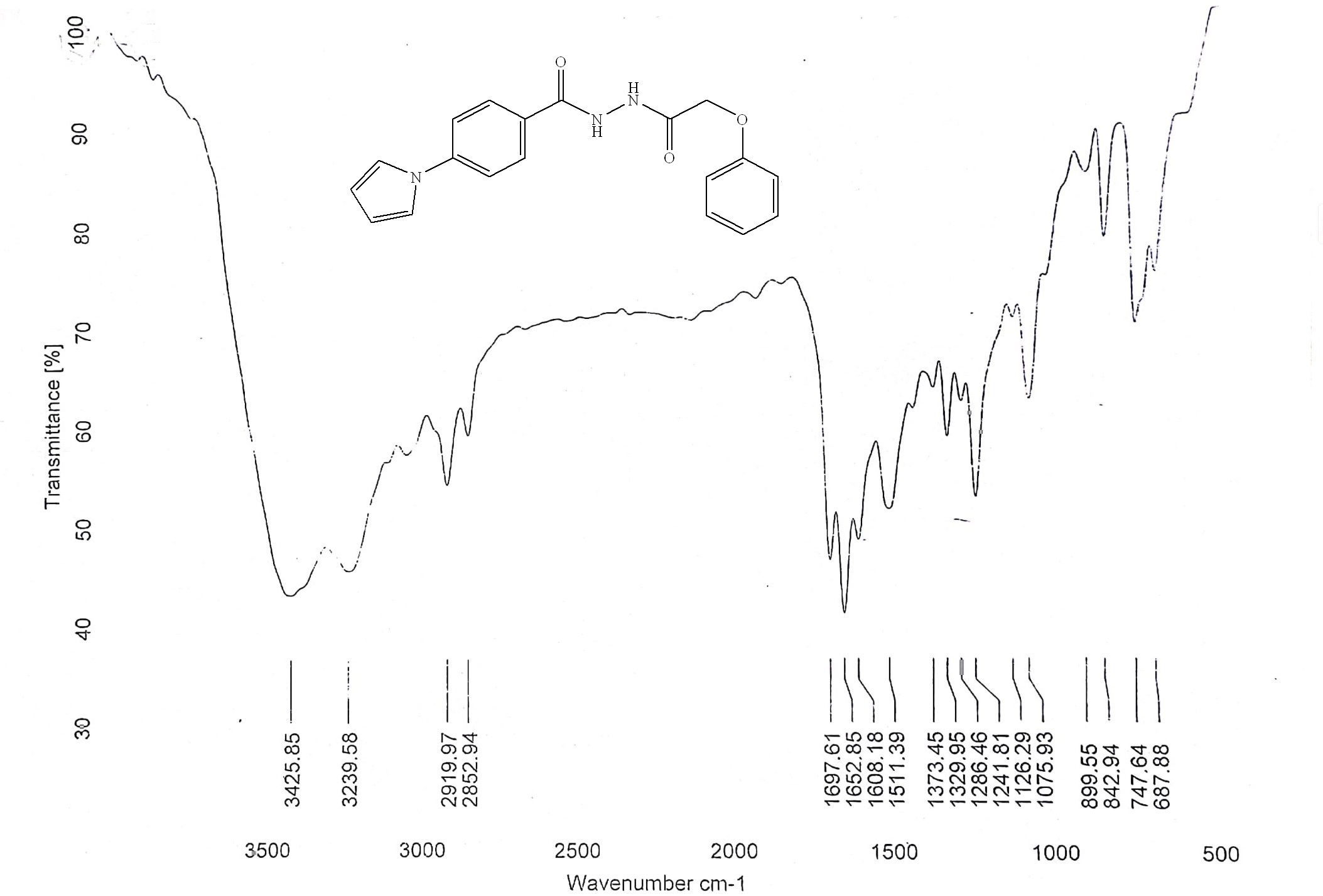


SPECTRUM 02: ^1^HNMR SPECTRUM OF COMPOUND 3A


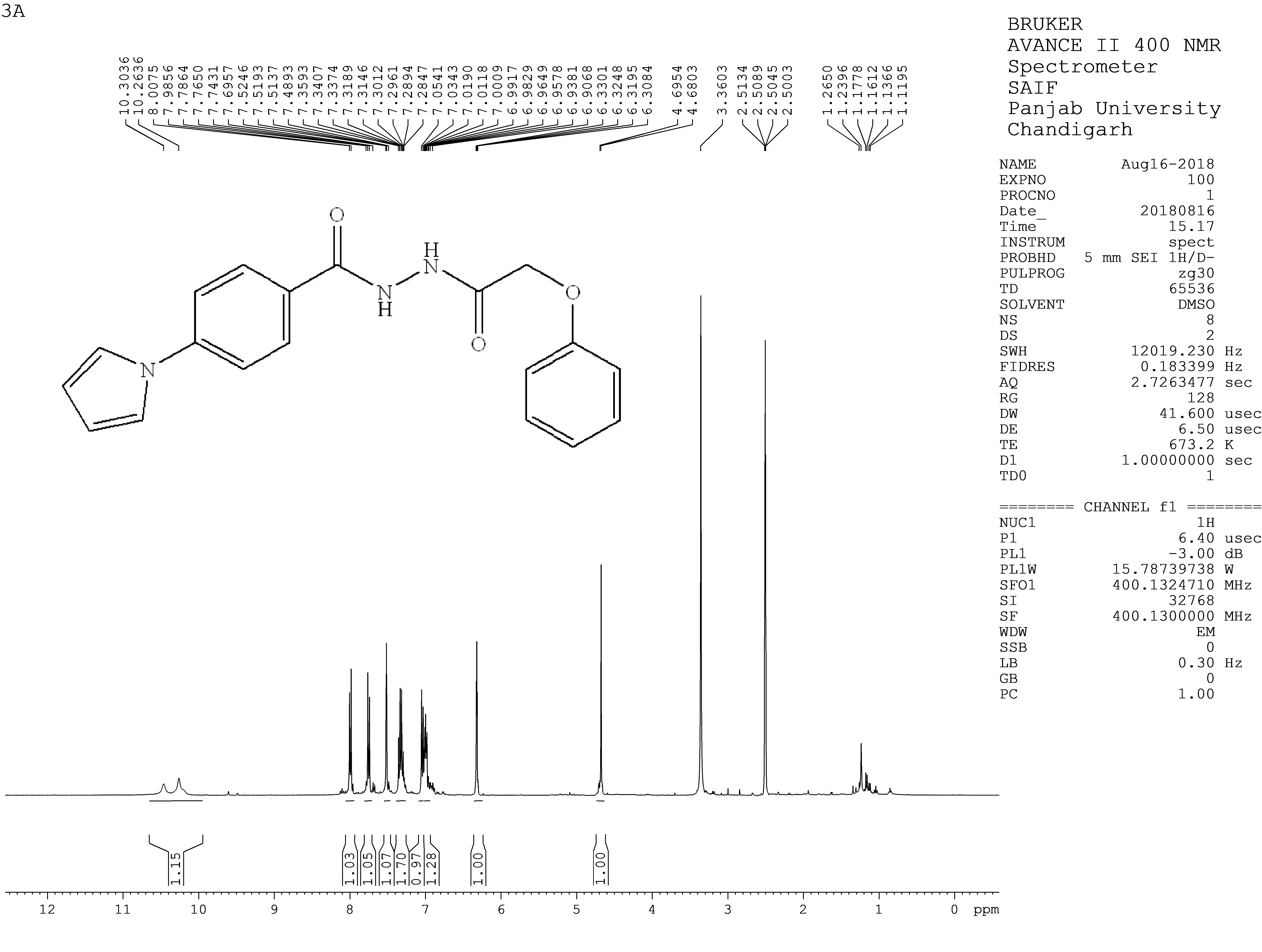


SPECTRUM 03: ^13^ C NMR SPECTRUM OF COMPOUND 3A


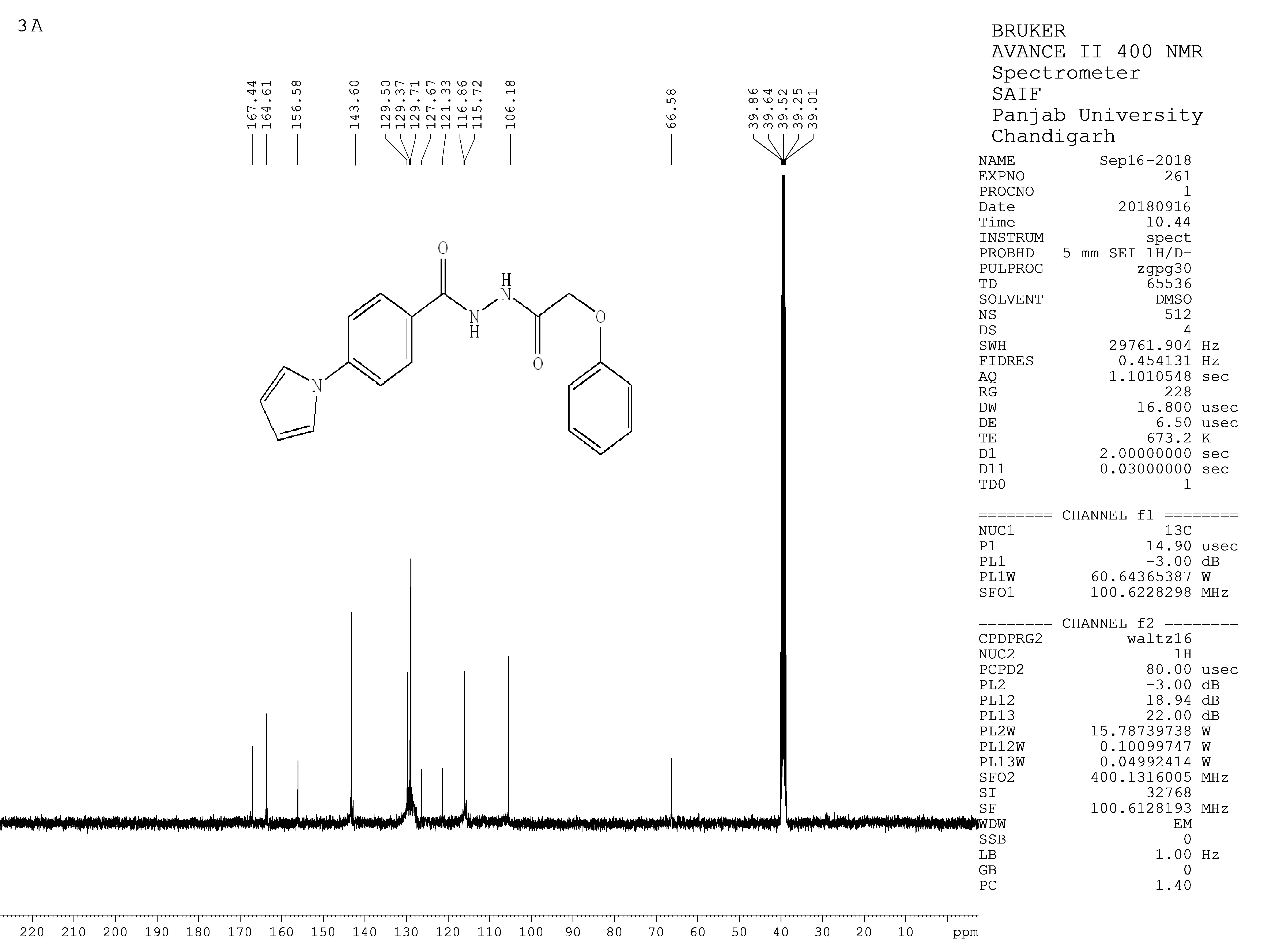


SPECTRUM 04: MASS SPECTRUM OF COMPOUND 3A


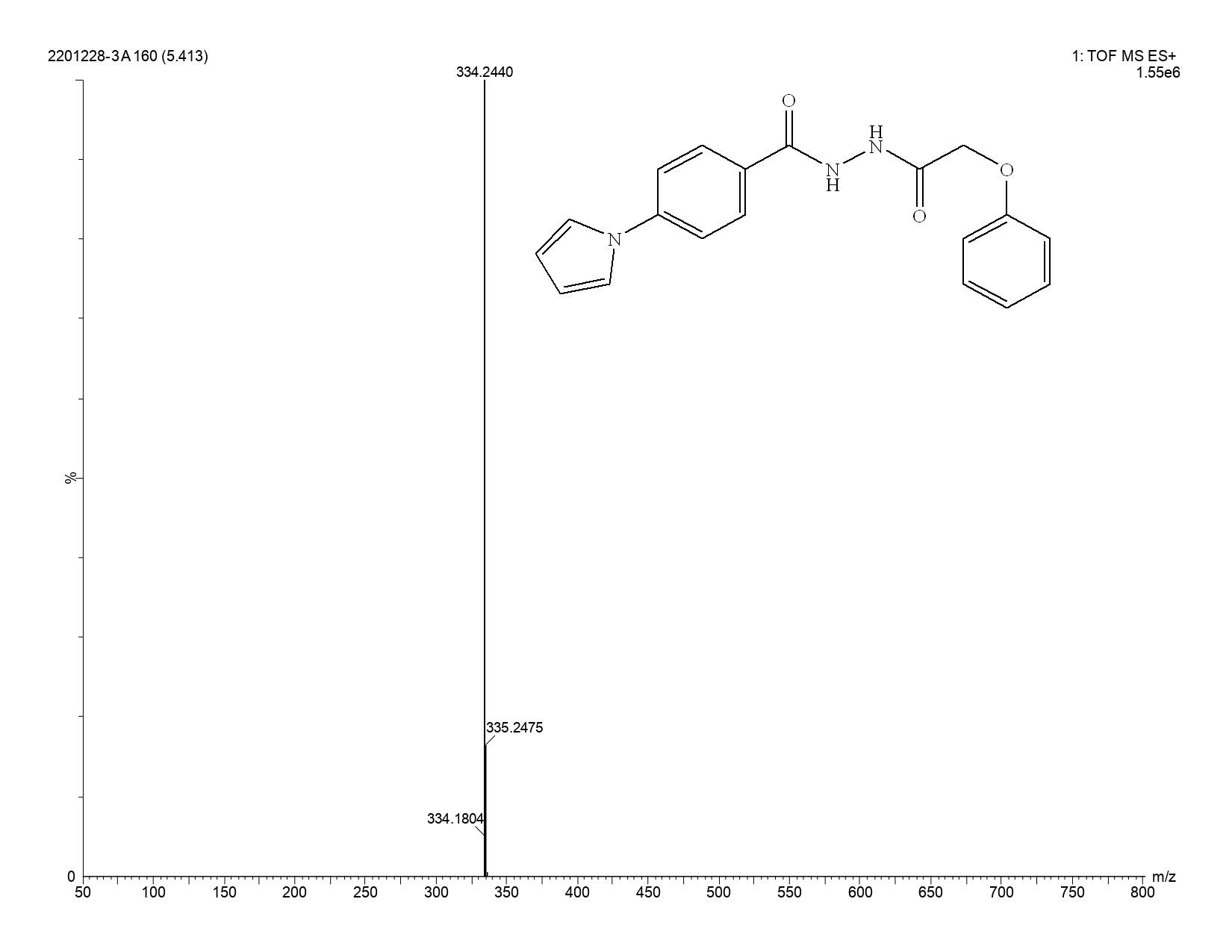


SPECTRUM 05: IR SPECTRUM OF COMPOUND 3B


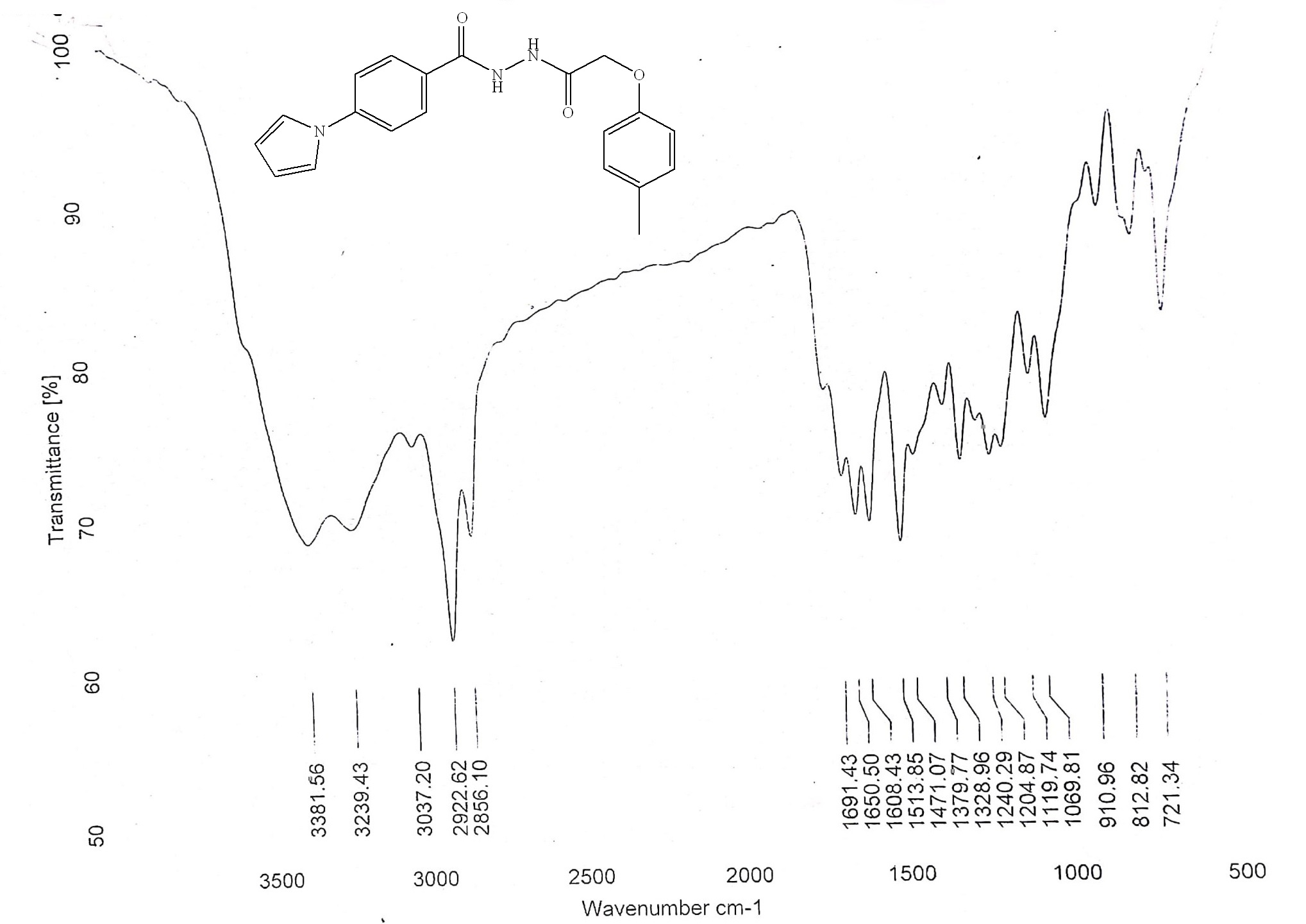


SPECTRUM 06: ^1^HNMR SPECTRUM OF COMPOUND 3B


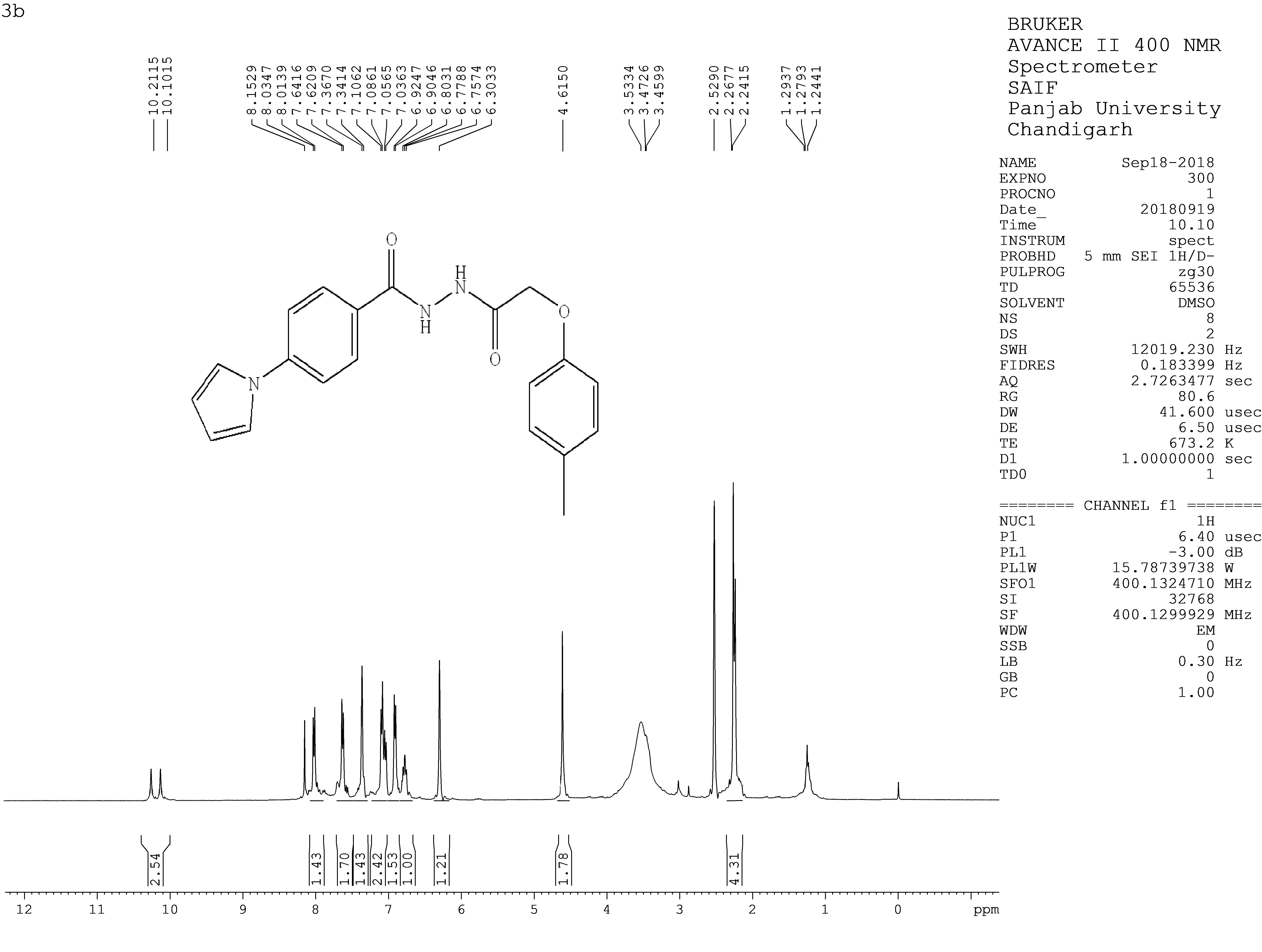


SPECTRUM 07: MASS SPECTRUM OF COMPOUND 3B


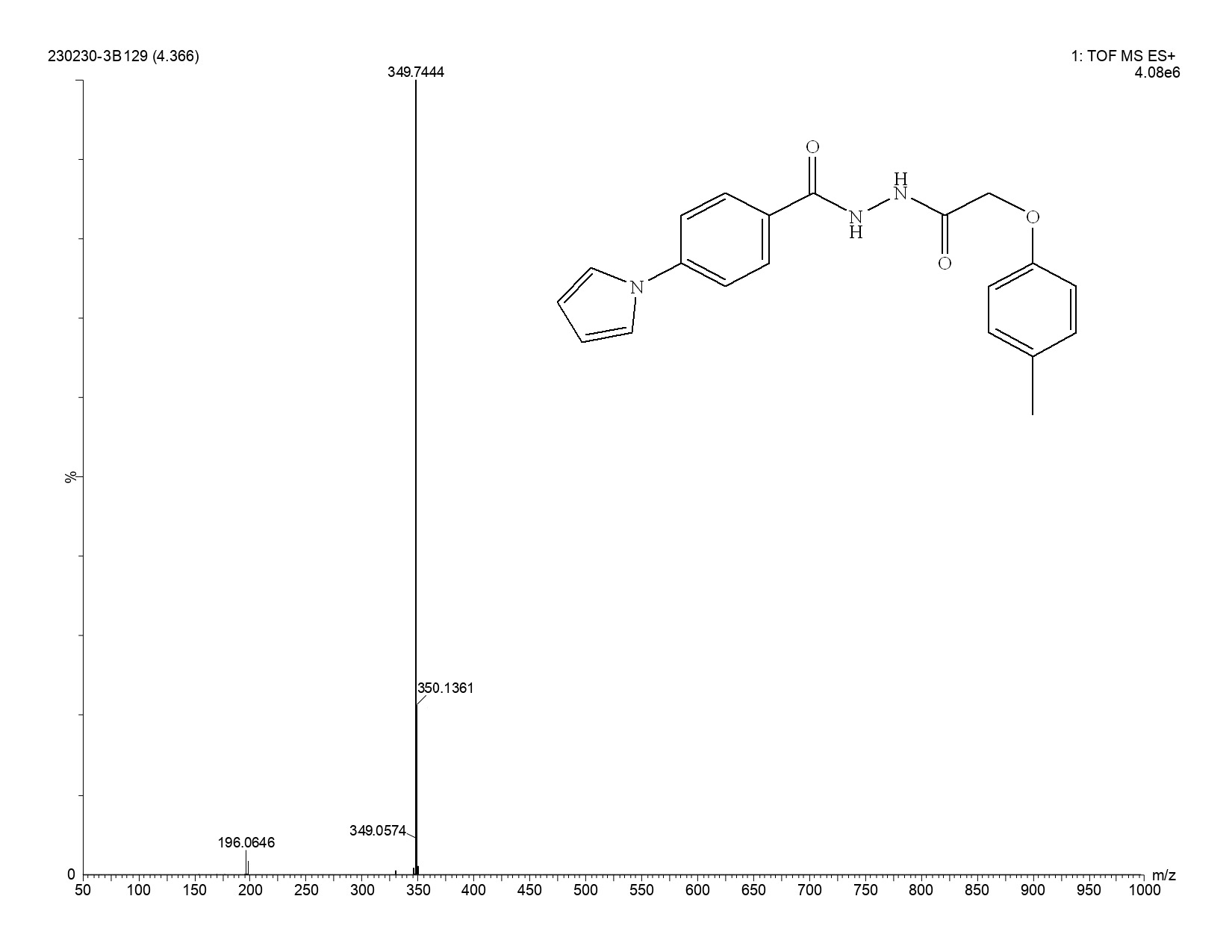


SPECTRUM 08: IR SPECTRUM OF COMPOUND 3C


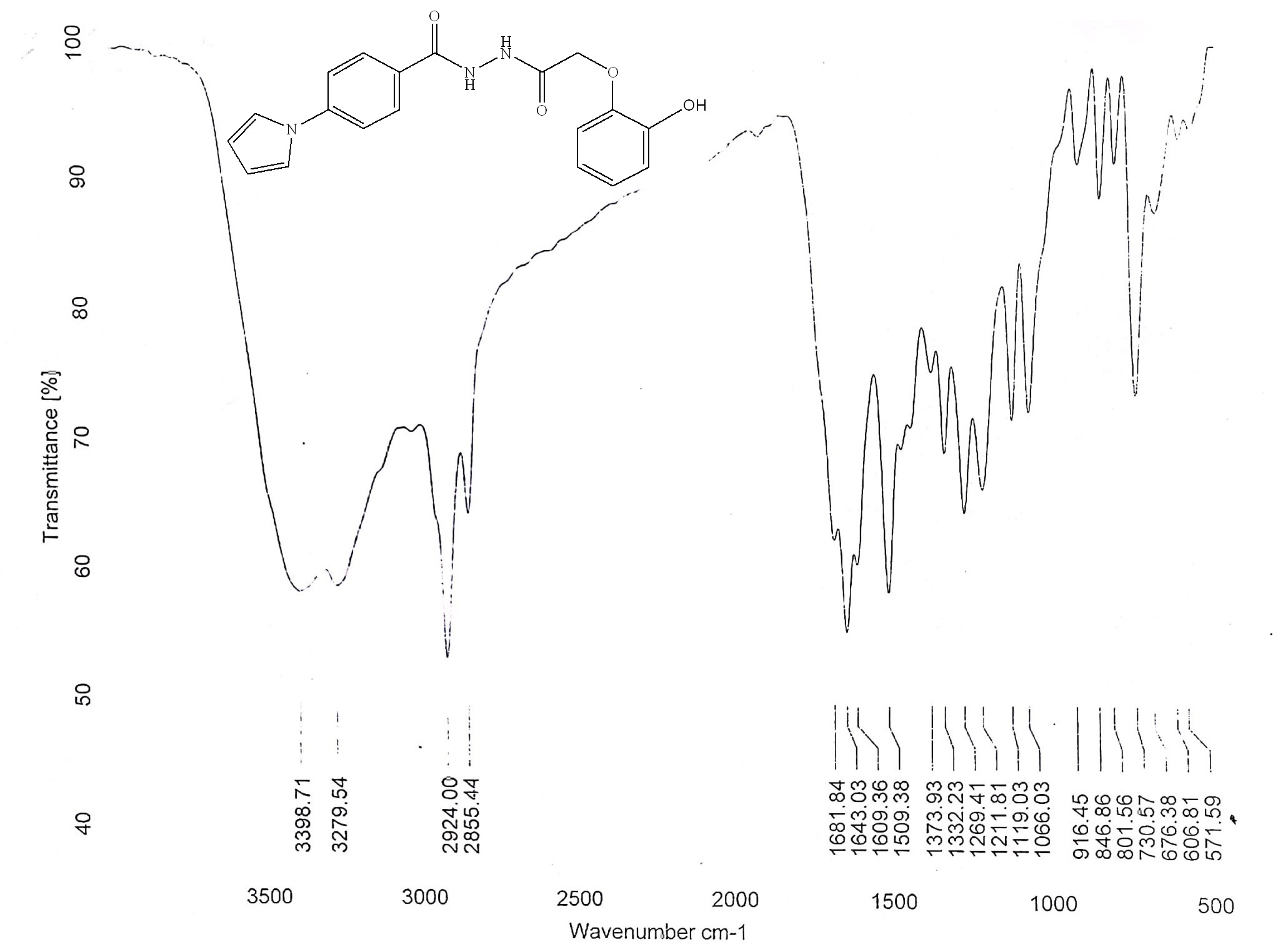


SPECTRUM 09: ^1^HNMR SPECTRUM OF COMPOUND 3C


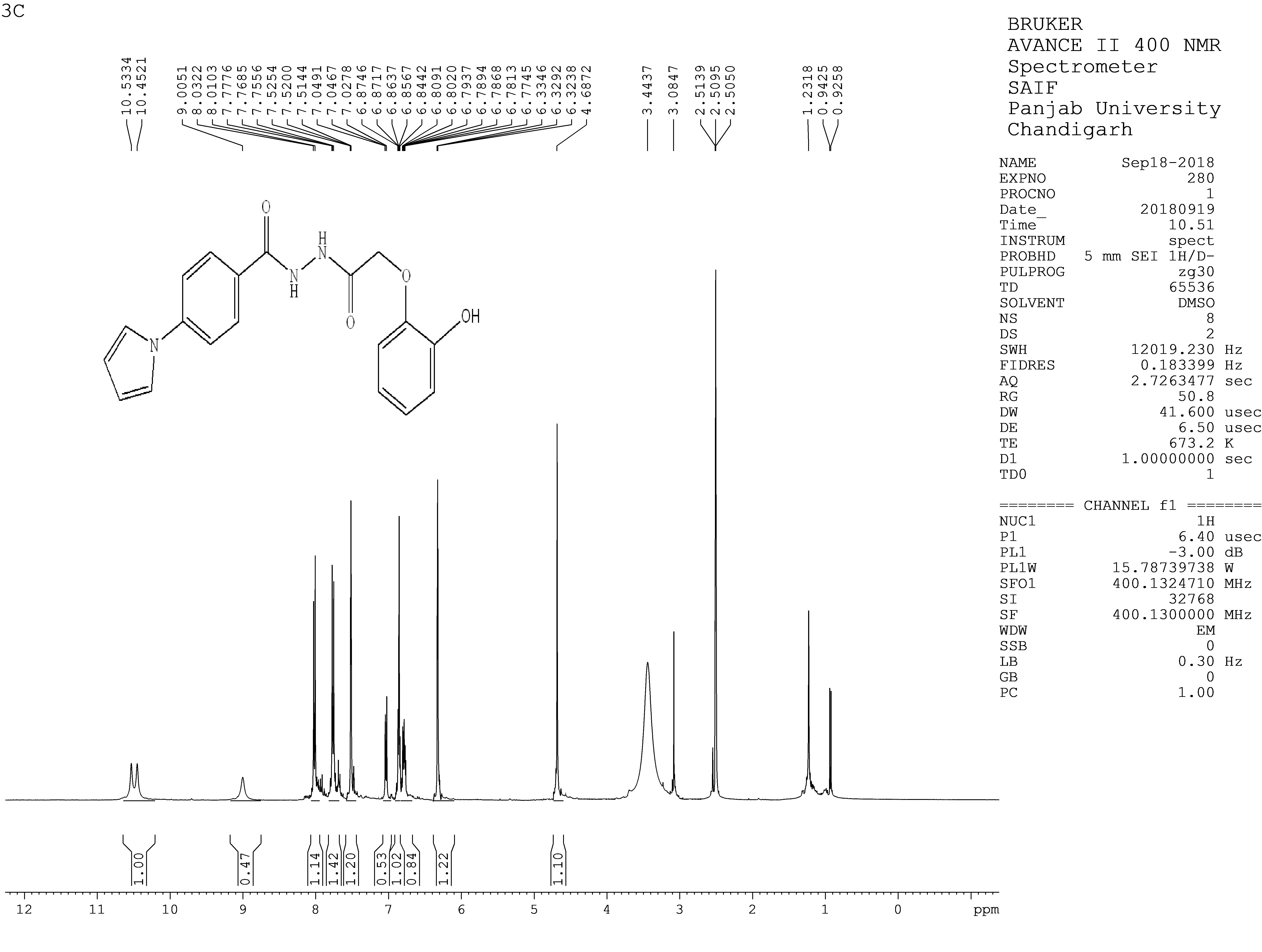


SPECTRUM 10: MASS SPECTRUM OF COMPOUND 3C


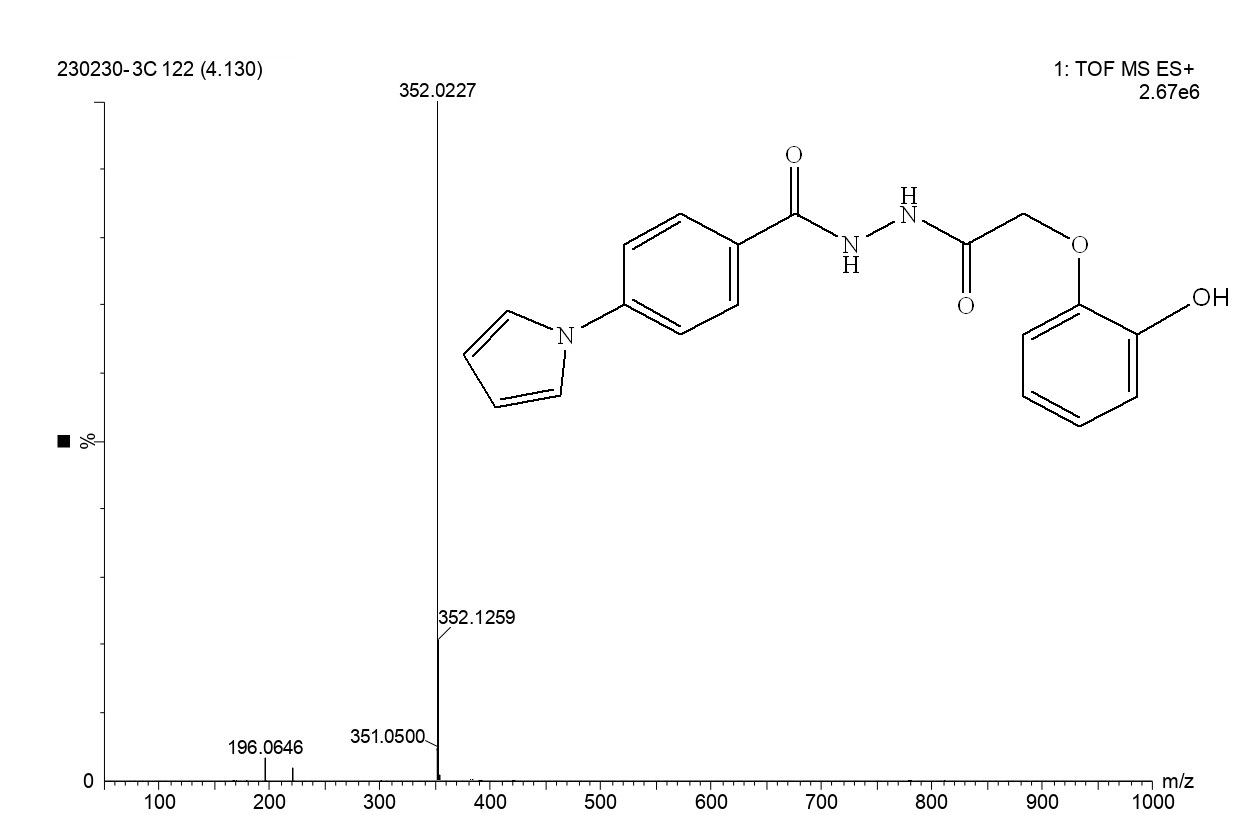


SPECTRUM 11: IR SPECTRUM OF COMPOUND 3D


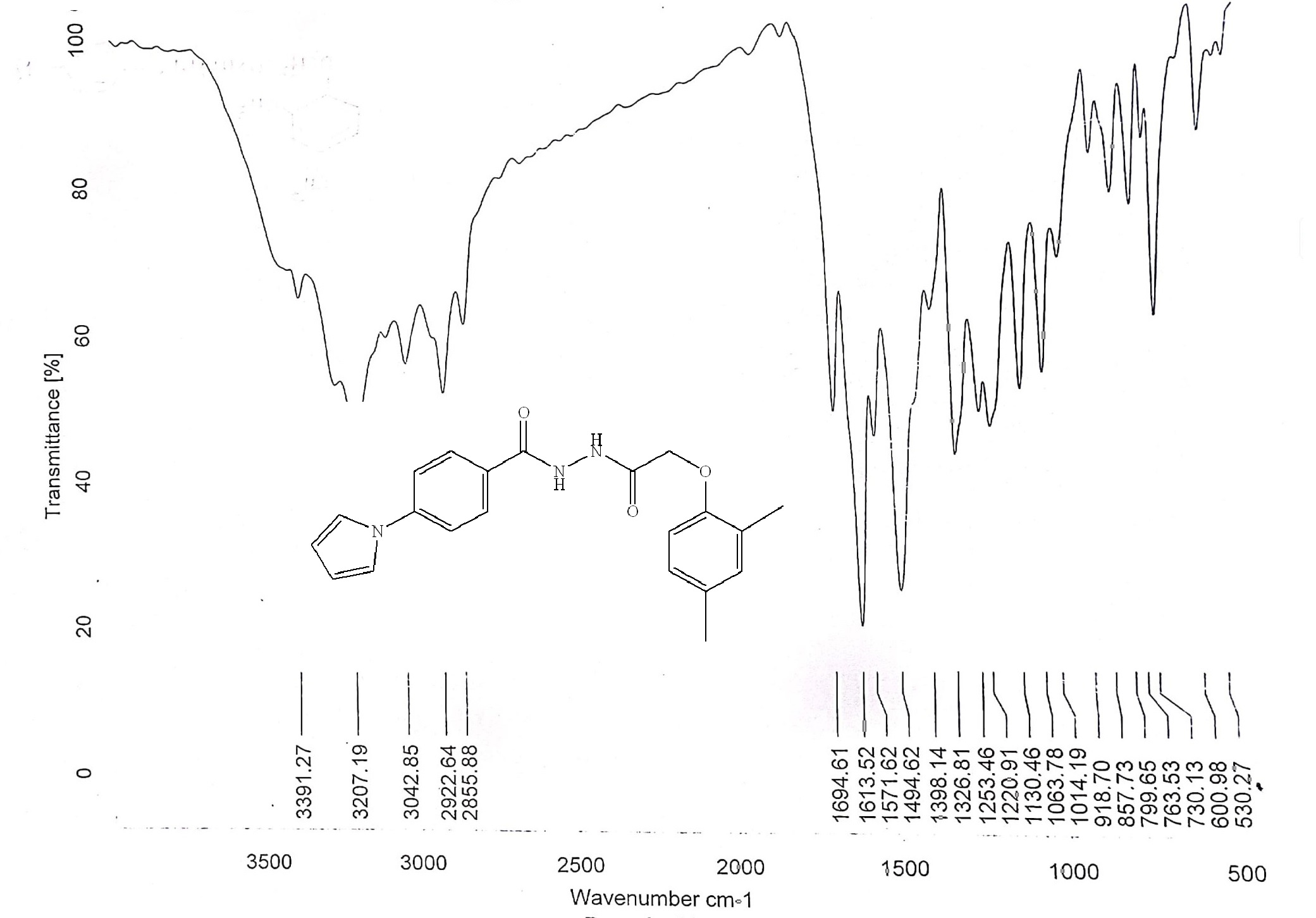


SPECTRUM 12: ^1^HNMR SPECTRUM OF COMPOUND 3D


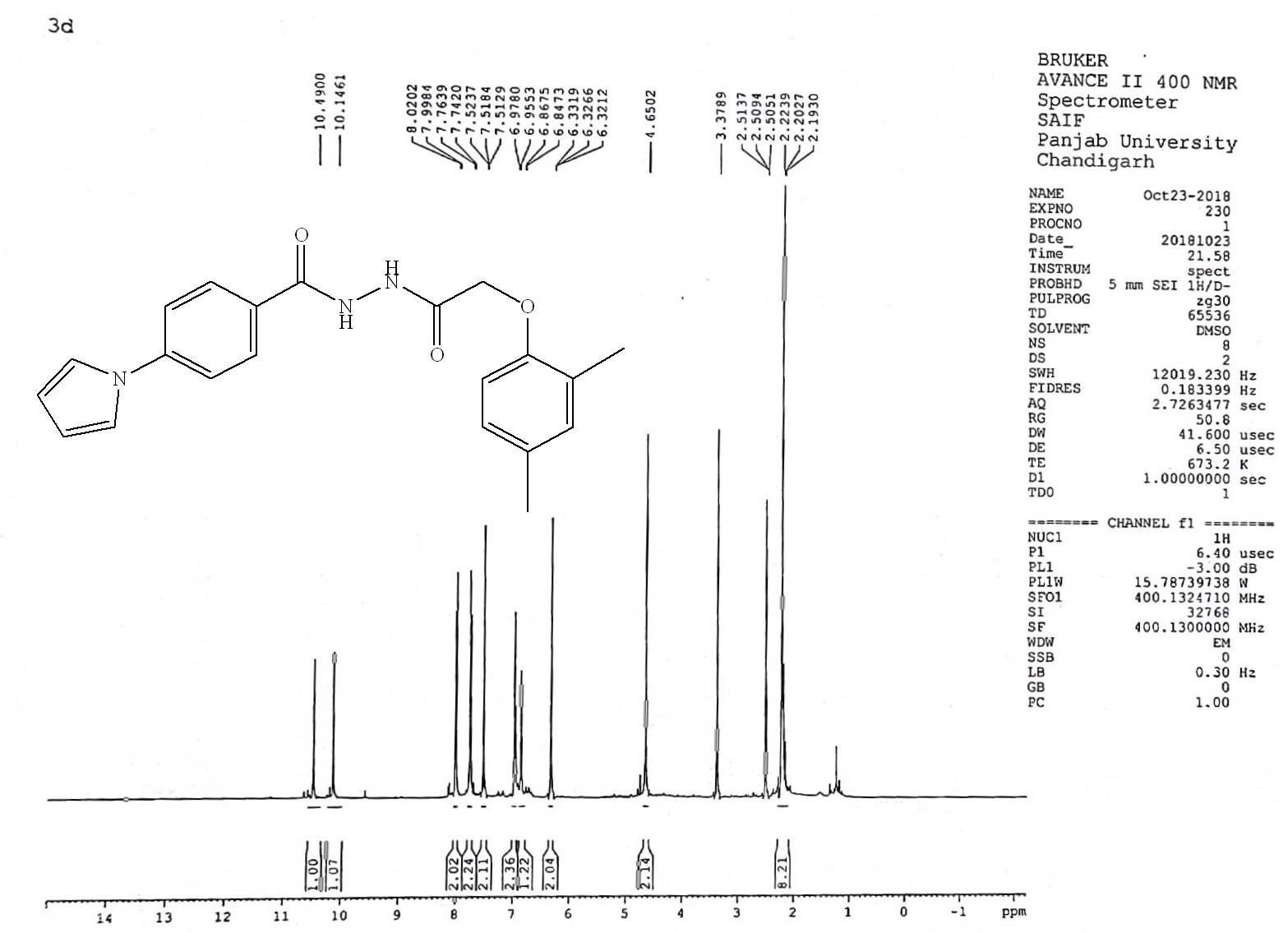


SPECTRUM 13: MASS SPECTRUM OF COMPOUND 3D


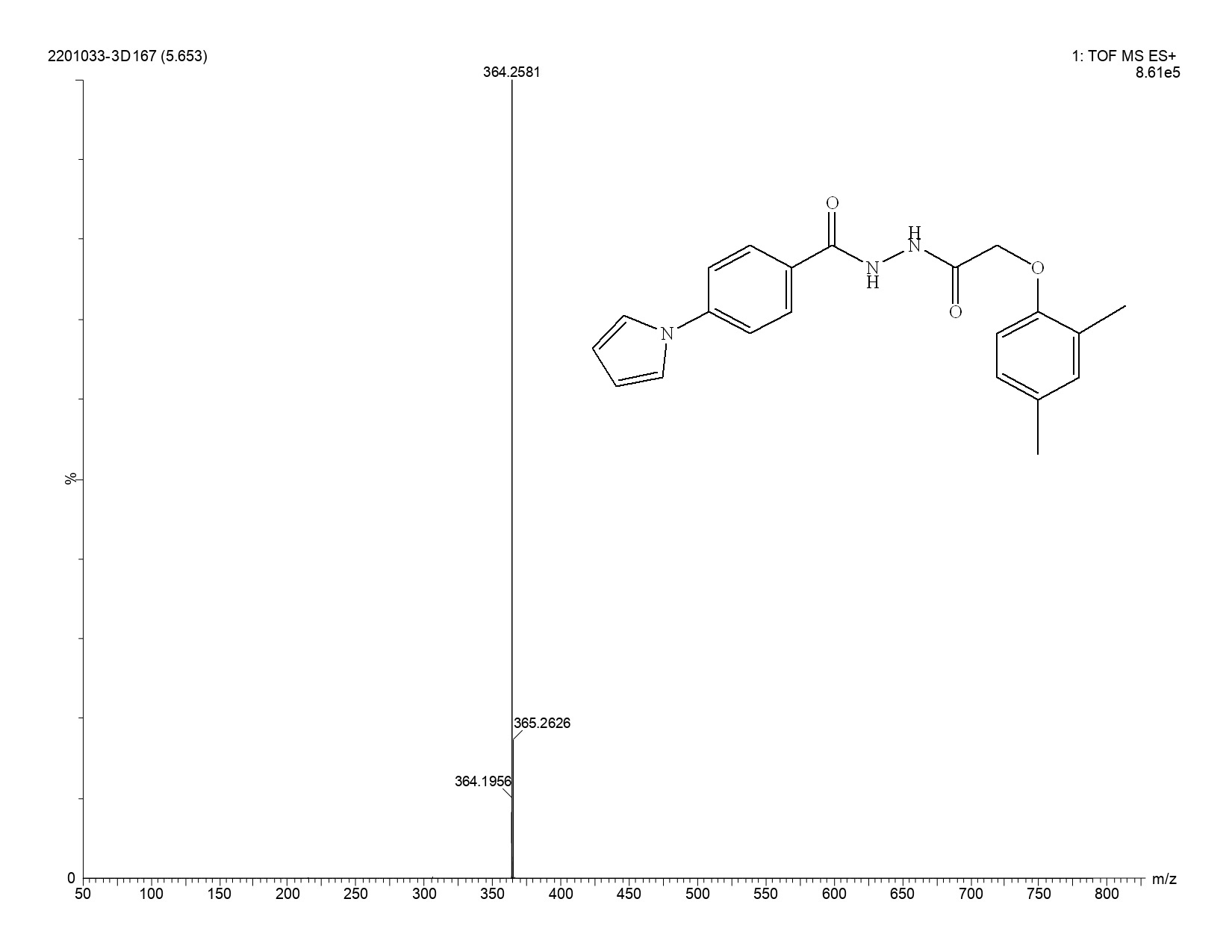


SPECTRUM 14: IR SPECTRUM OF COMPOUND 3E


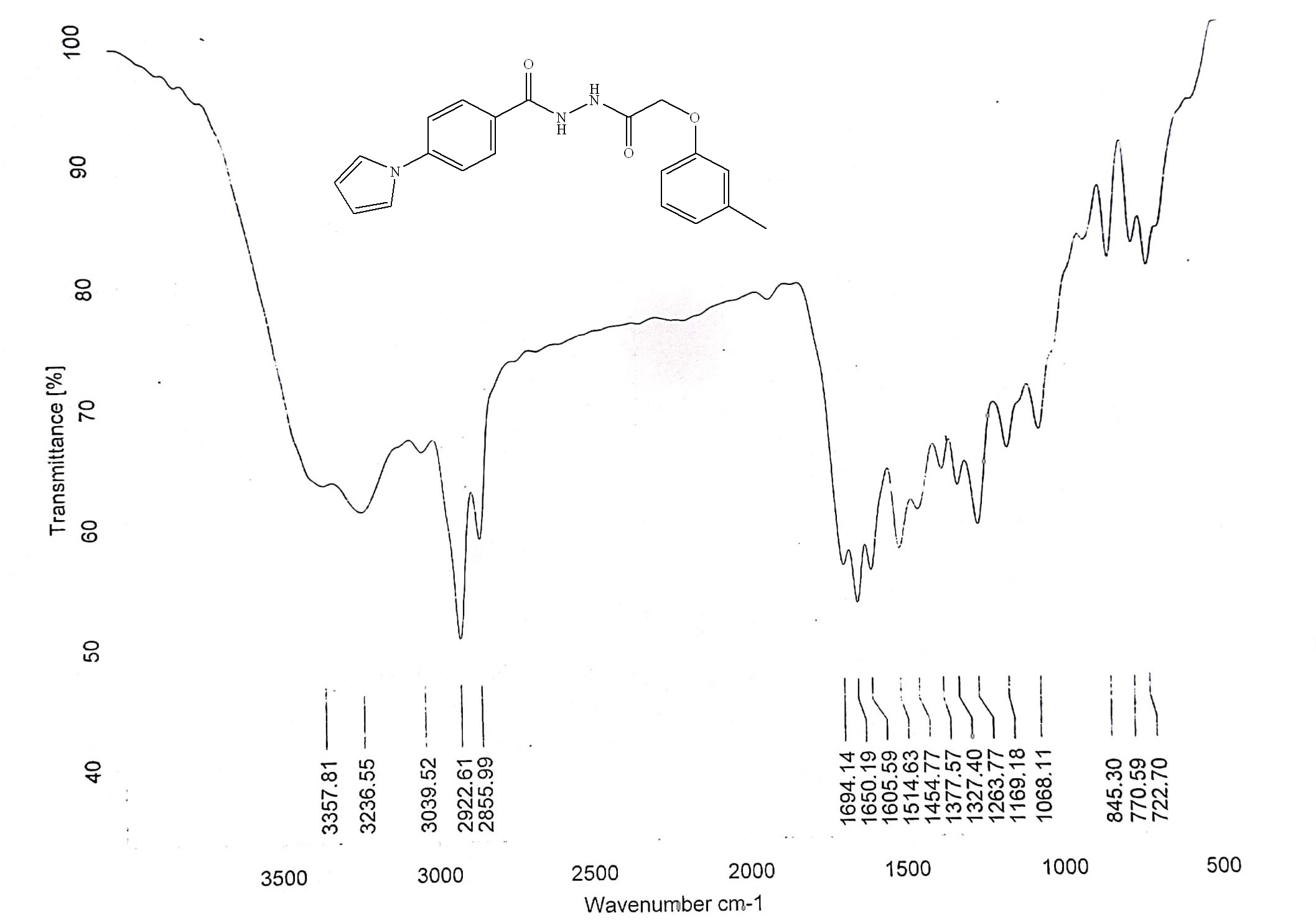


SPECTRUM 15: ^1^HNMR SPECTRUM OF COMPOUND 3E


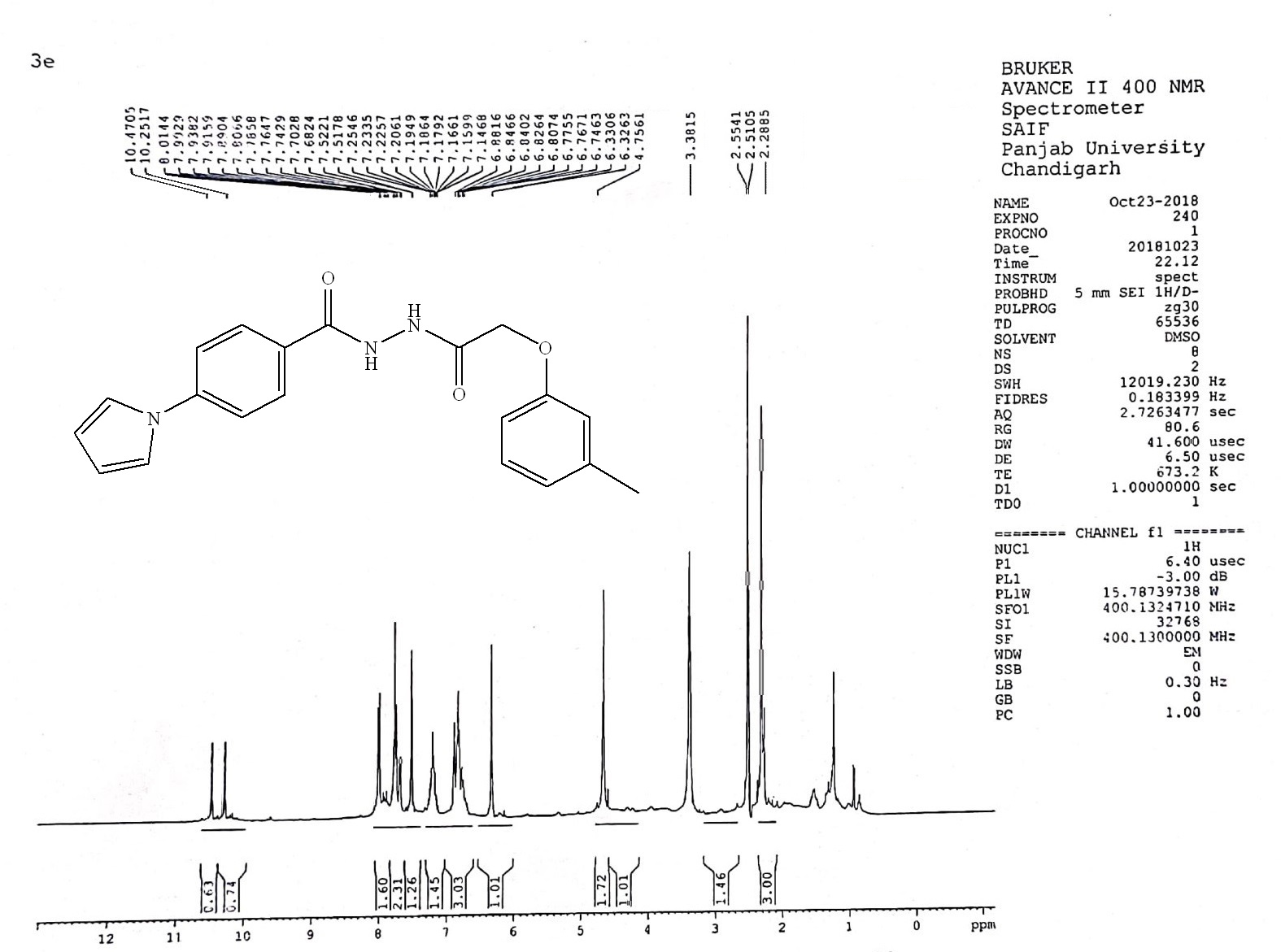


SPECTRUM 16: MASS SPECTRUM OF COMPOUND 3E


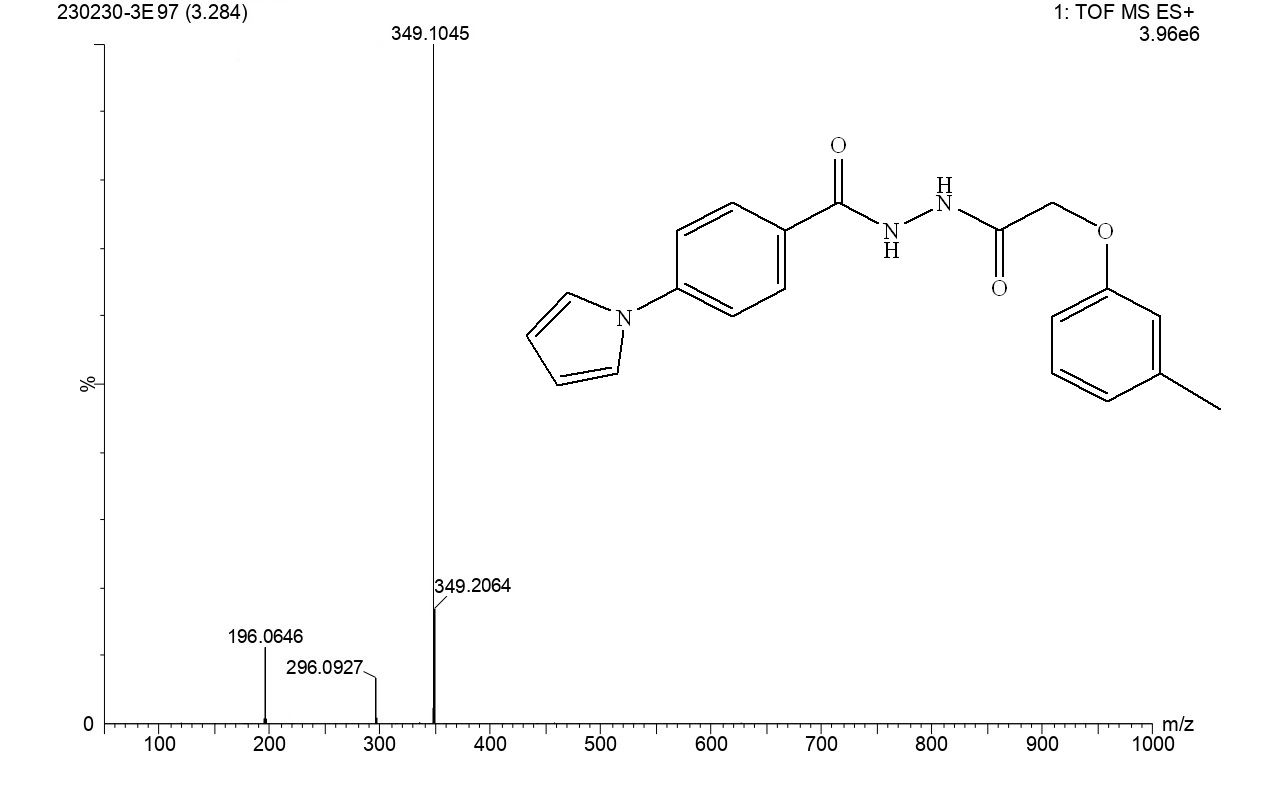


SPECTRUM 17: IR SPECTRUM OF COMPOUND 3F


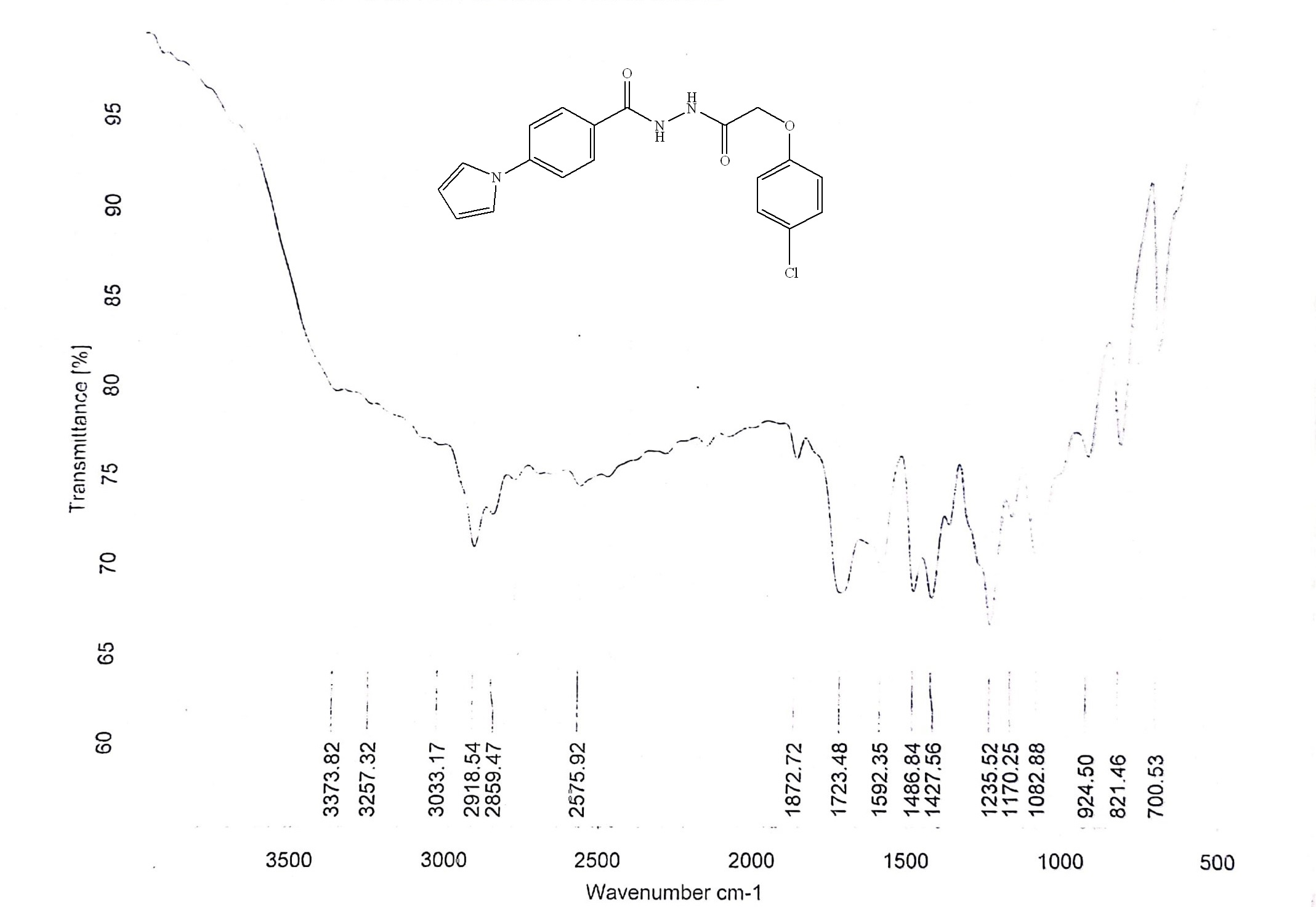


SPECTRUM 18: ^1^HNMR SPECTRUM OF COMPOUND 3F


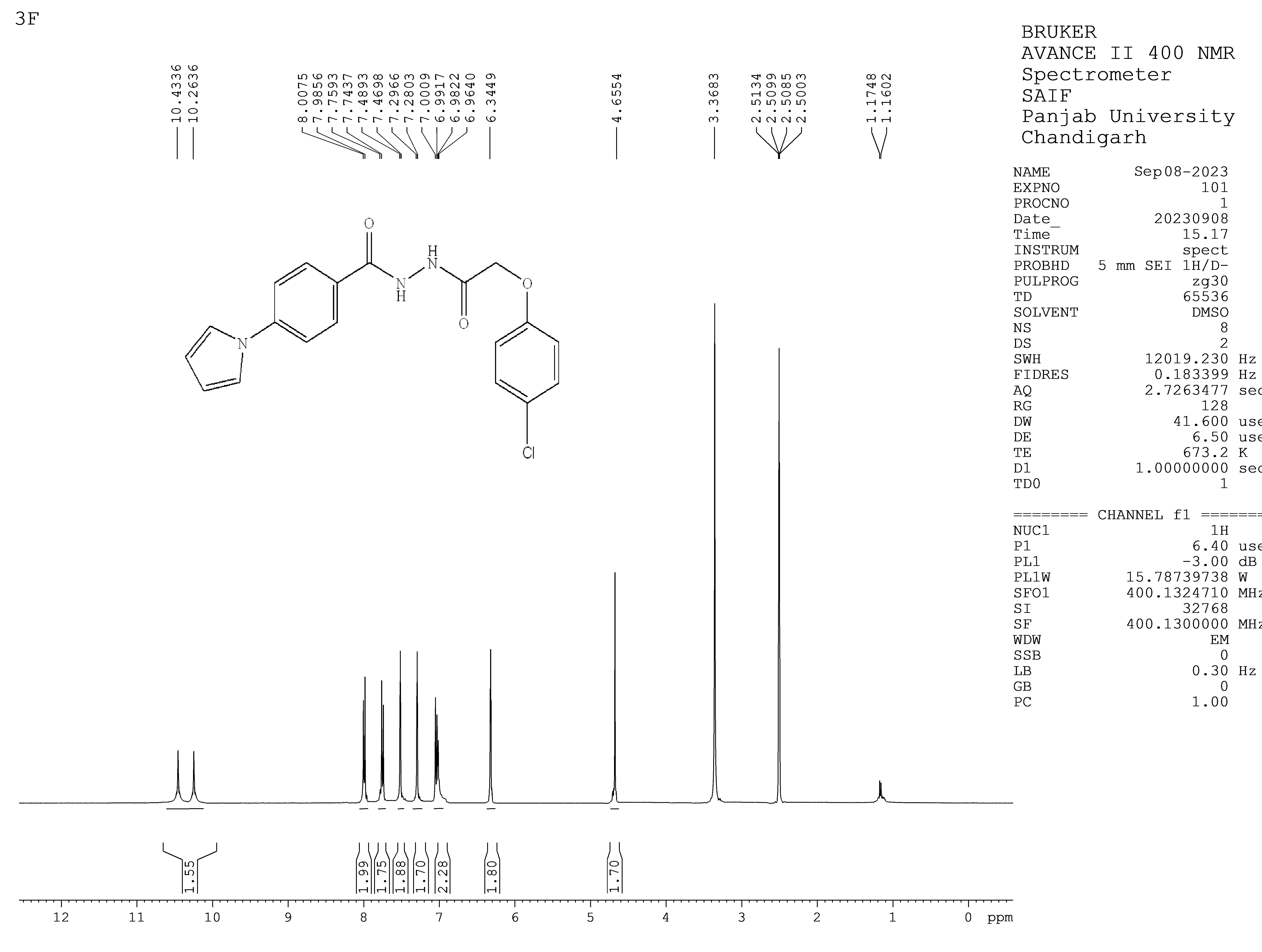


SPECTRUM 19: ^13^ C NMR SPECTRUM OF COMPOUND 3F


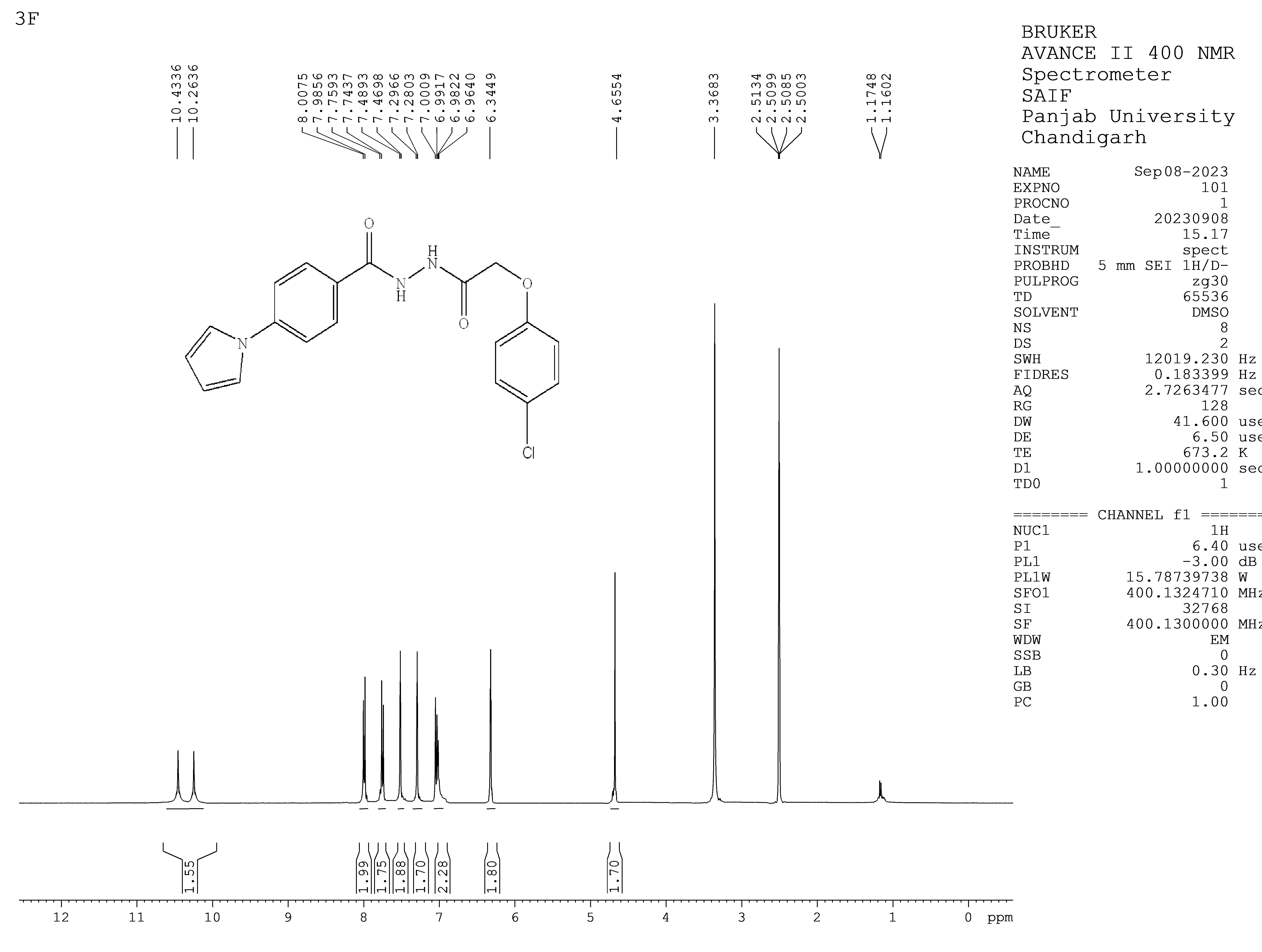


SPECTRUM 20: MASS SPECTRUM OF COMPOUND 3F


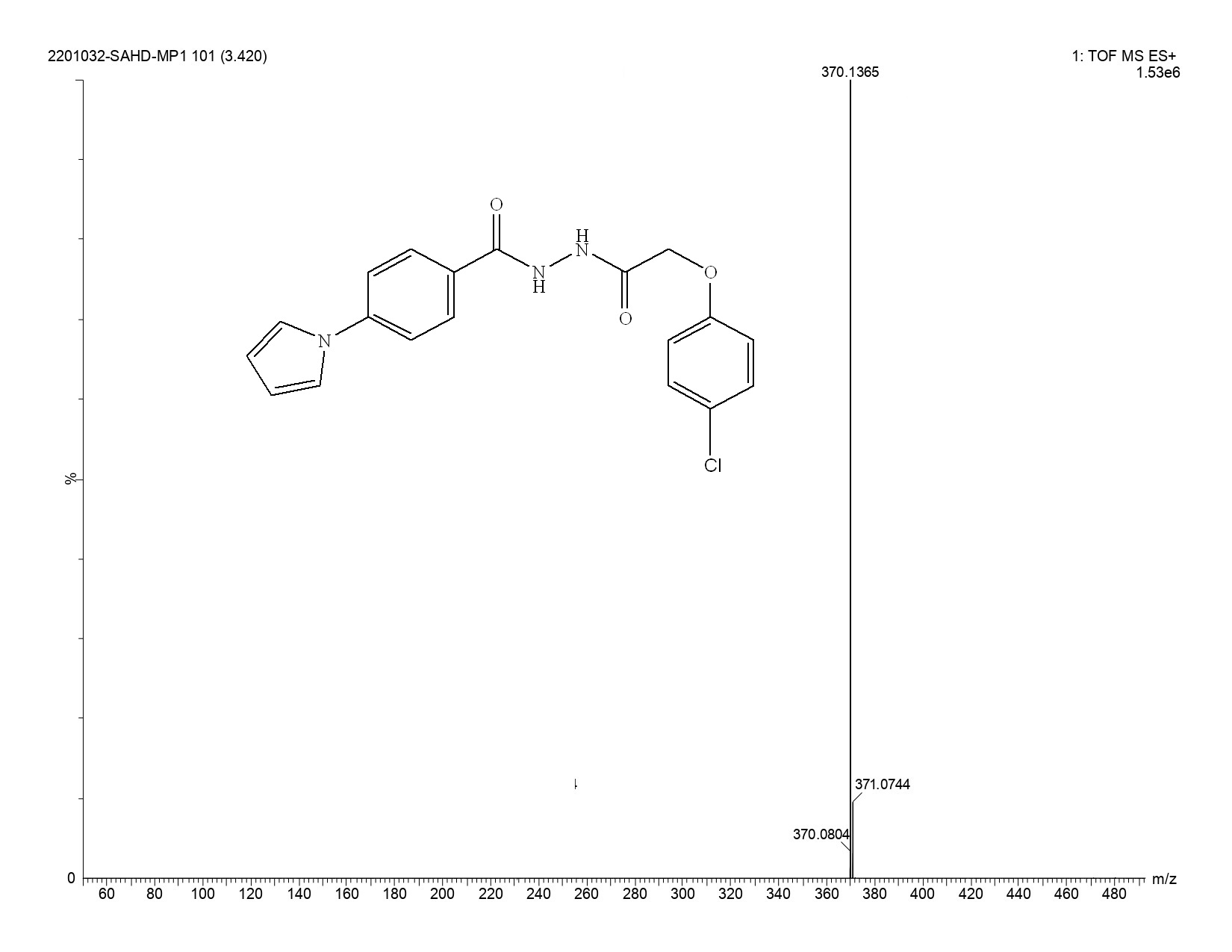


SPECTRUM 21: IR SPECTRUM OF COMPOUND 3G


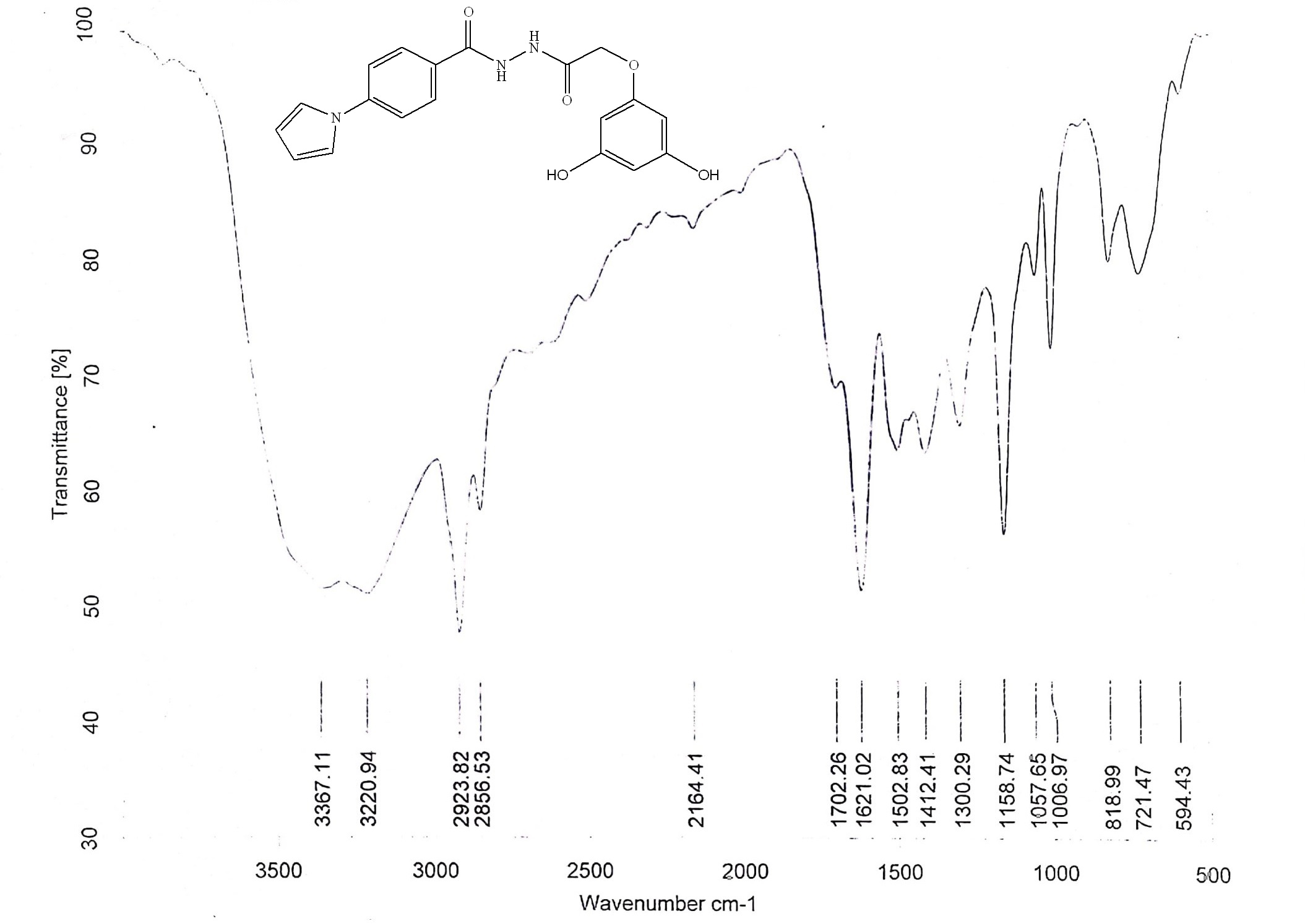


SPECTRUM 22: ^1^HNMR SPECTRUM OF COMPOUND 3G


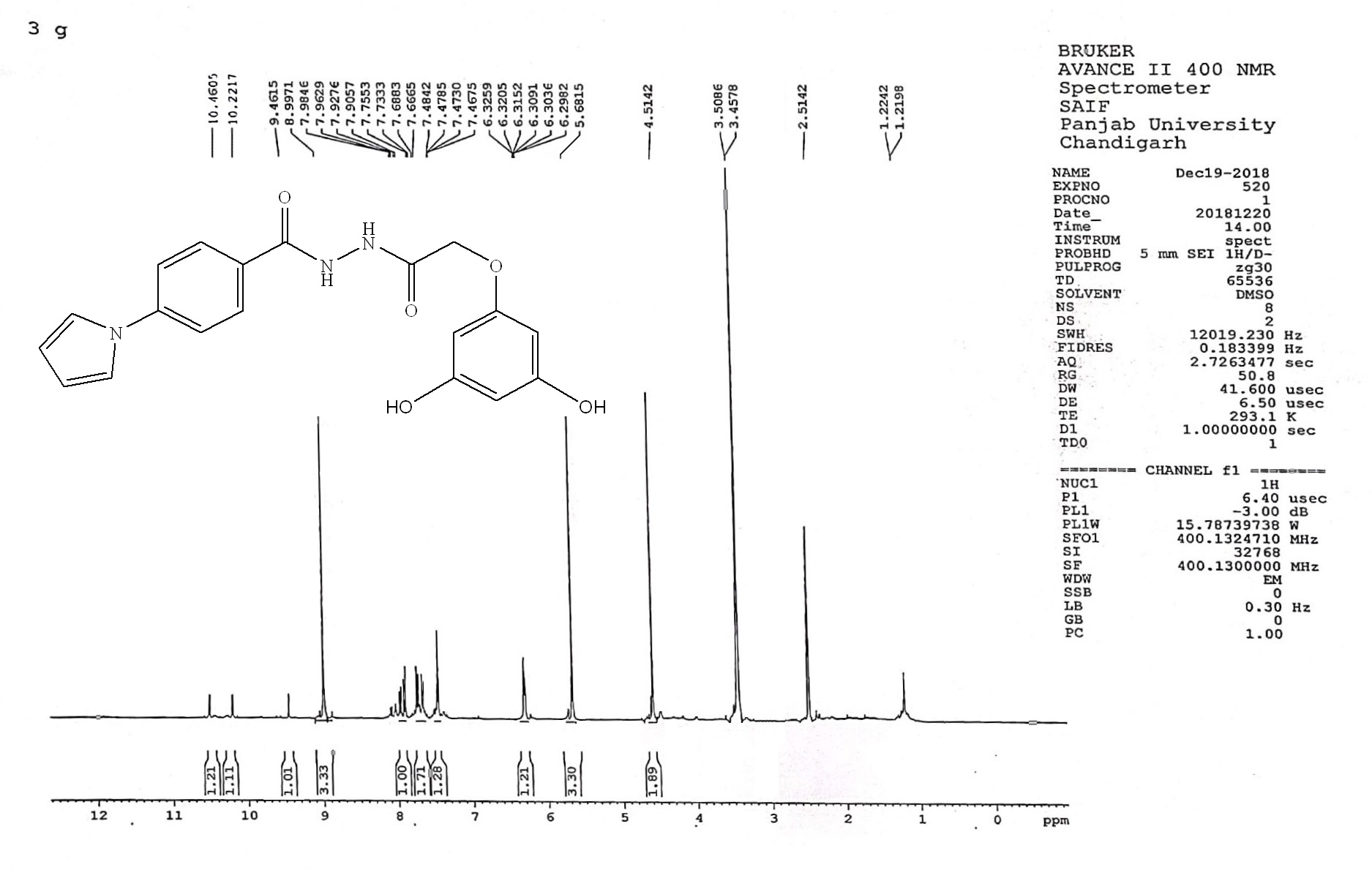


SPECTRUM 23: MASS SPECTRUM OF COMPOUND 3G


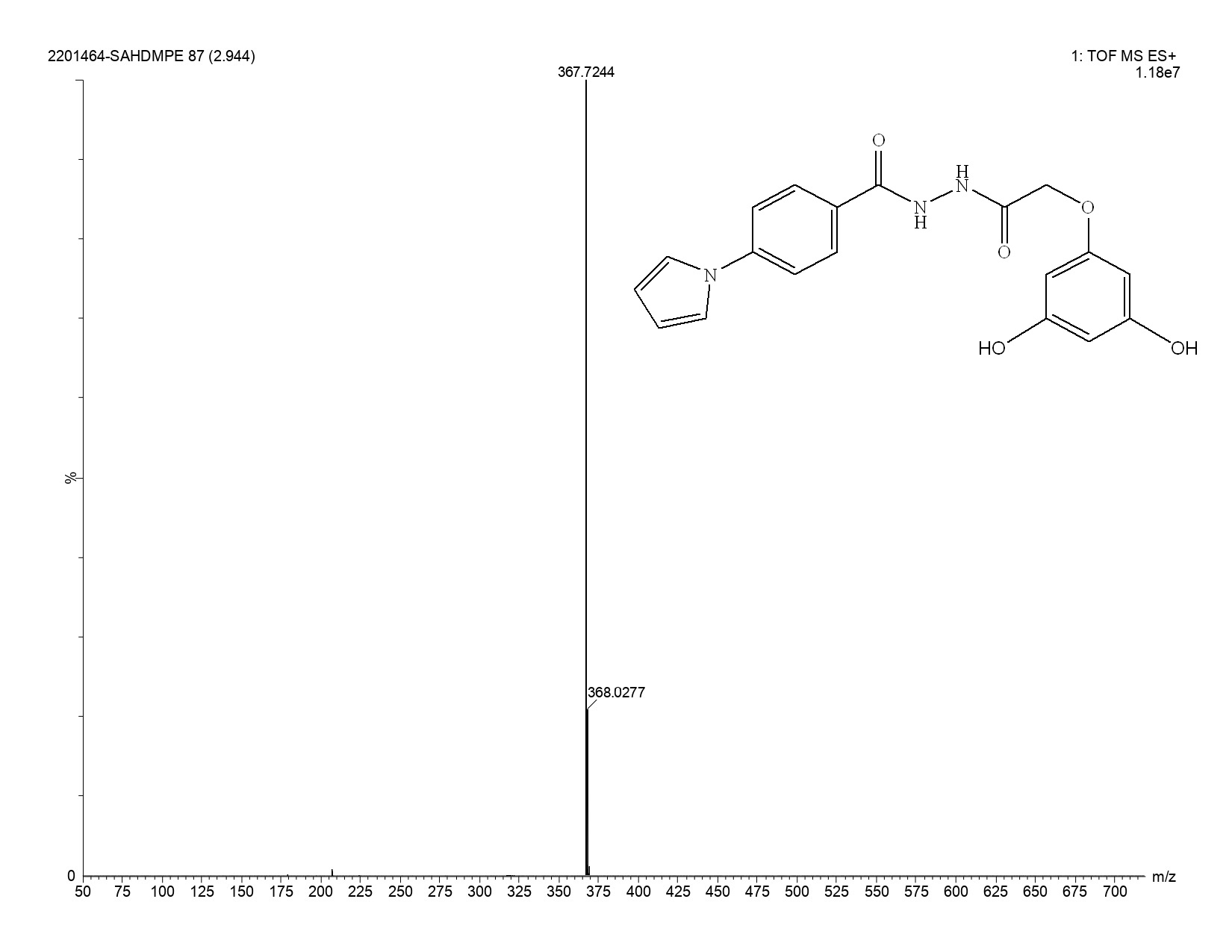


SPECTRUM 24: IR SPECTRUM OF COMPOUND 3H


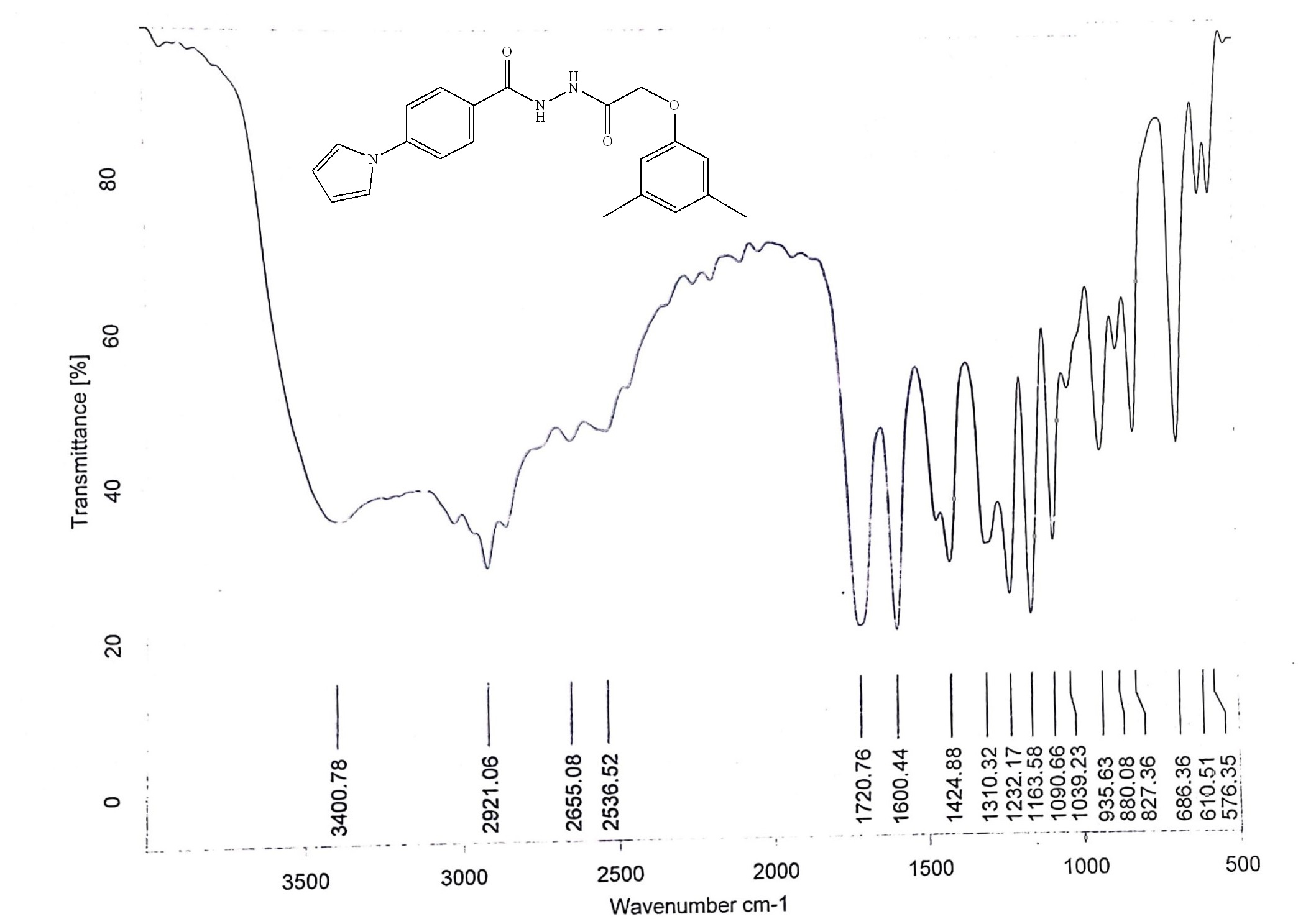


SPECTRUM 25: ^1^HNMR SPECTRUM OF COMPOUND 3H


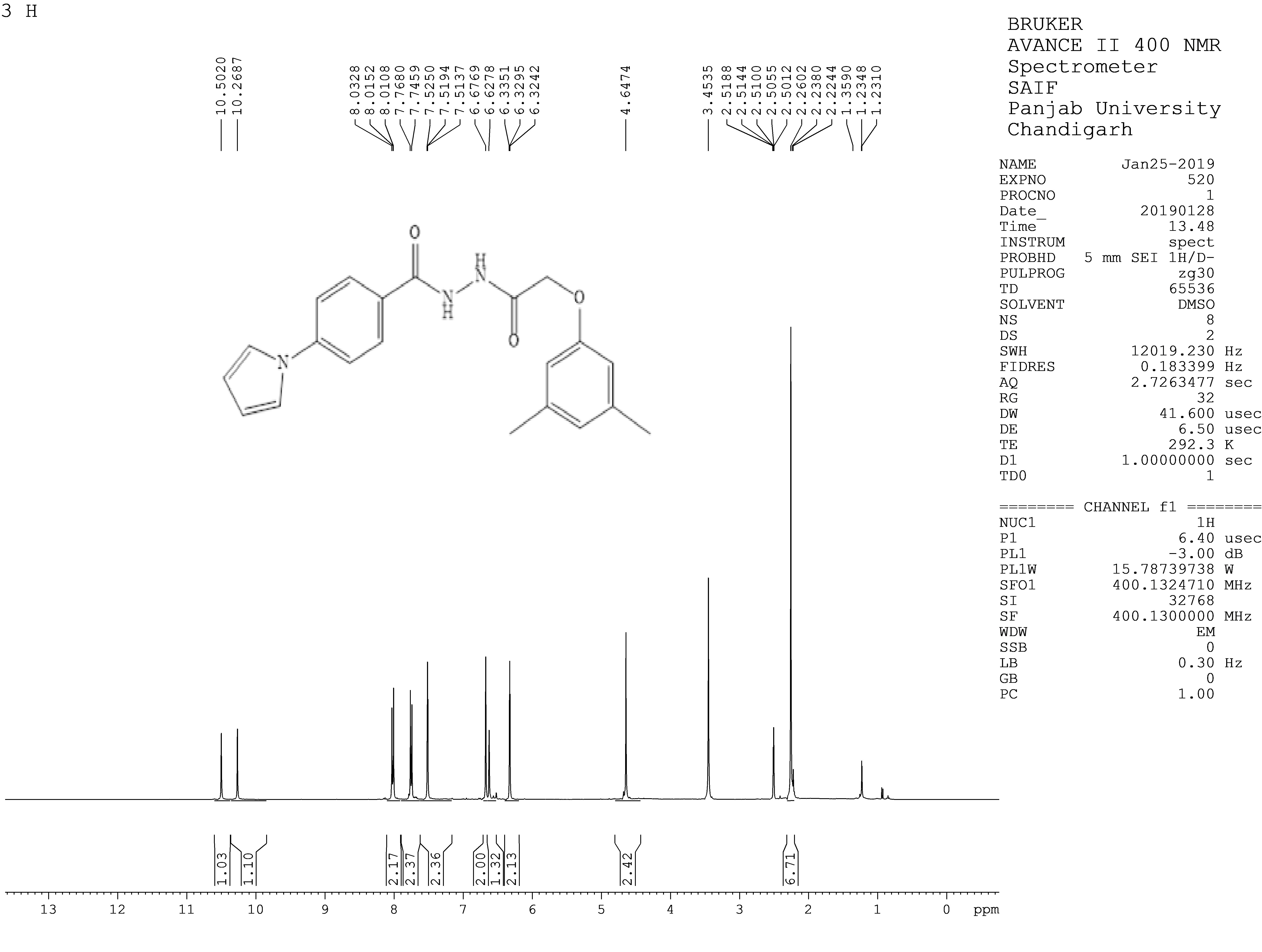


SPECTRUM 26: MASS SPECTRUM OF COMPOUND 3H


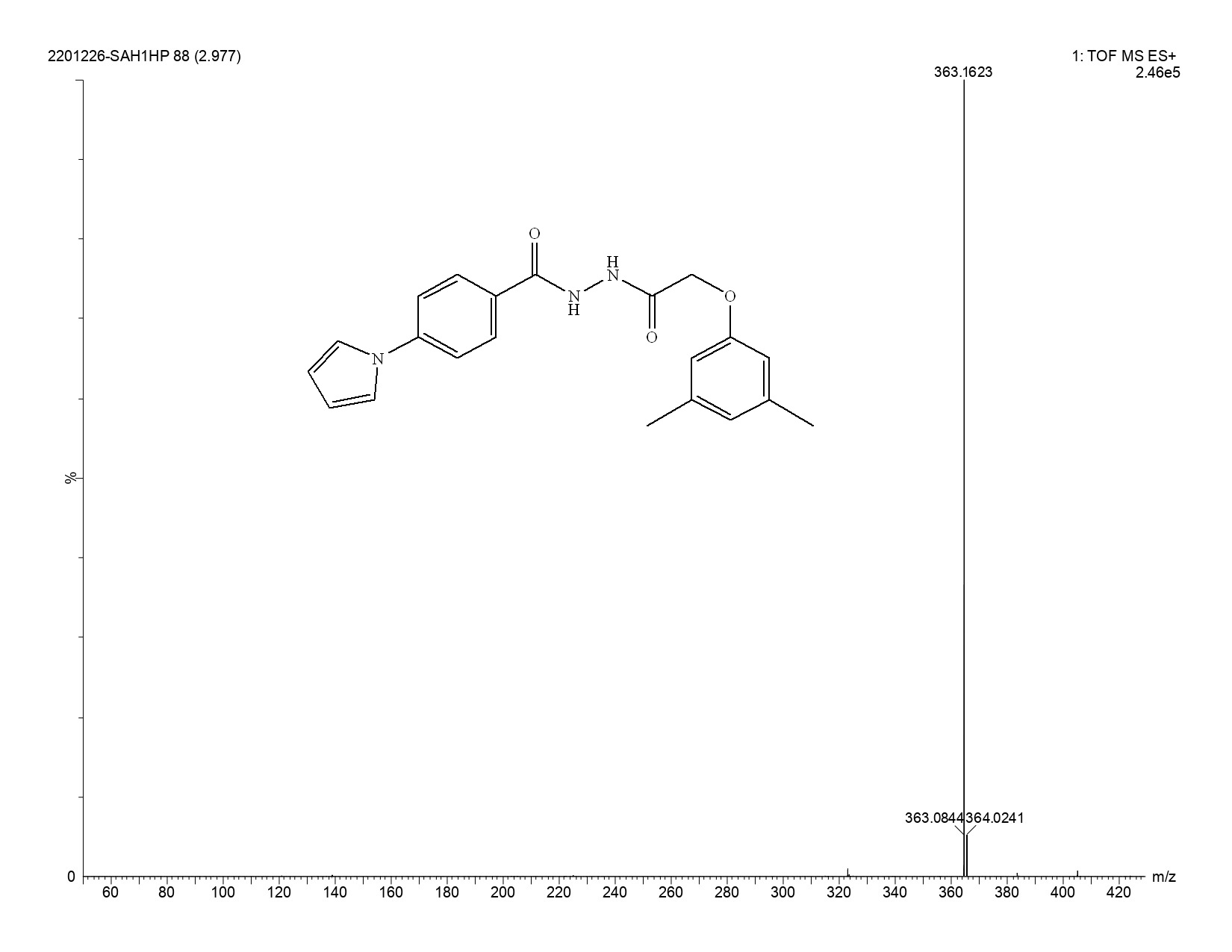


SPECTRUM 27: IR SPECTRUM OF COMPOUND 3I


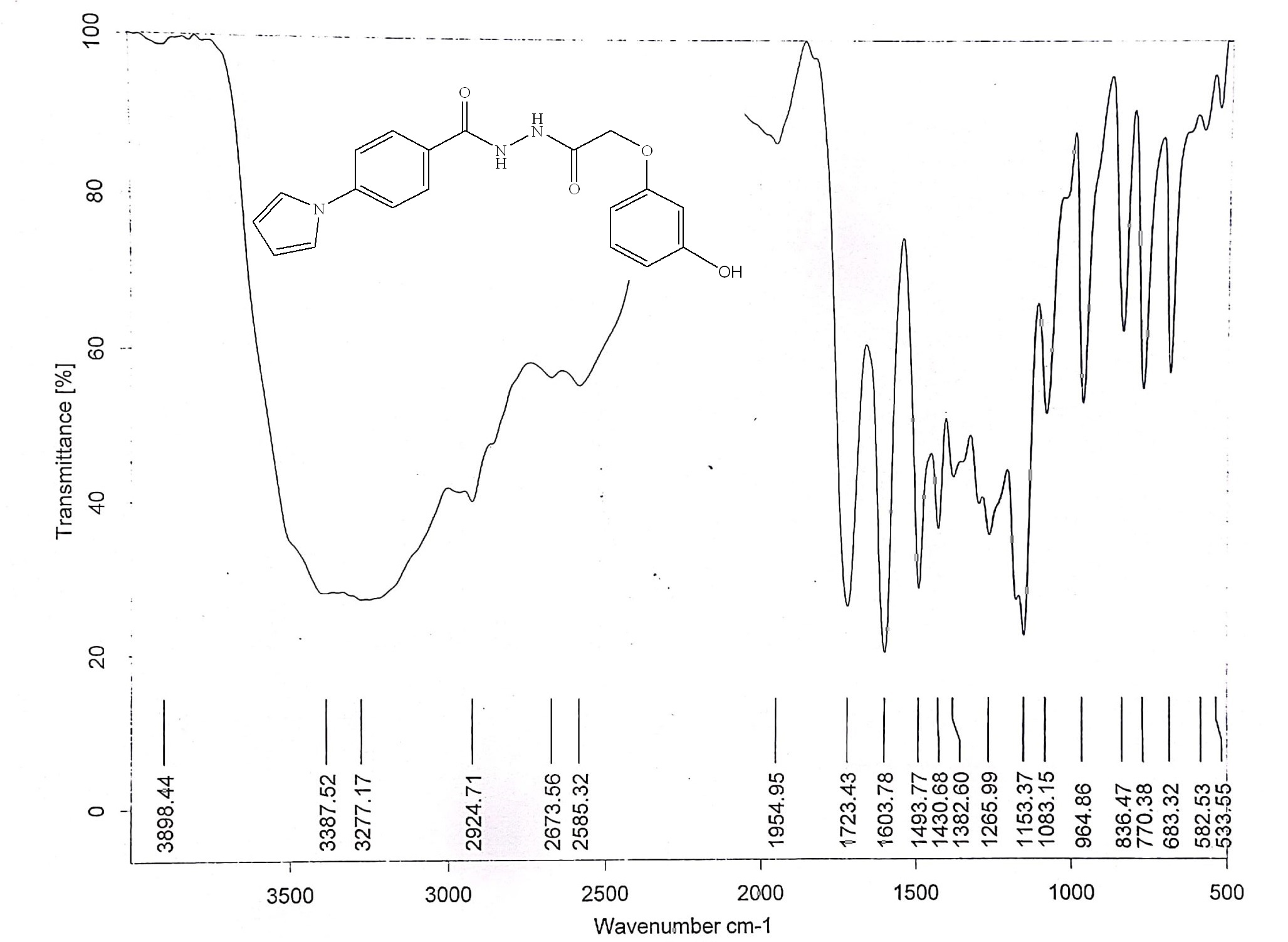


SPECTRUM 28: ^1^HNMR SPECTRUM OF COMPOUND 3I


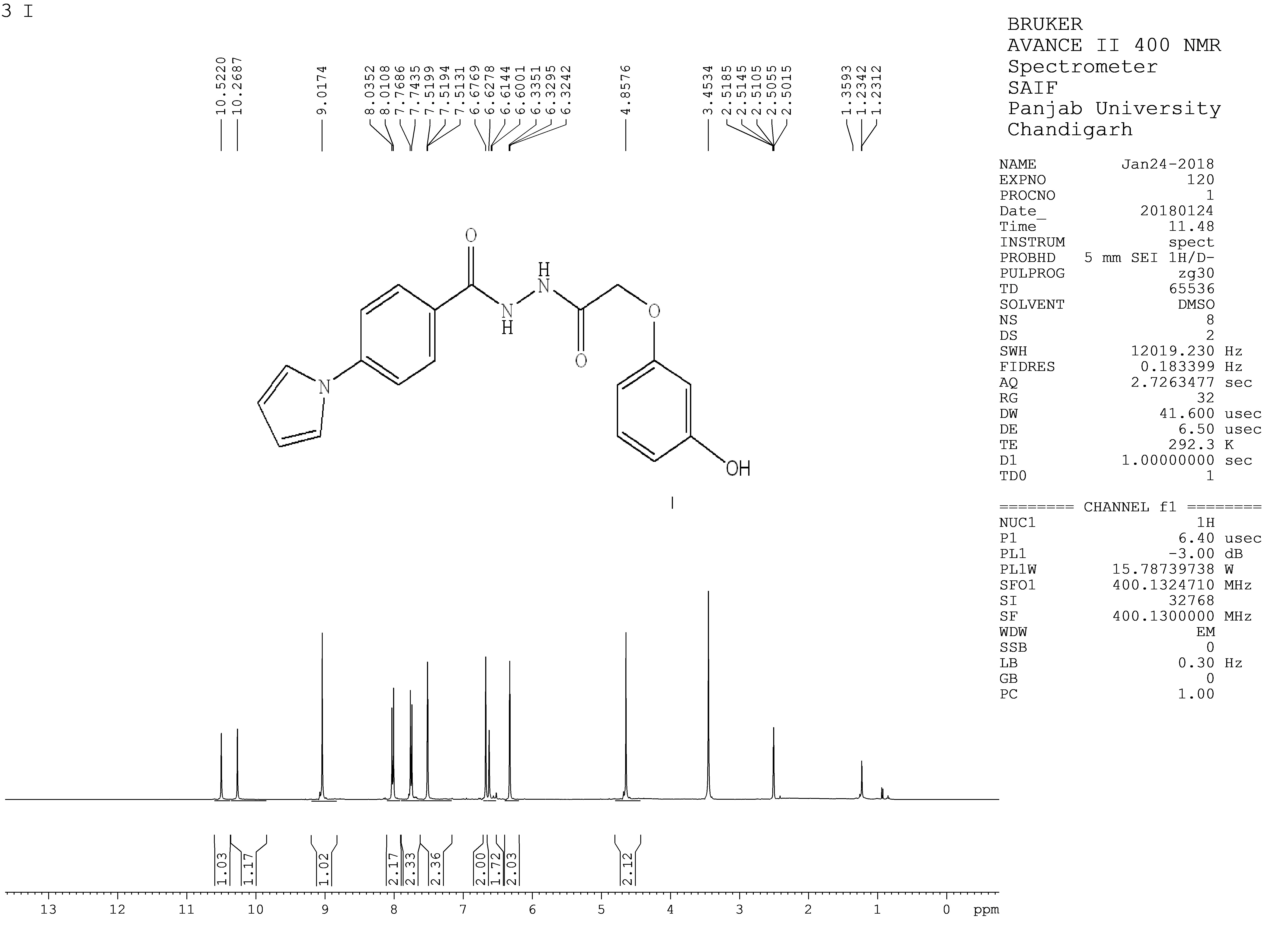


SPECTRUM 29: MASS SPECTRUM OF COMPOUND 3I


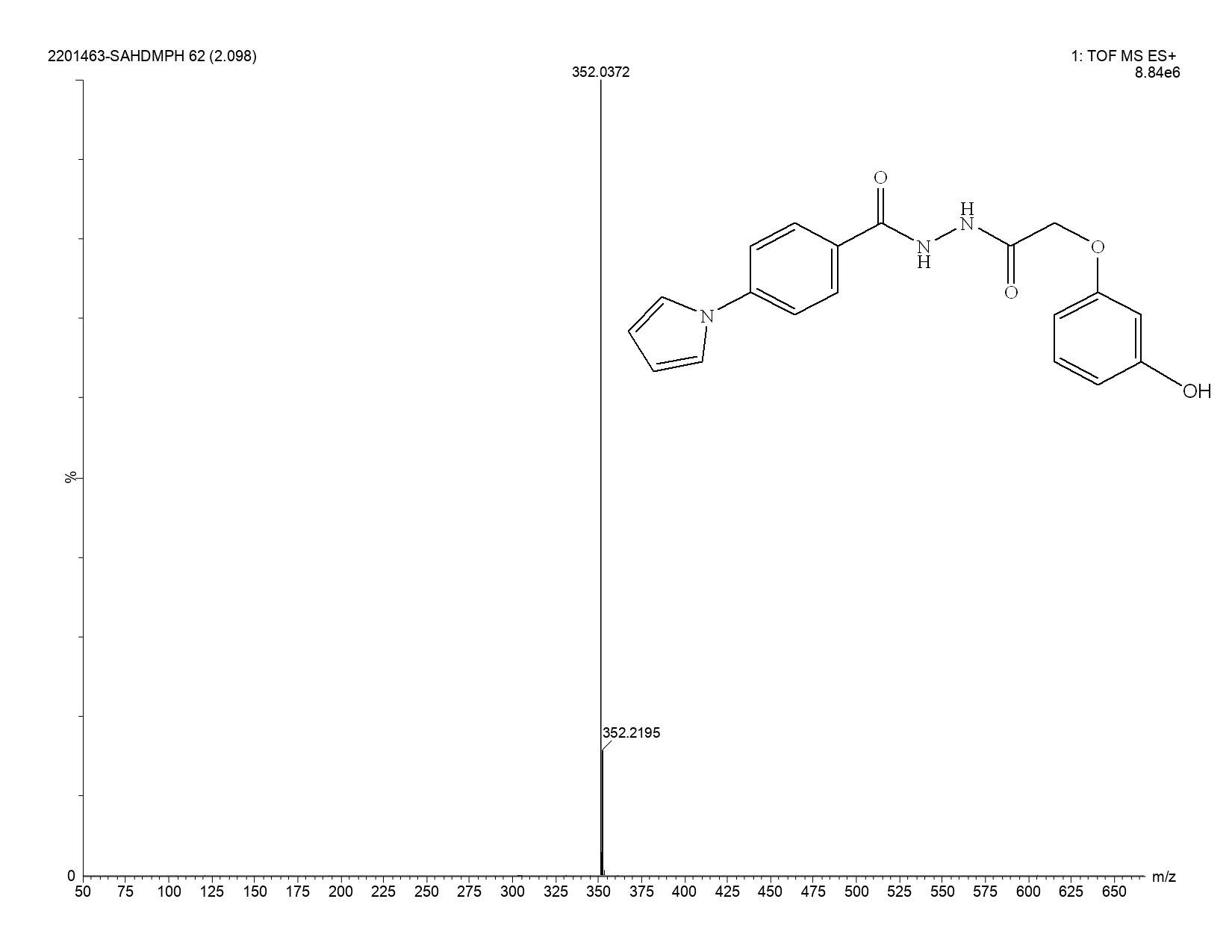


SPECTRUM 30: IR SPECTRUM OF COMPOUND 3J


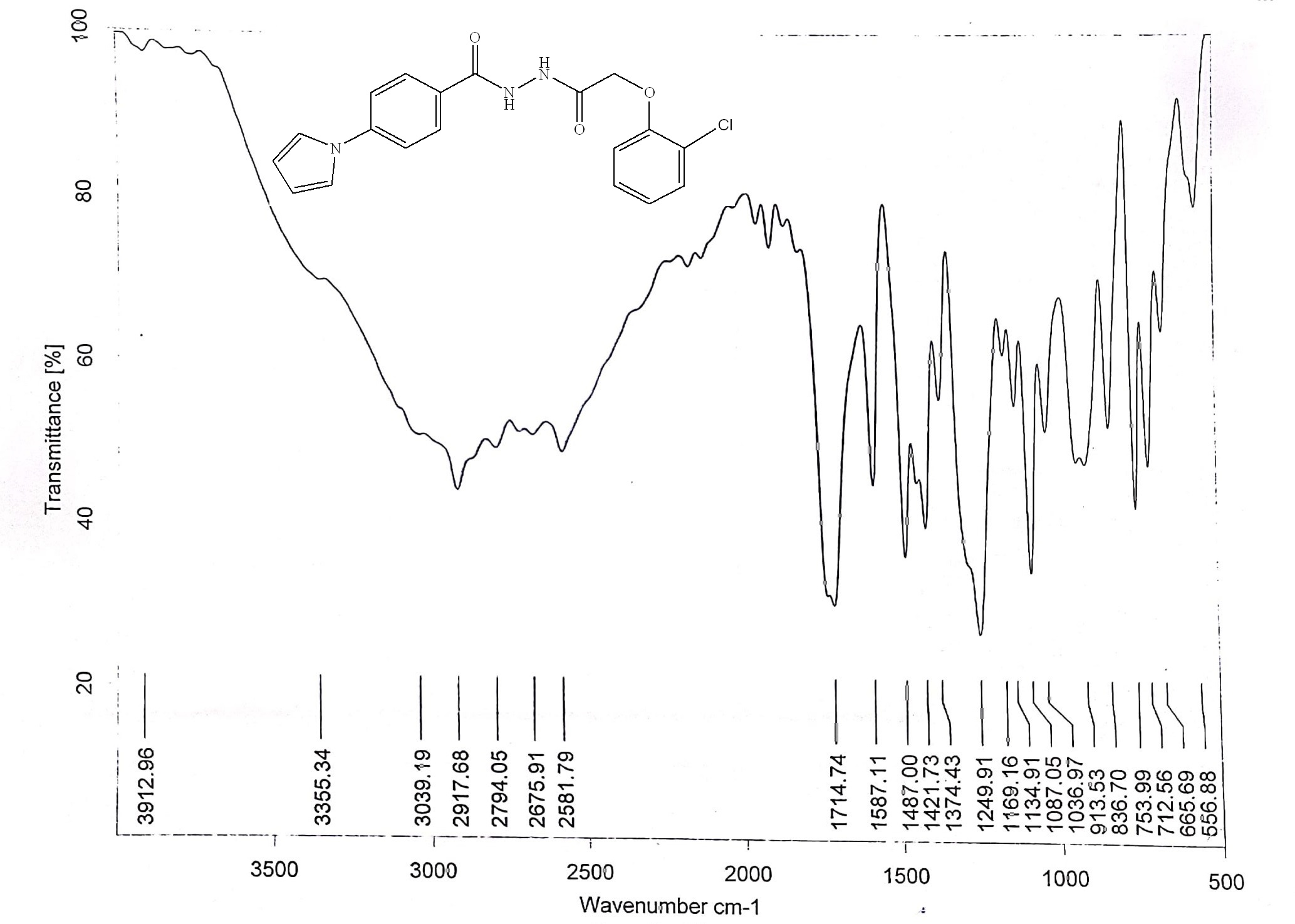


SPECTRUM 31: ^1^HNMR SPECTRUM OF COMPOUND 3J


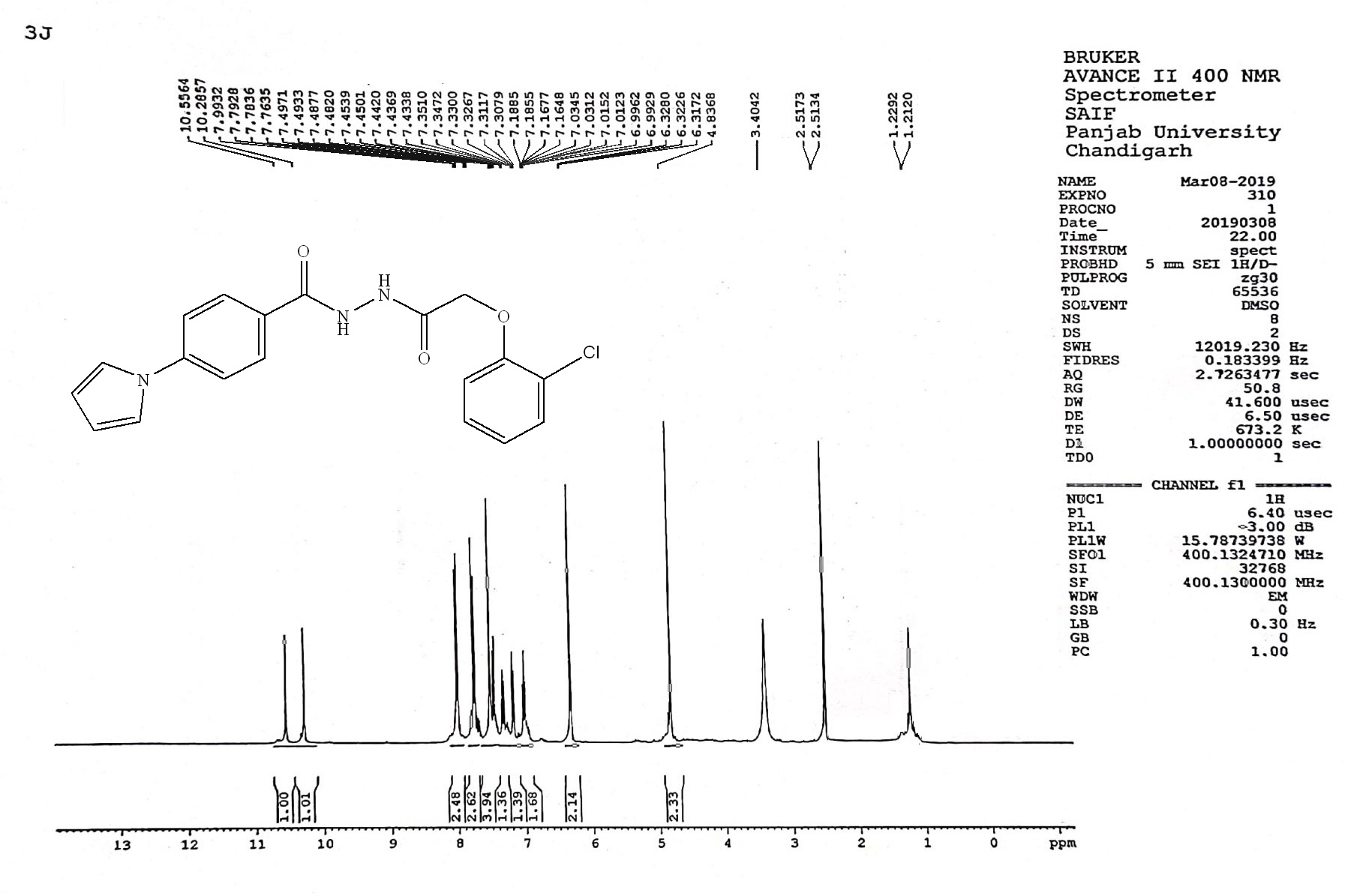


SPECTRUM 32: MASS SPECTRUM OF COMPOUND 3J


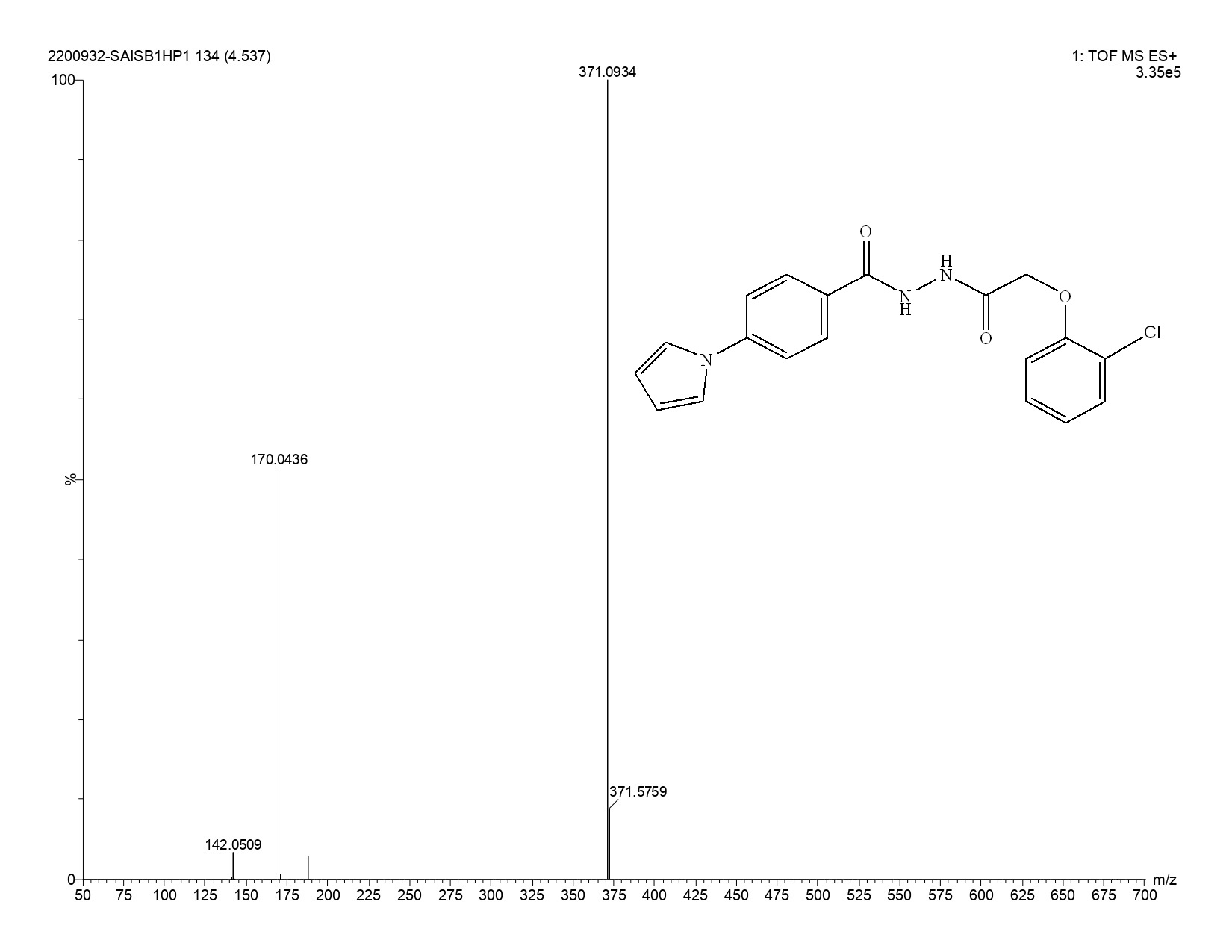


SPECTRUM 33: IR SPECTRUM OF COMPOUND 5A


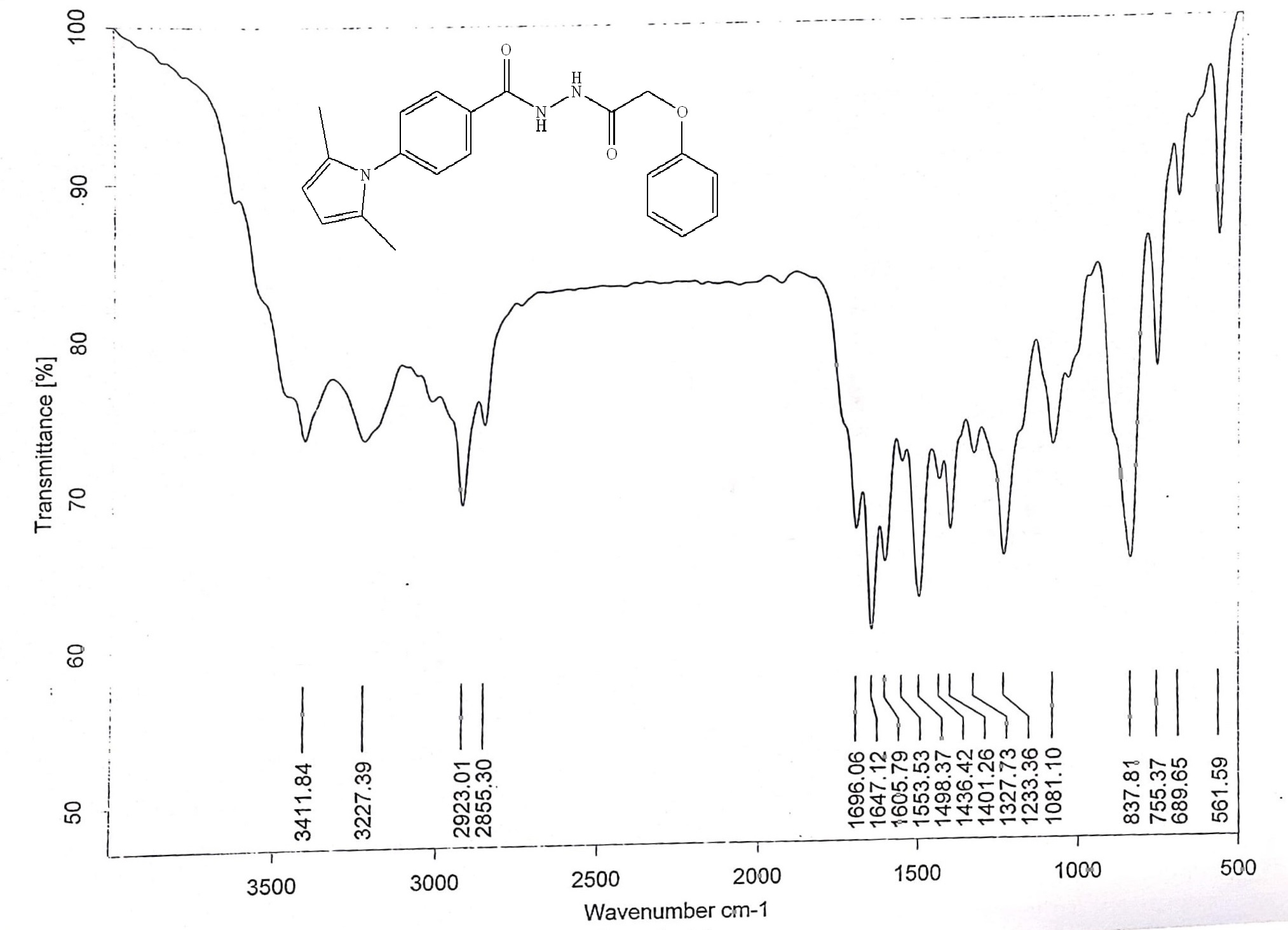


SPECTRUM 34: ^1^HNMR SPECTRUM OF COMPOUND 5A


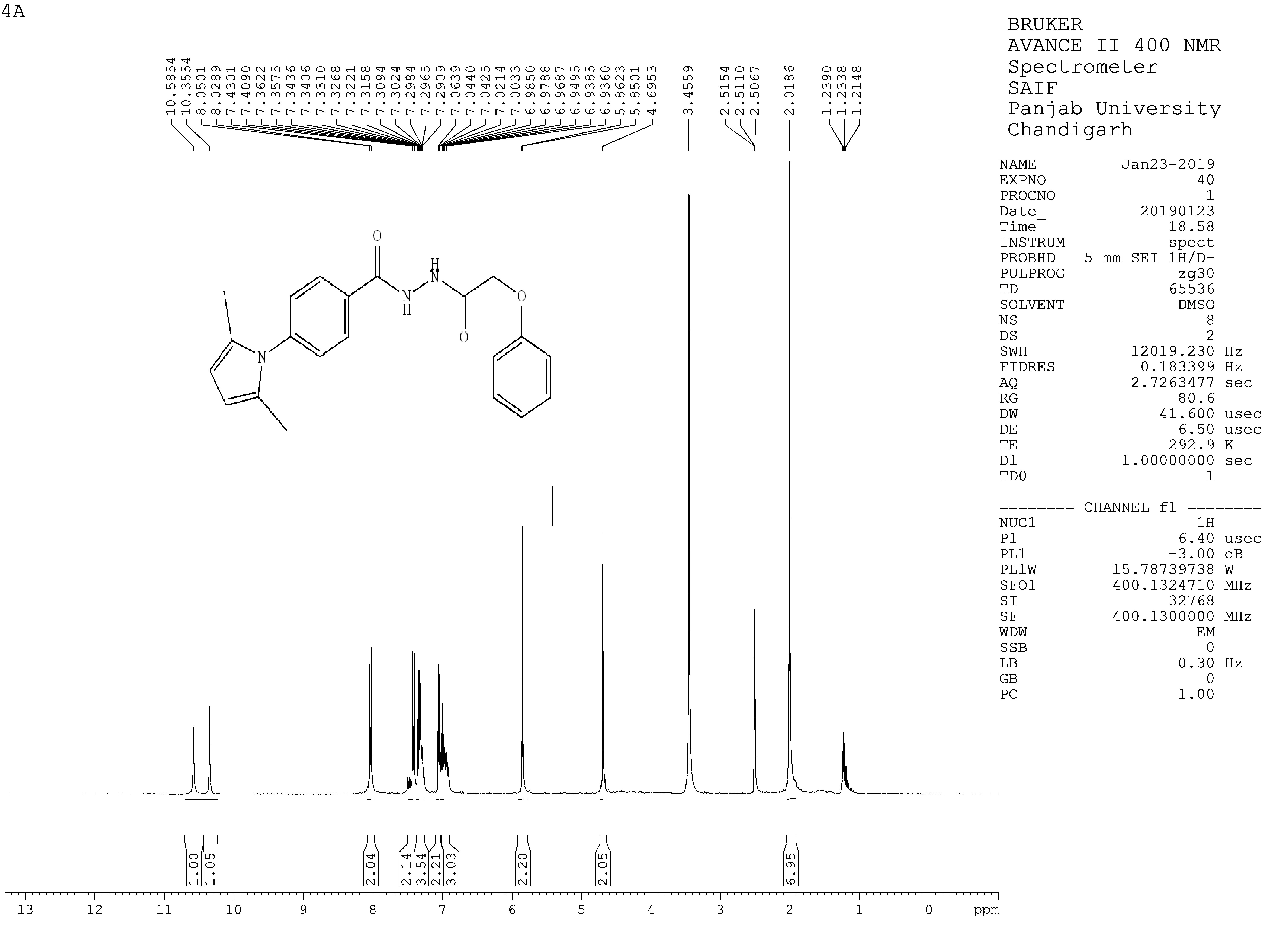


SPECTRUM 35: MASS SPECTRUM OF COMPOUND 5A


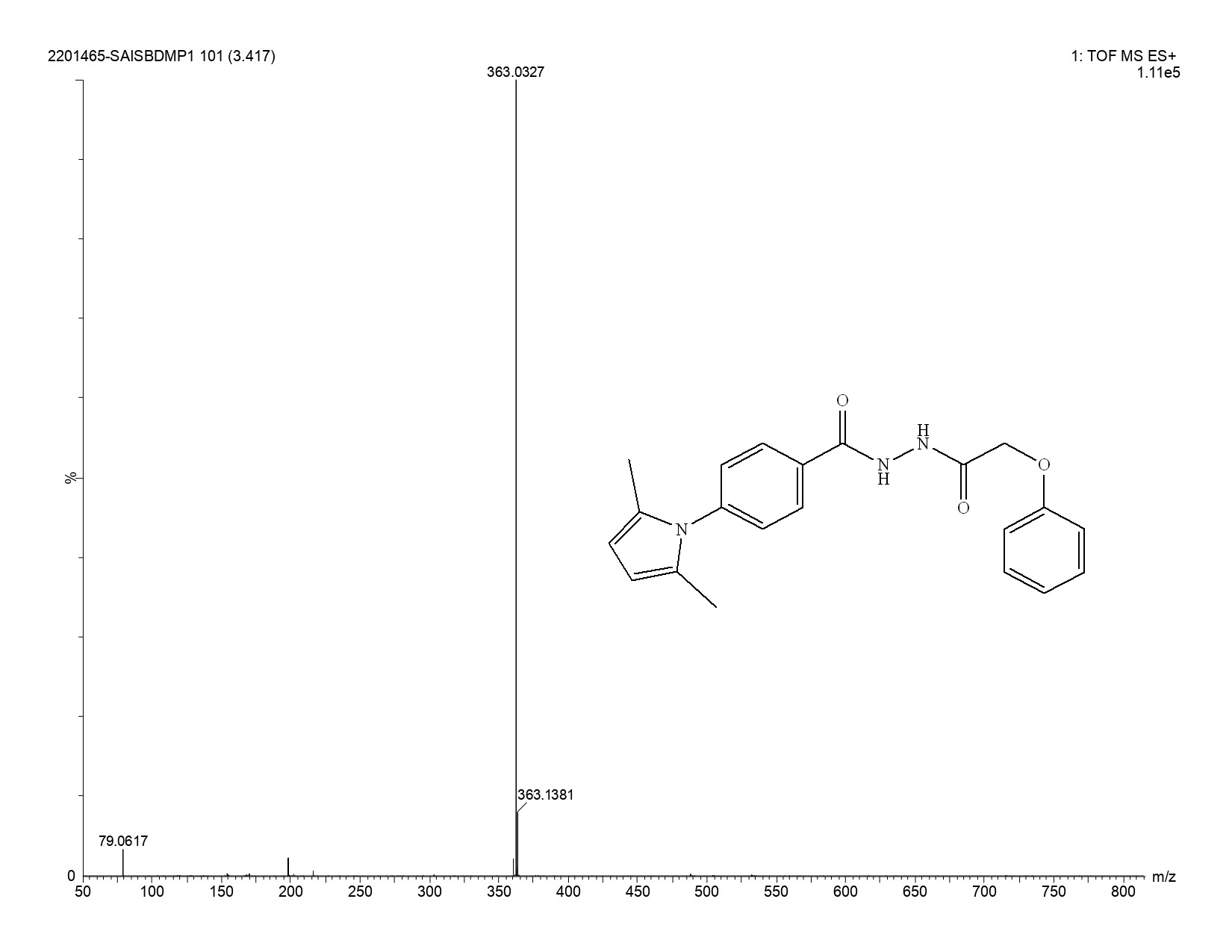


SPECTRUM 36: IR SPECTRUM OF COMPOUND 5B


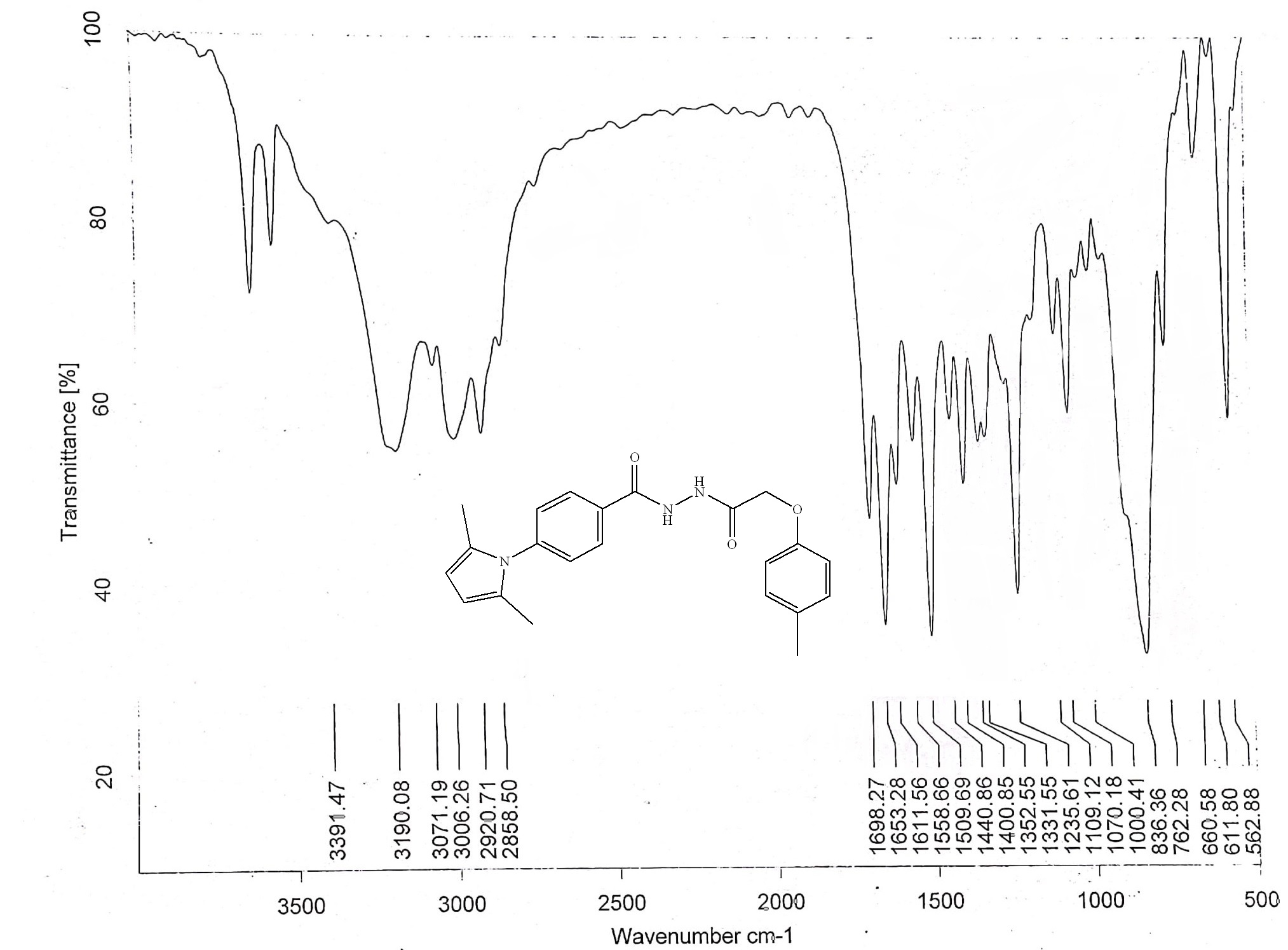


SPECTRUM 37: ^1^HNMR SPECTRUM OF COMPOUND 5B


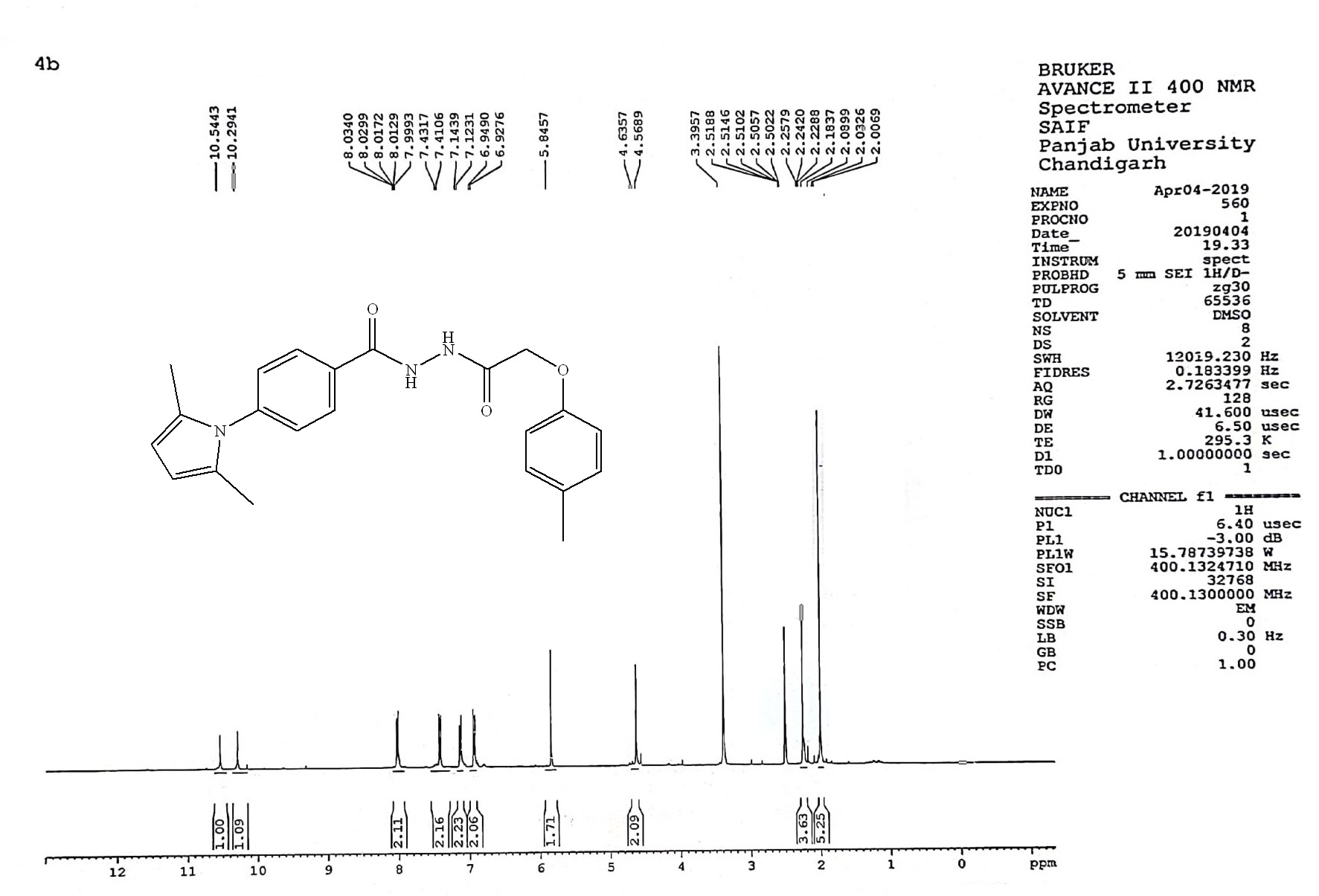


SPECTRUM 38: MASS SPECTRUM OF COMPOUND 5B


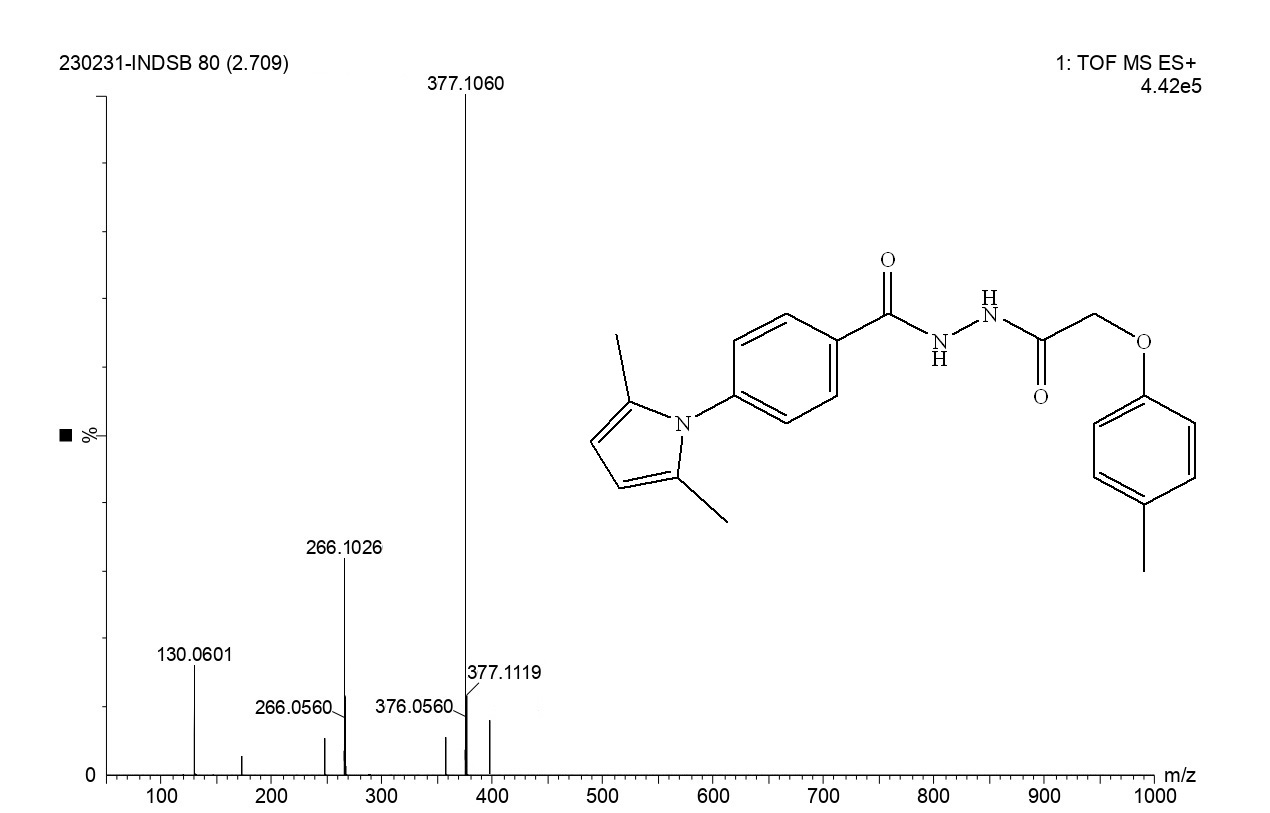


SPECTRUM 39: IR SPECTRUM OF COMPOUND 5C


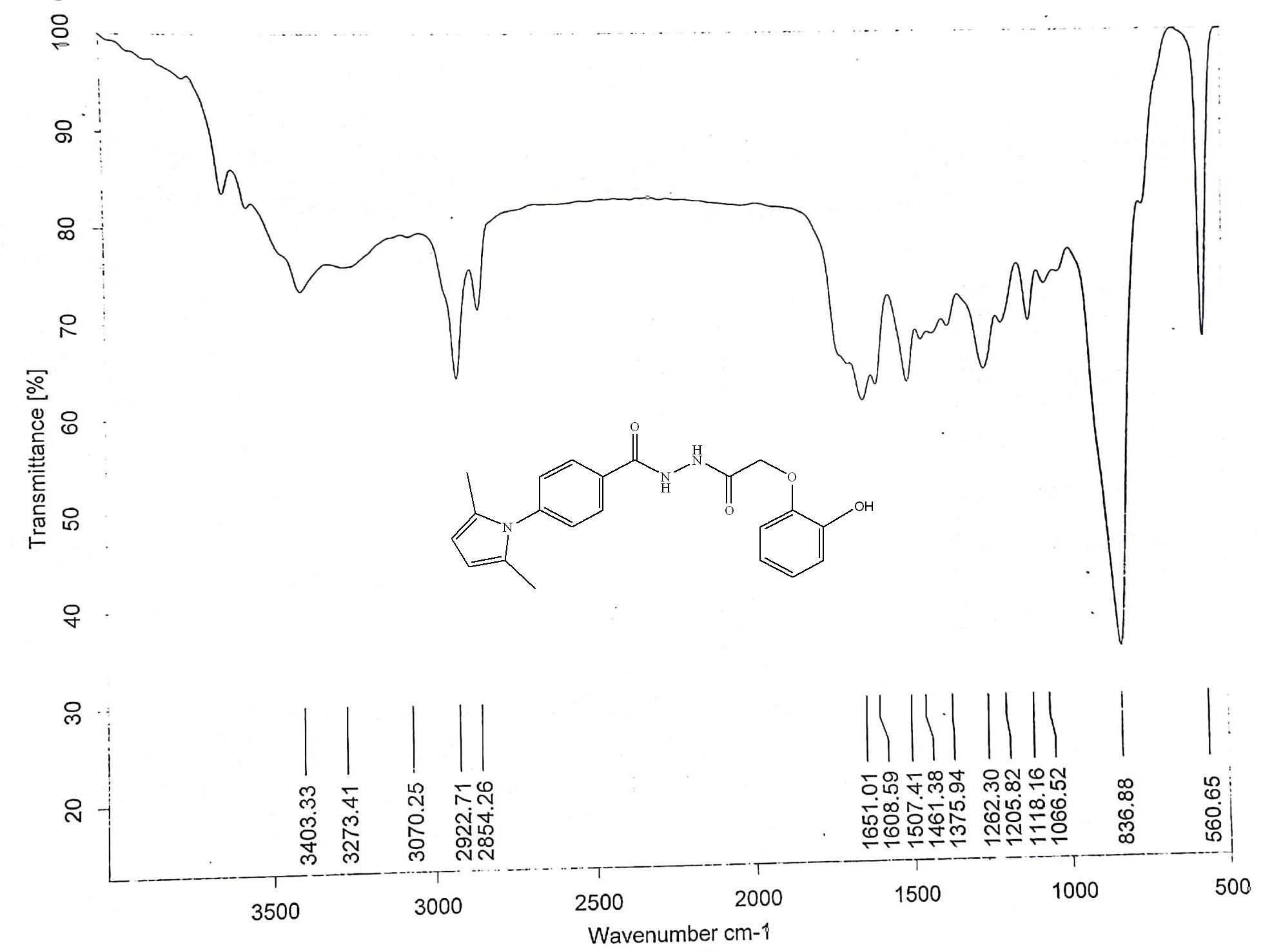


SPECTRUM 40: ^1^HNMR SPECTRUM OF COMPOUND 5C


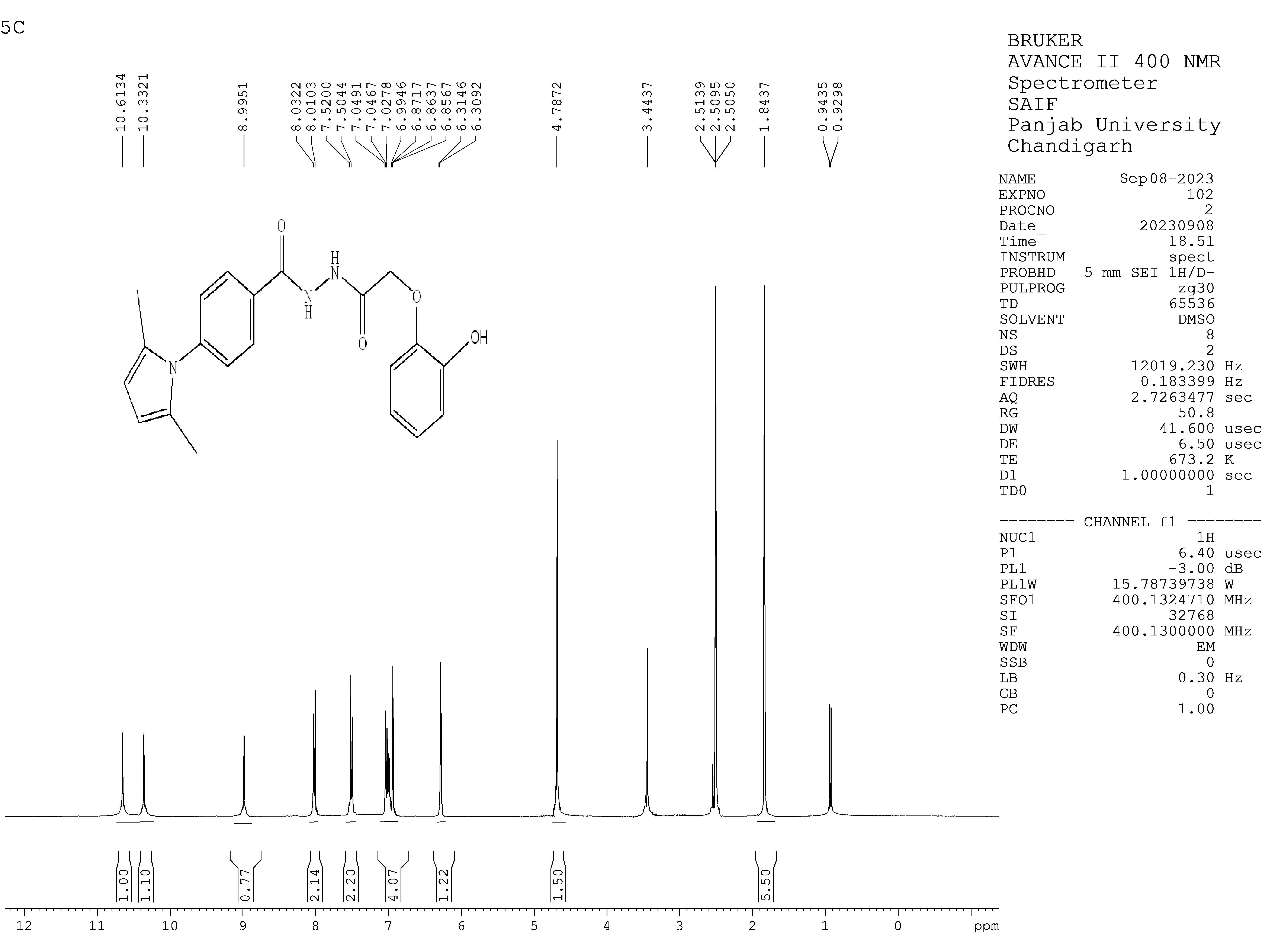


SPECTRUM 41: ^13^ C NMR SPECTRUM OF COMPOUND 5C


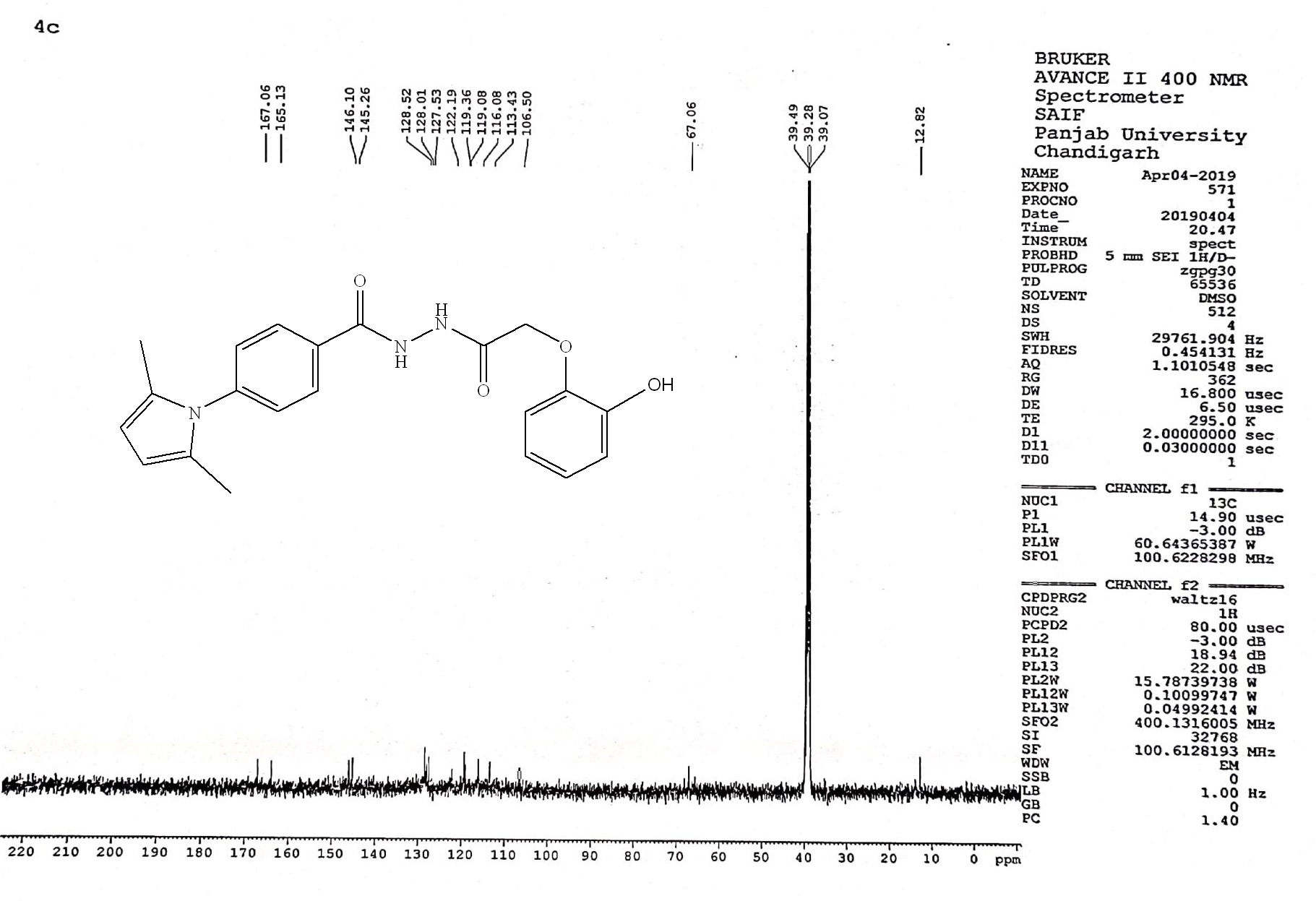


SPECTRUM 42: MASS SPECTRUM OF COMPOUND 5C


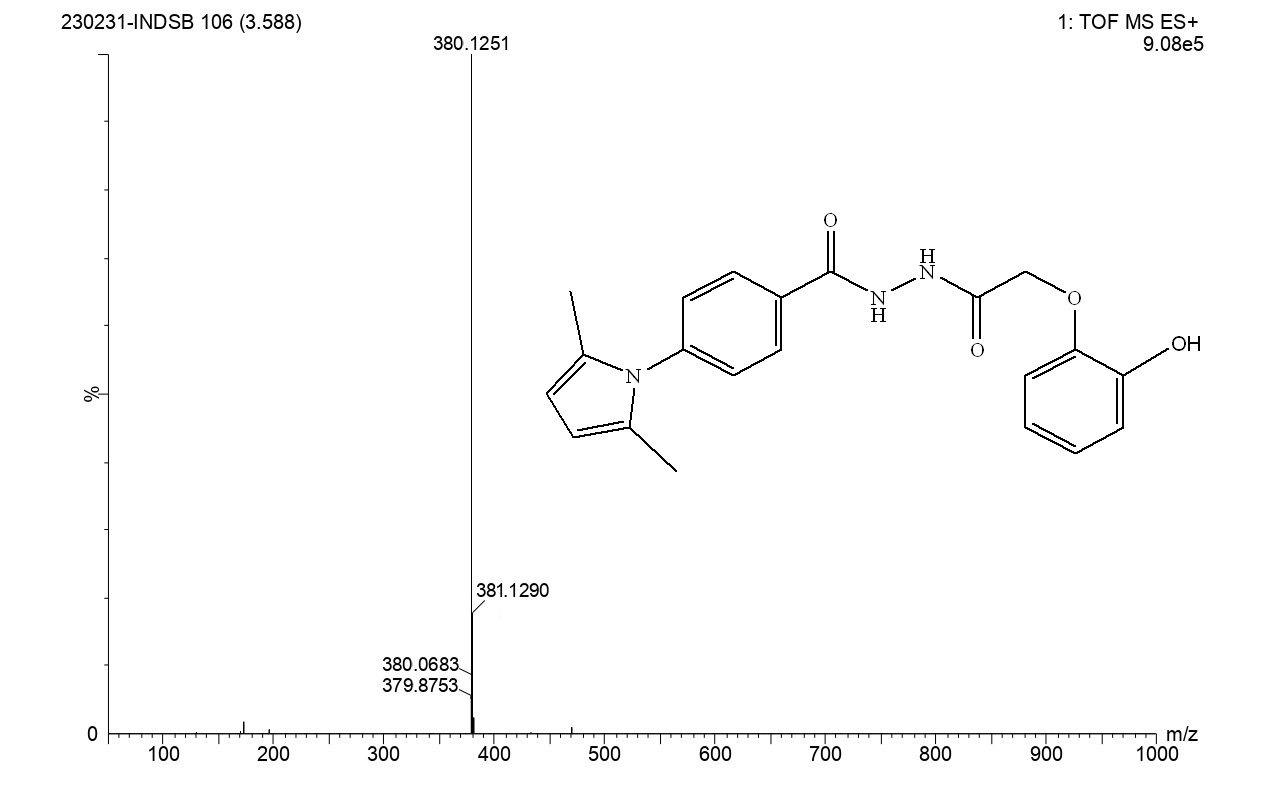


SPECTRUM 43: IR SPECTRUM OF COMPOUND 5D


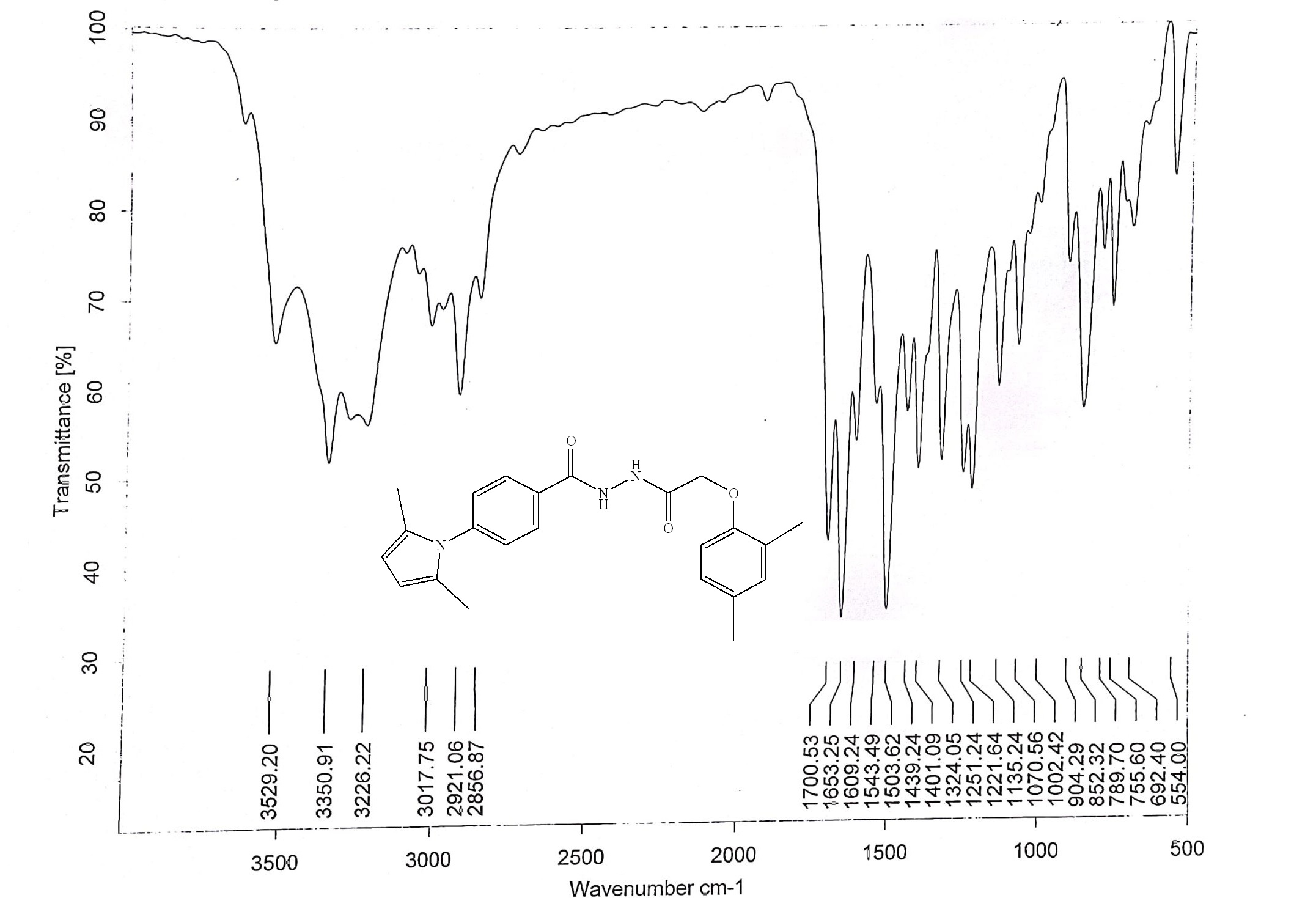


SPECTRUM 44: ^1^HNMR SPECTRUM OF COMPOUND 5D


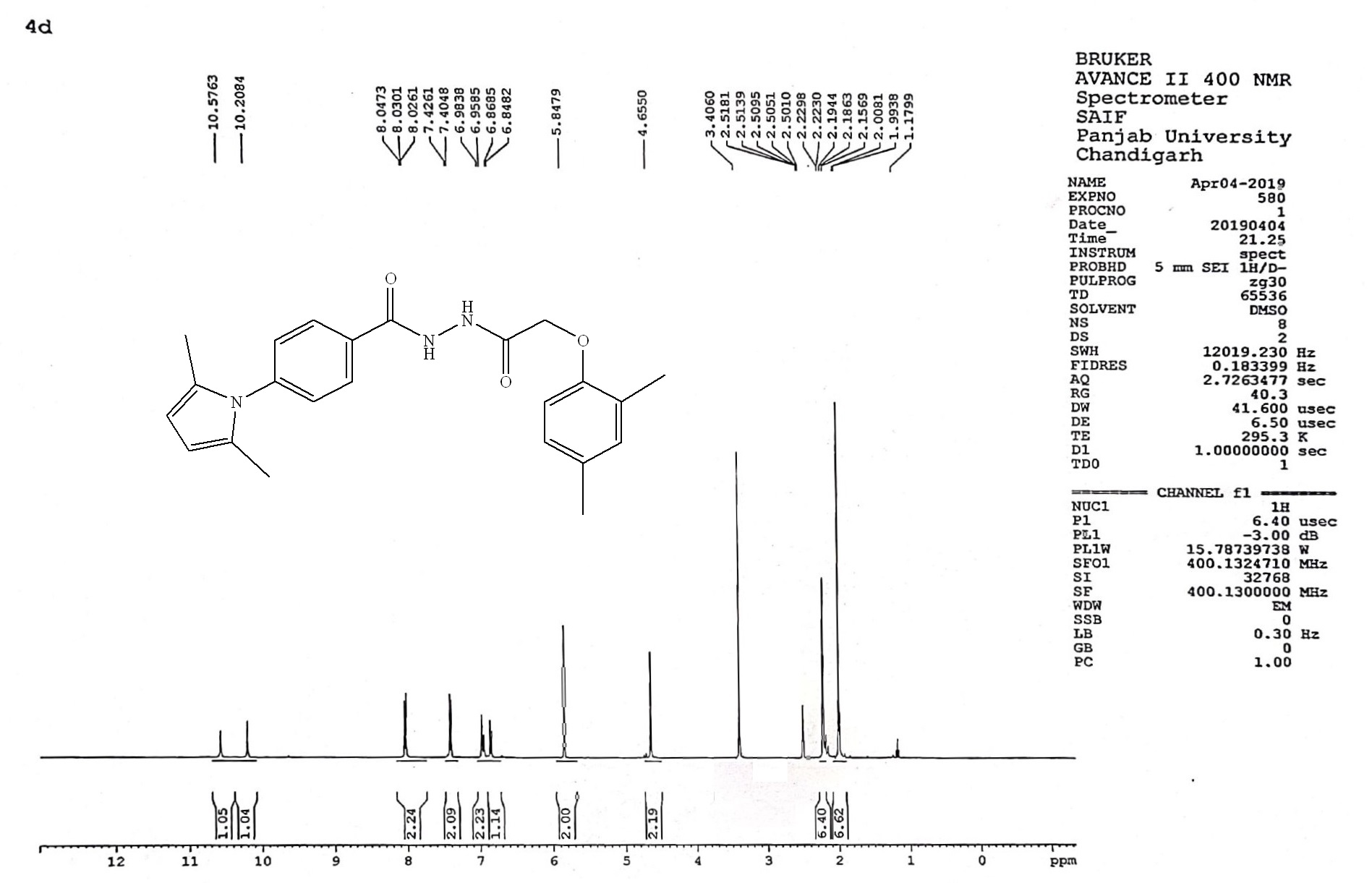


SPECTRUM 45: MASS SPECTRUM OF COMPOUND 5D


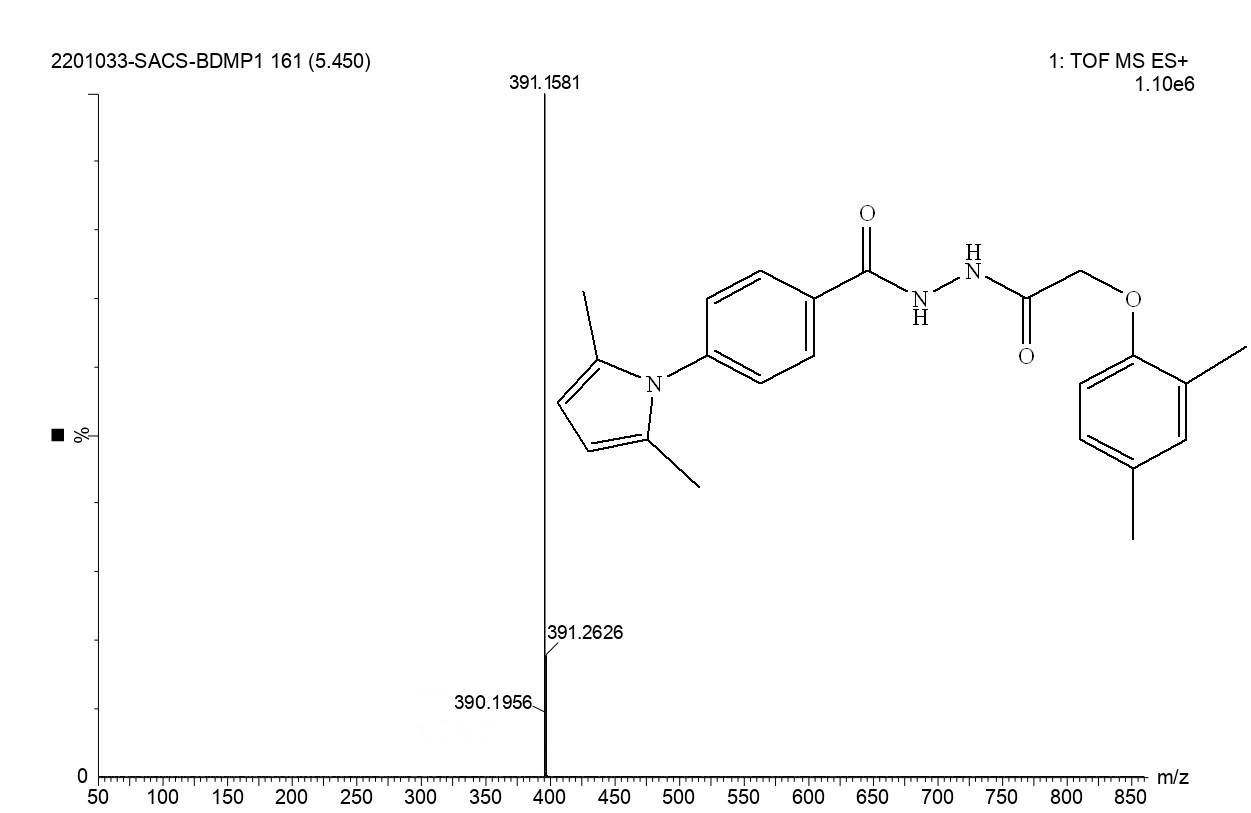


SPECTRUM 46: IR SPECTRUM OF COMPOUND 5E


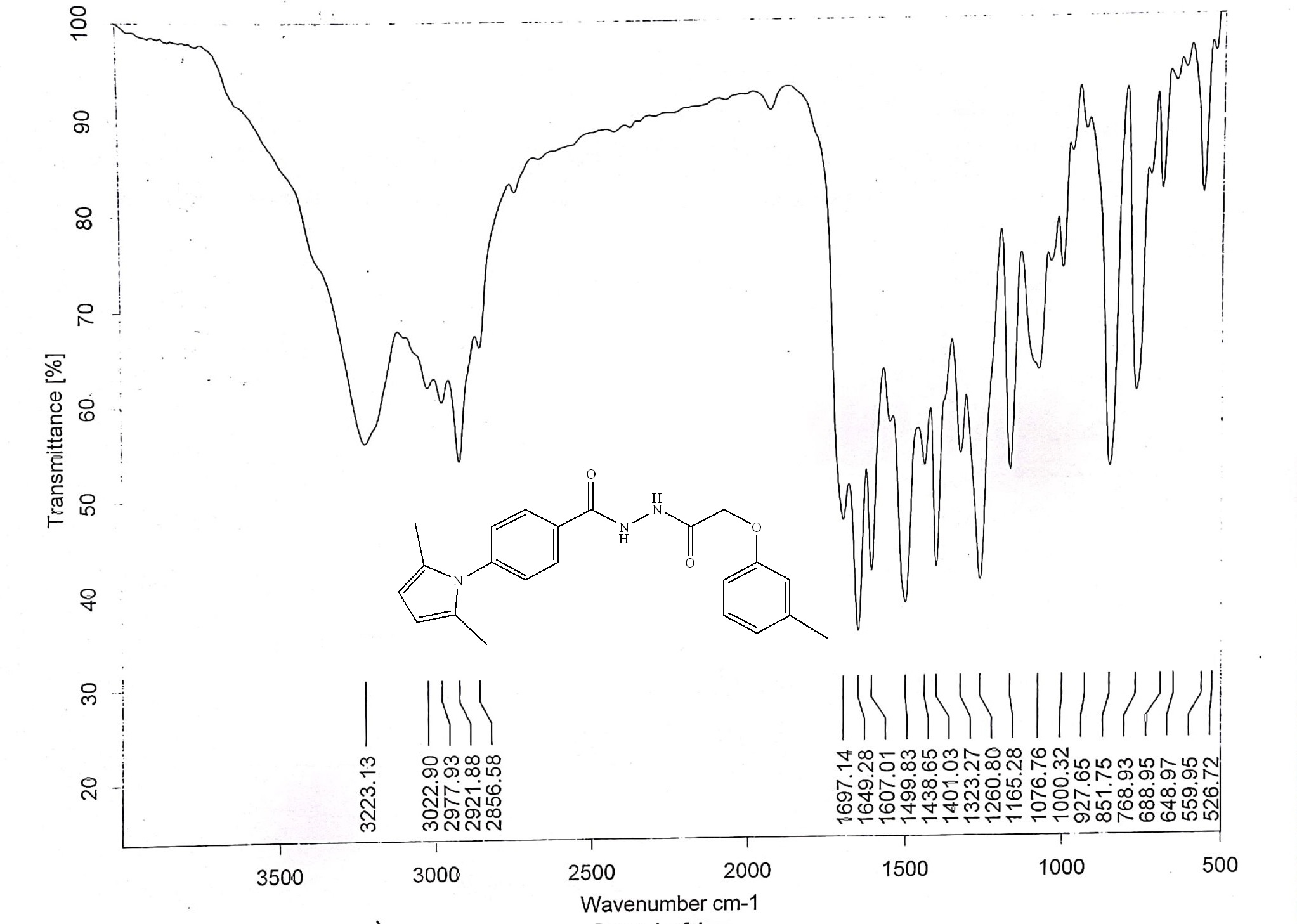


SPECTRUM 47: ^1^HNMR SPECTRUM OF COMPOUND 5E


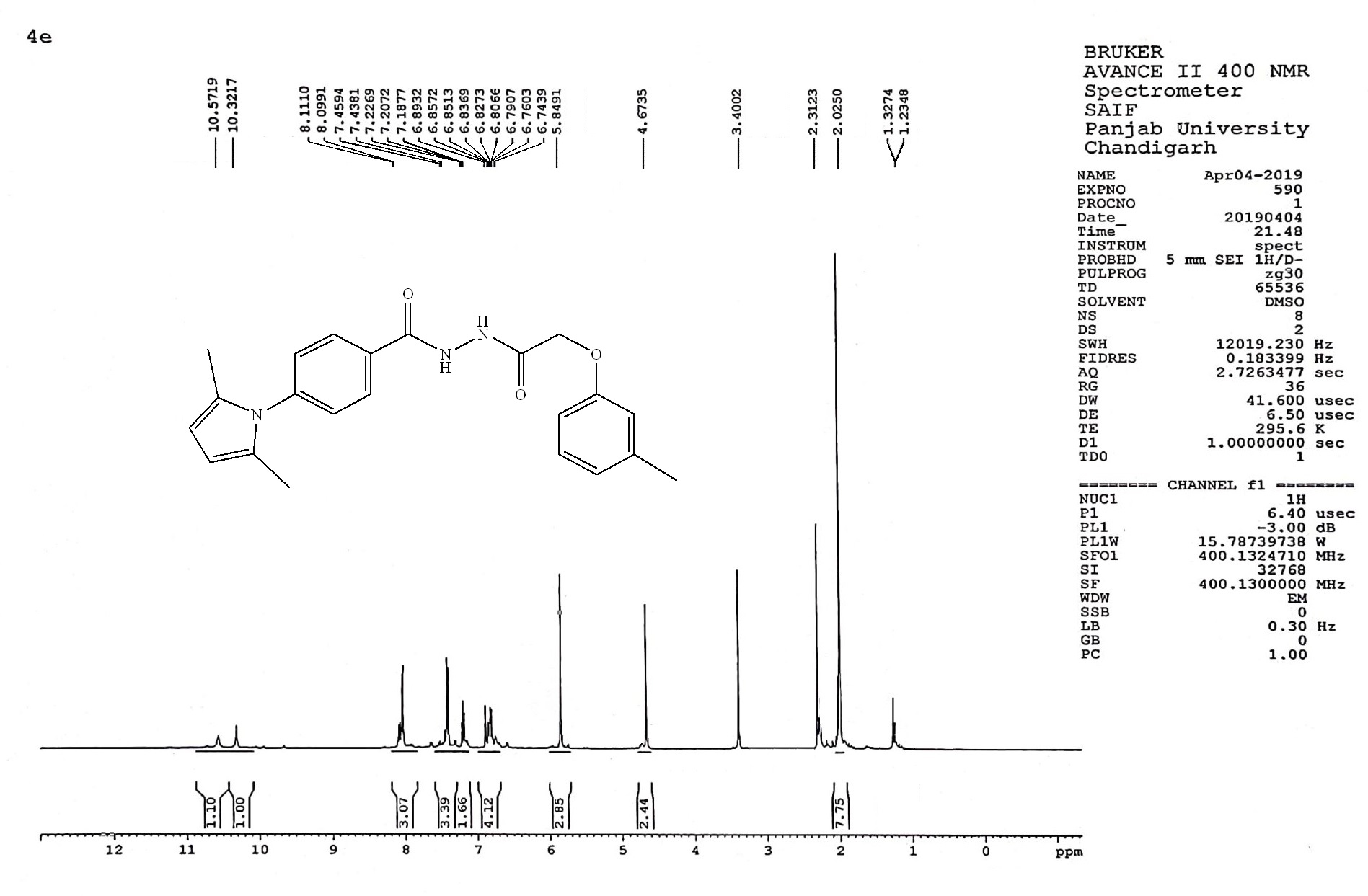


SPECTRUM 48: MASS SPECTRUM OF COMPOUND 5E


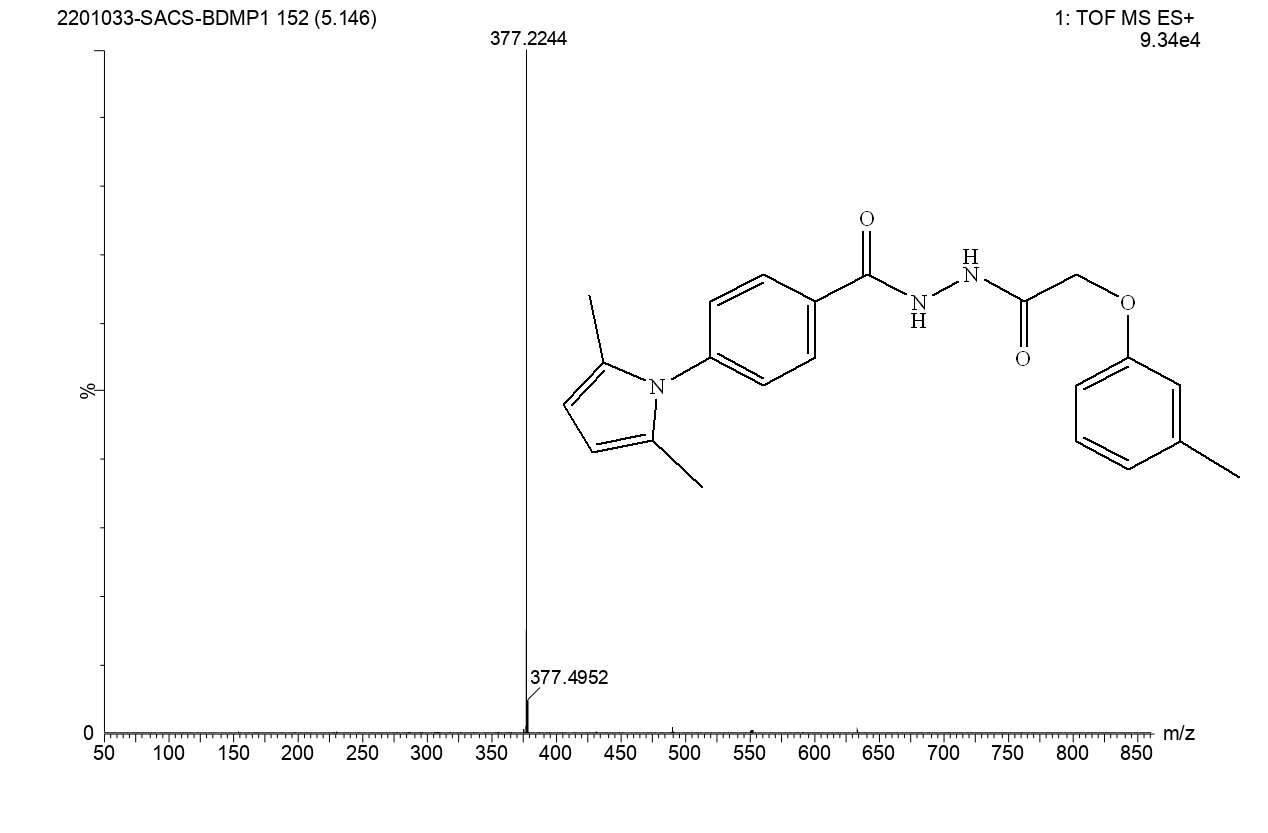


SPECTRUM 49: IR SPECTRUM OF COMPOUND 5F


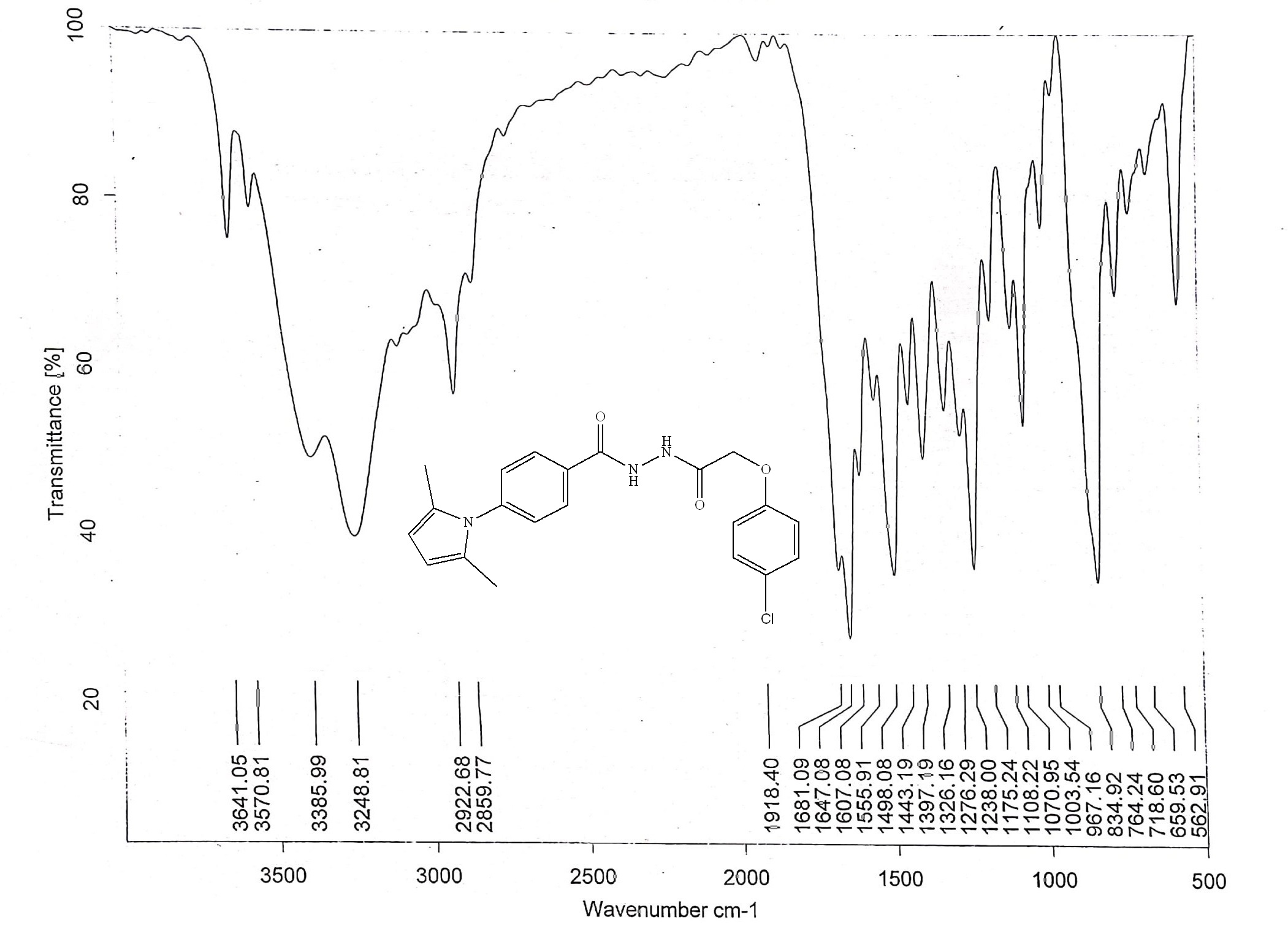


SPECTRUM 50: ^1^HNMR SPECTRUM OF COMPOUND 5F


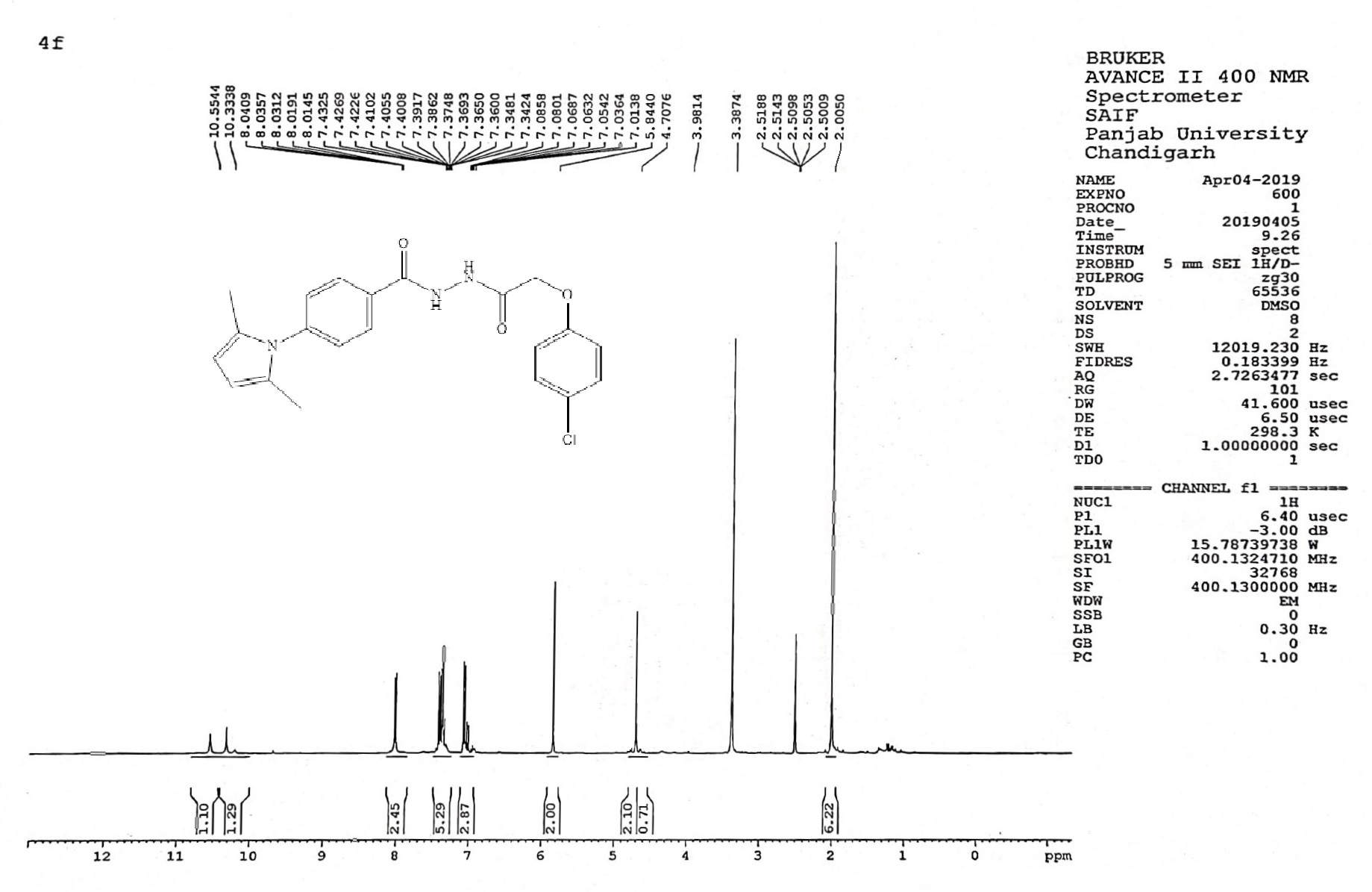


SPECTRUM 51: MASS SPECTRUM OF COMPOUND 5F


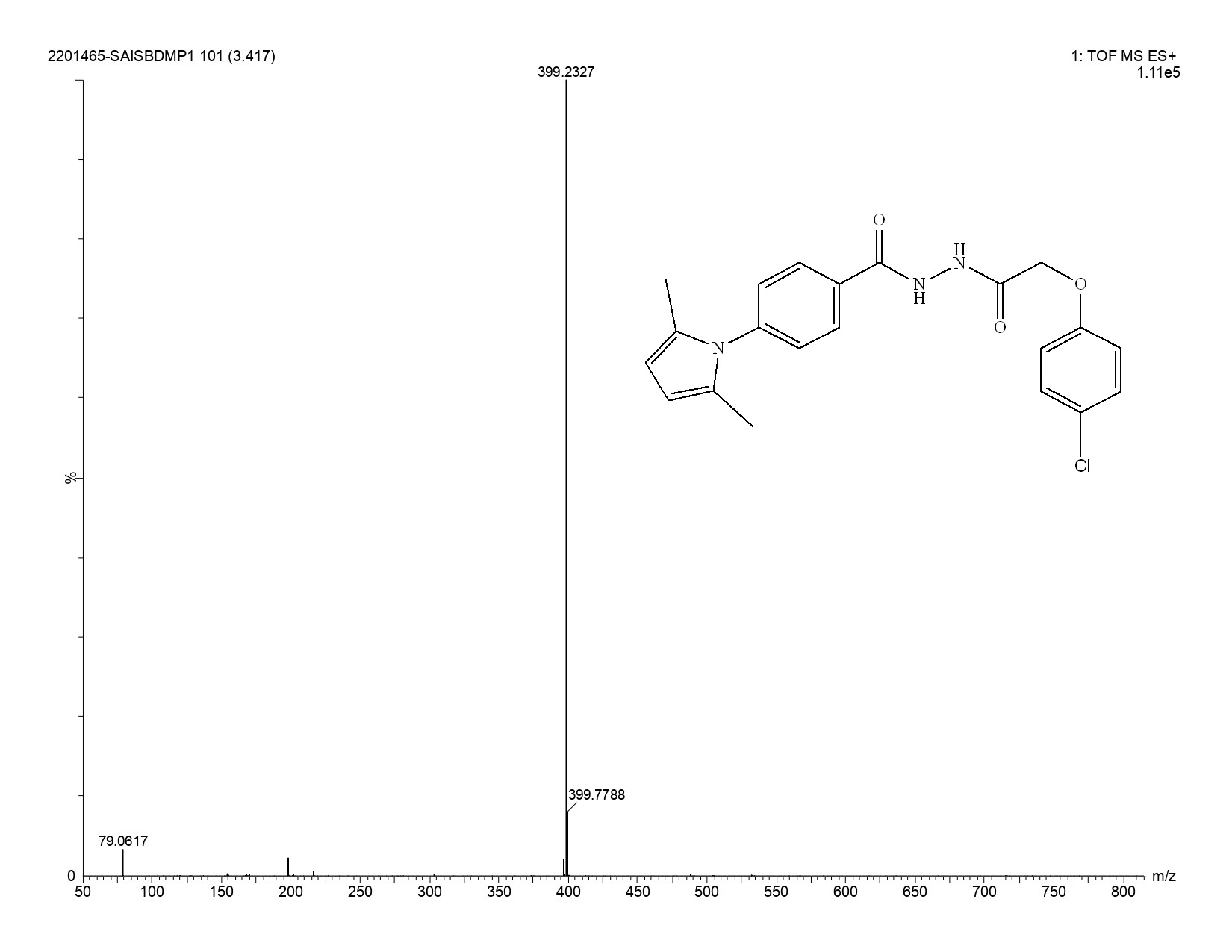


SPECTRUM 52: IR SPECTRUM OF COMPOUND 5G


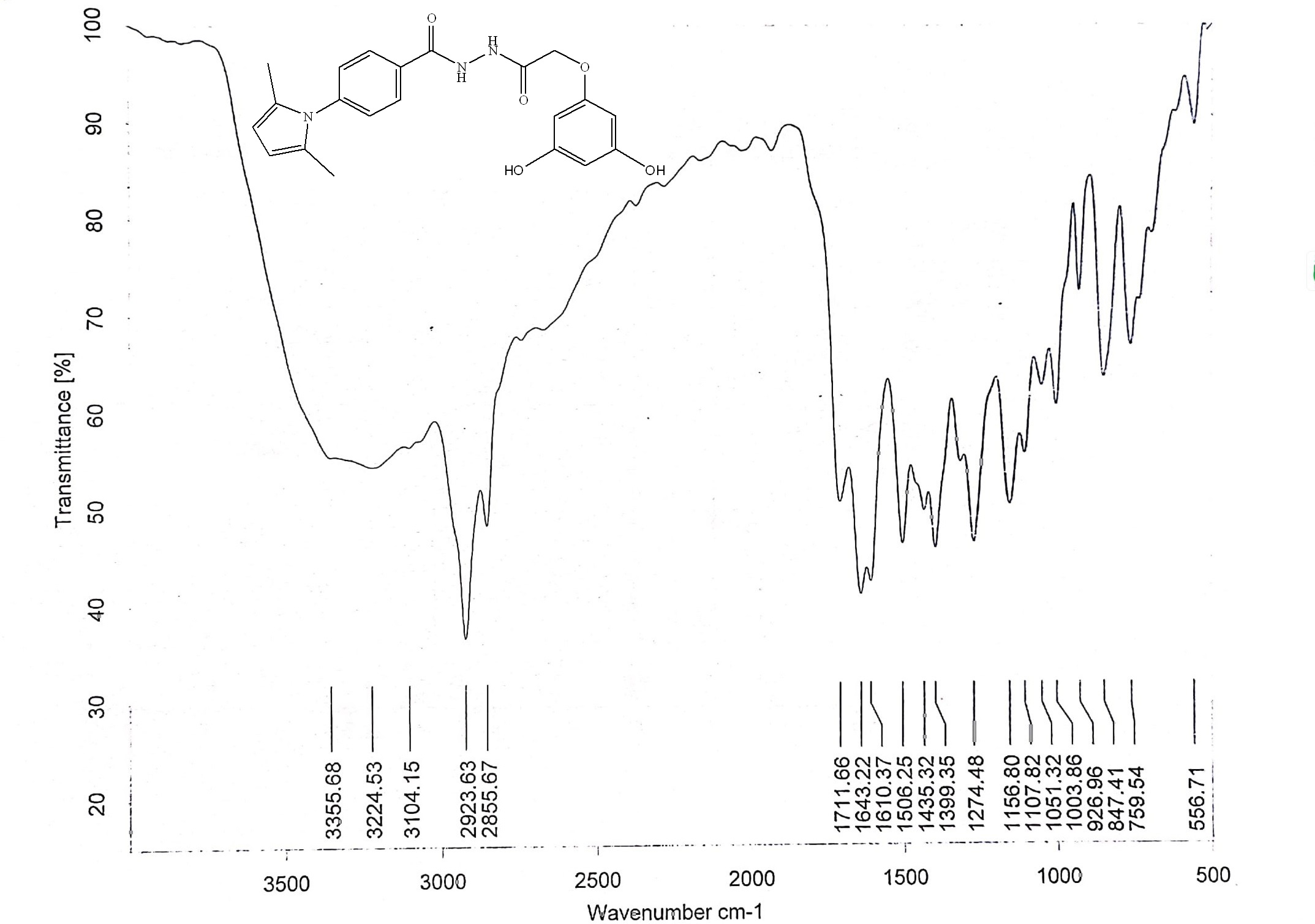


SPECTRUM 53: ^1^HNMR SPECTRUM OF COMPOUND 5G


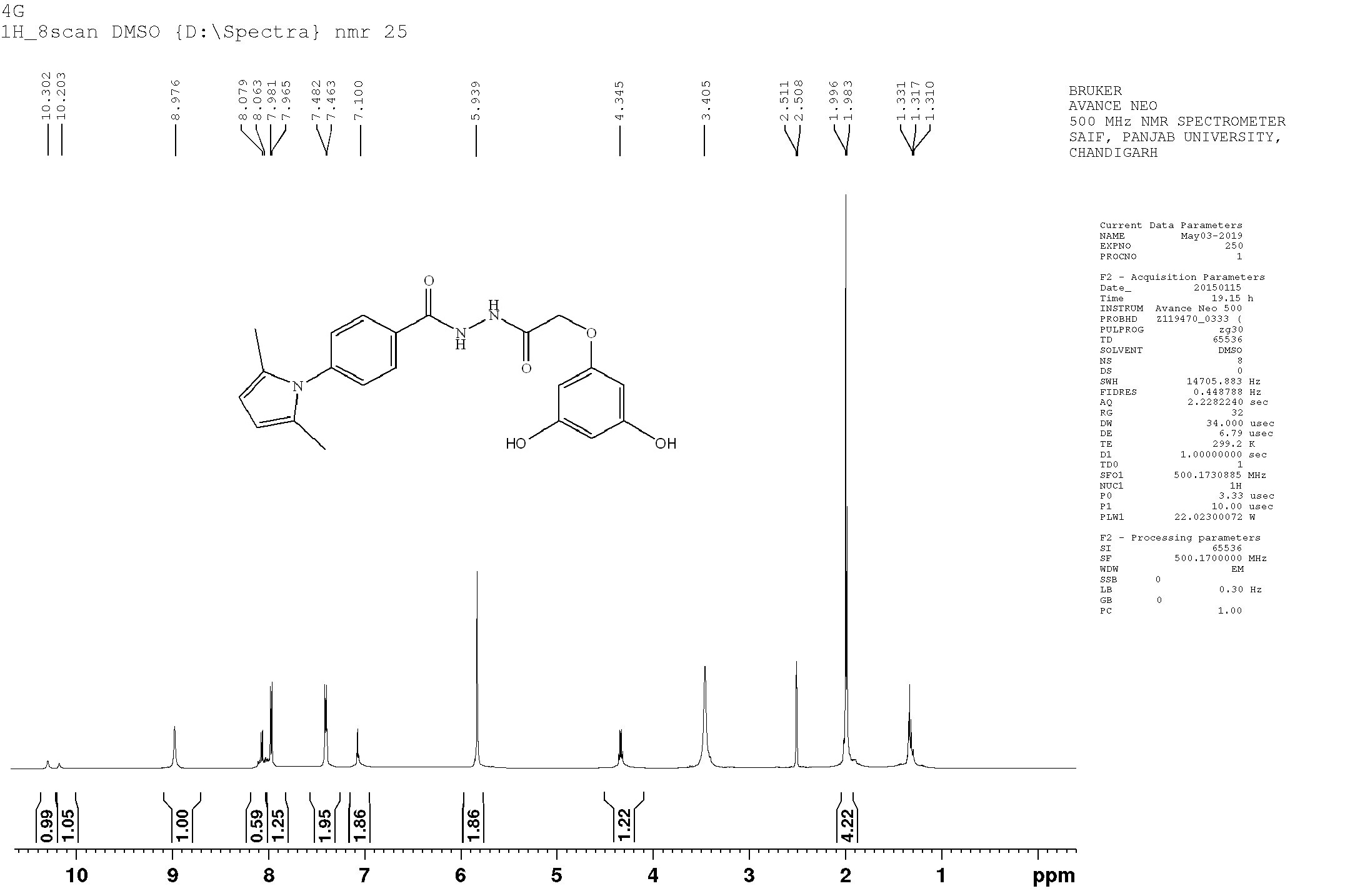


SPECTRUM 54: ^13^ CNMR SPECTRUM OF COMPOUND 5G


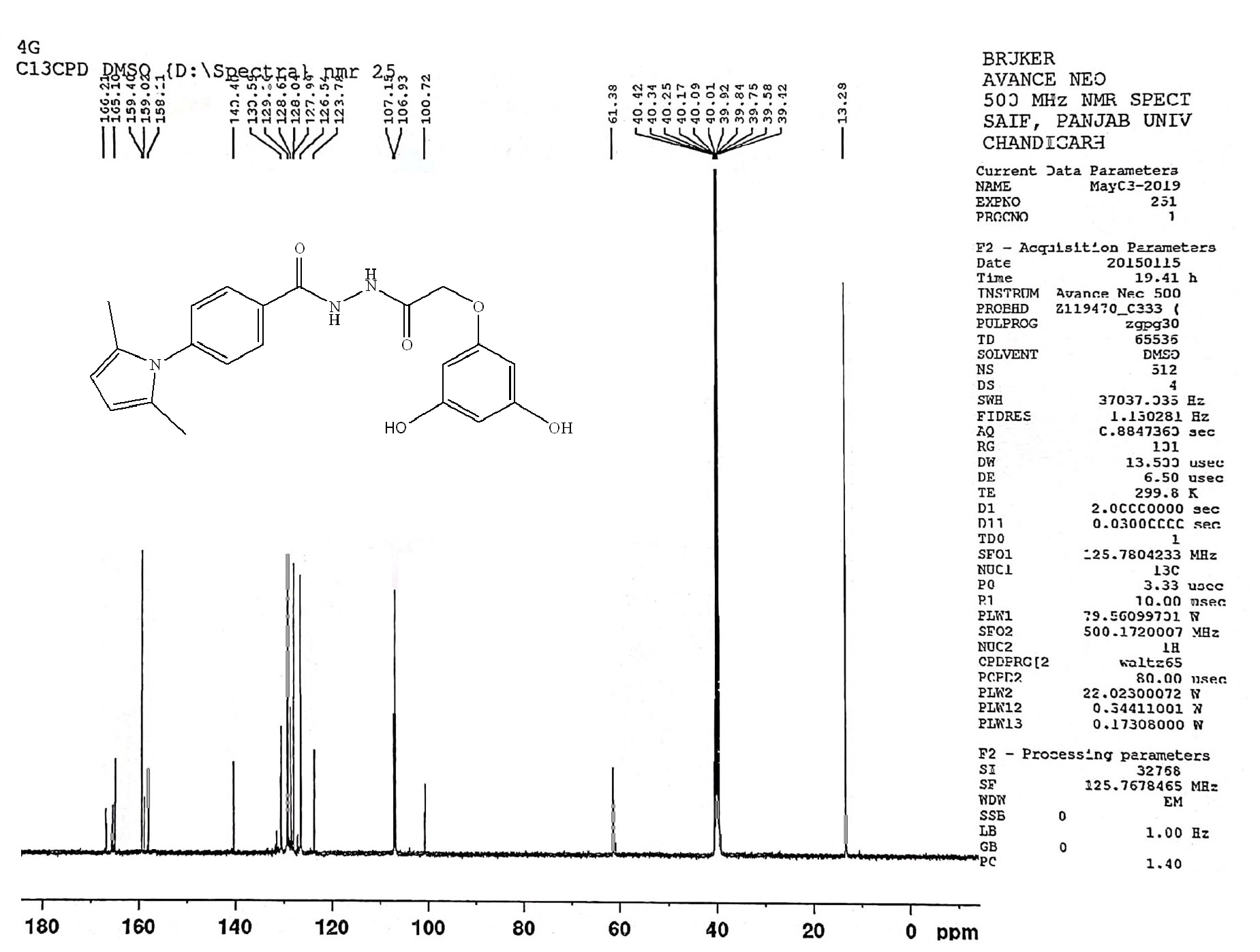


SPECTRUM 55: MASS SPECTRUM OF COMPOUND 5G


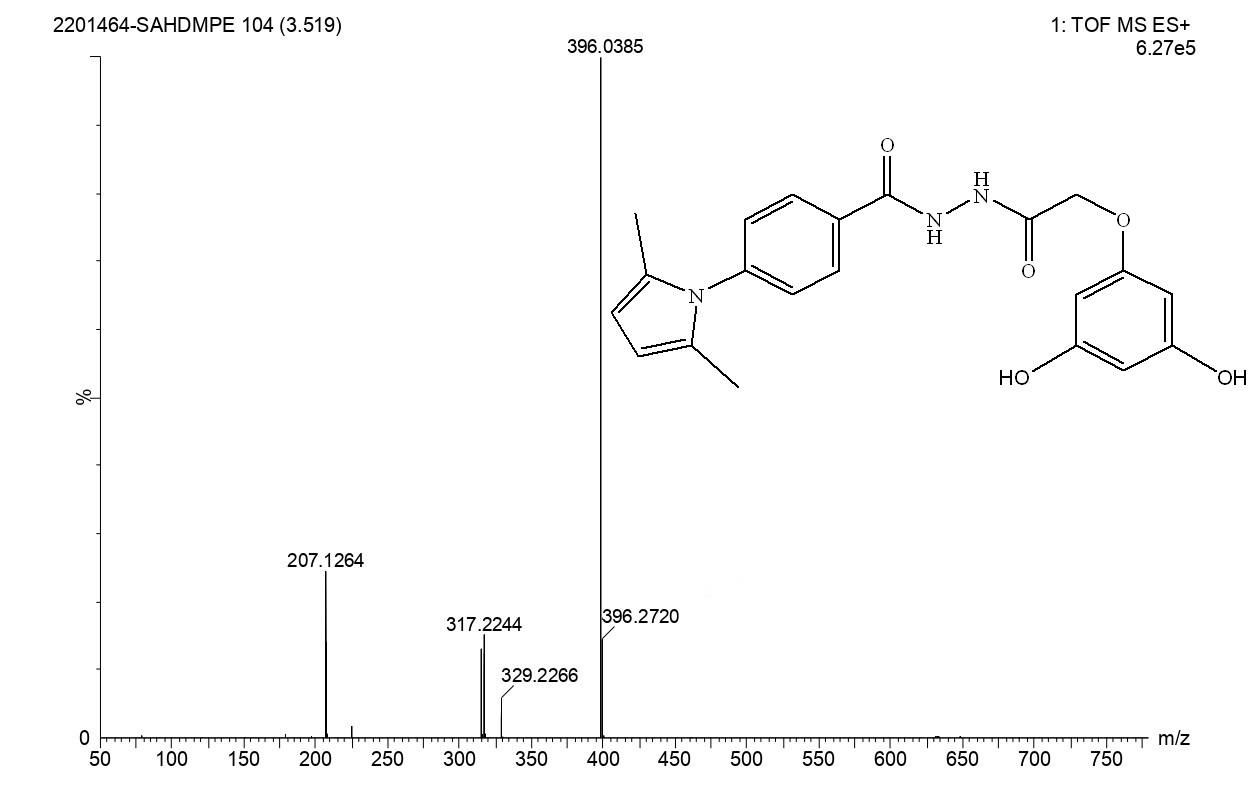


SPECTRUM 56: IR SPECTRUM OF COMPOUND 5H


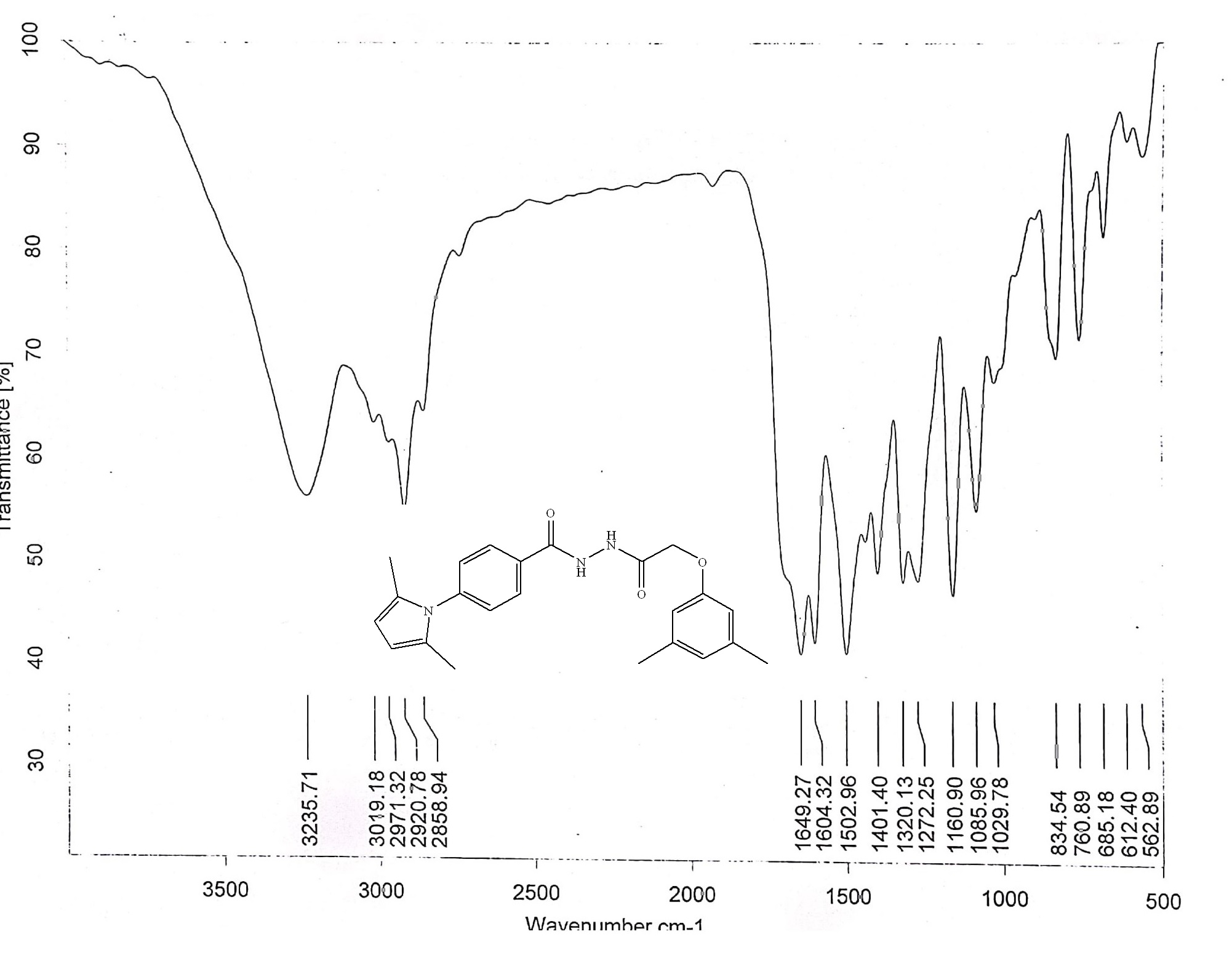


SPECTRUM 57: ^1^HNMR SPECTRUM OF COMPOUND 5H


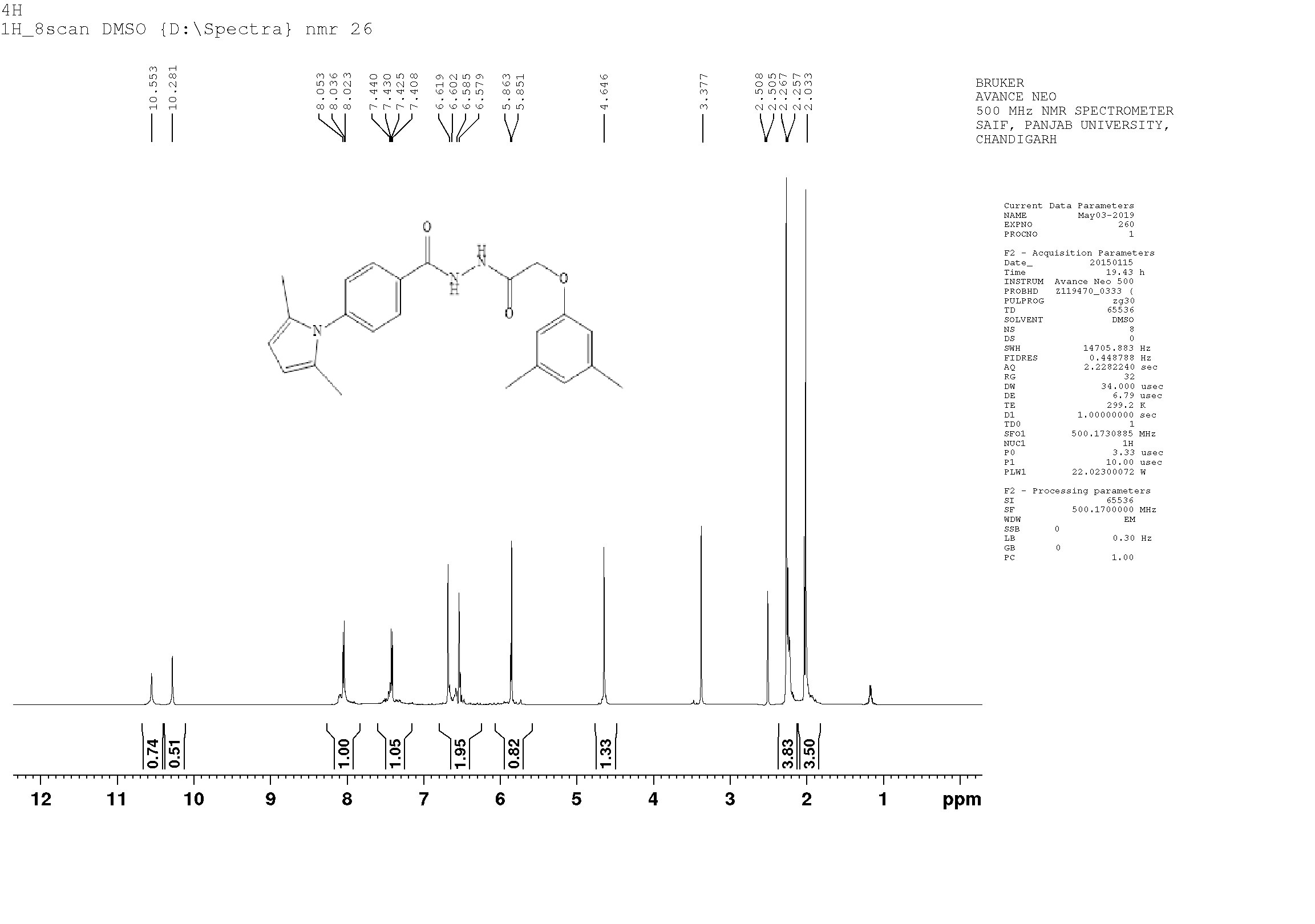


SPECTRUM 58: ^13^ CNMR SPECTRUM OF COMPOUND 5H


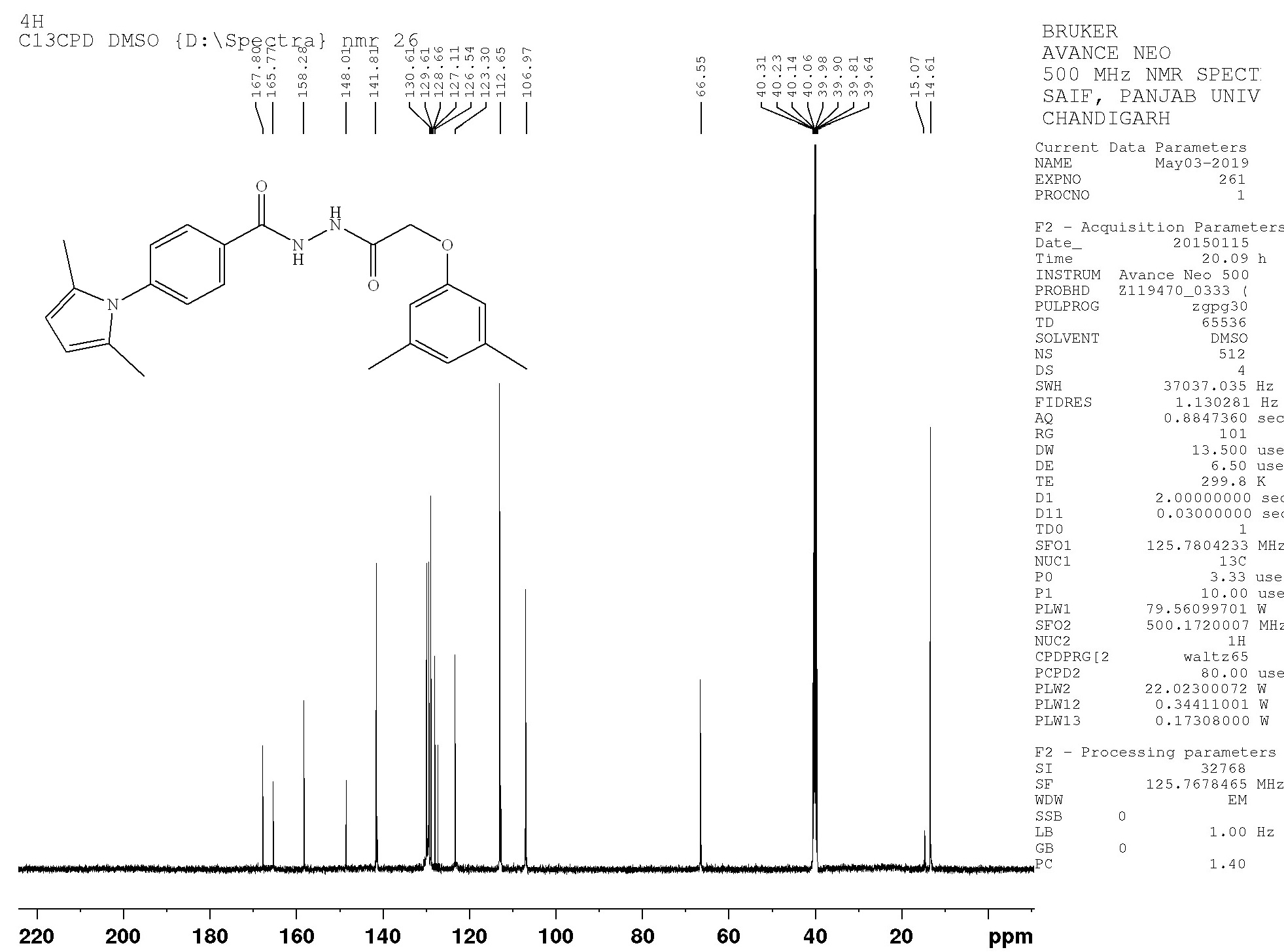


SPECTRUM 59: MASS SPECTRUM OF COMPOUND 5H


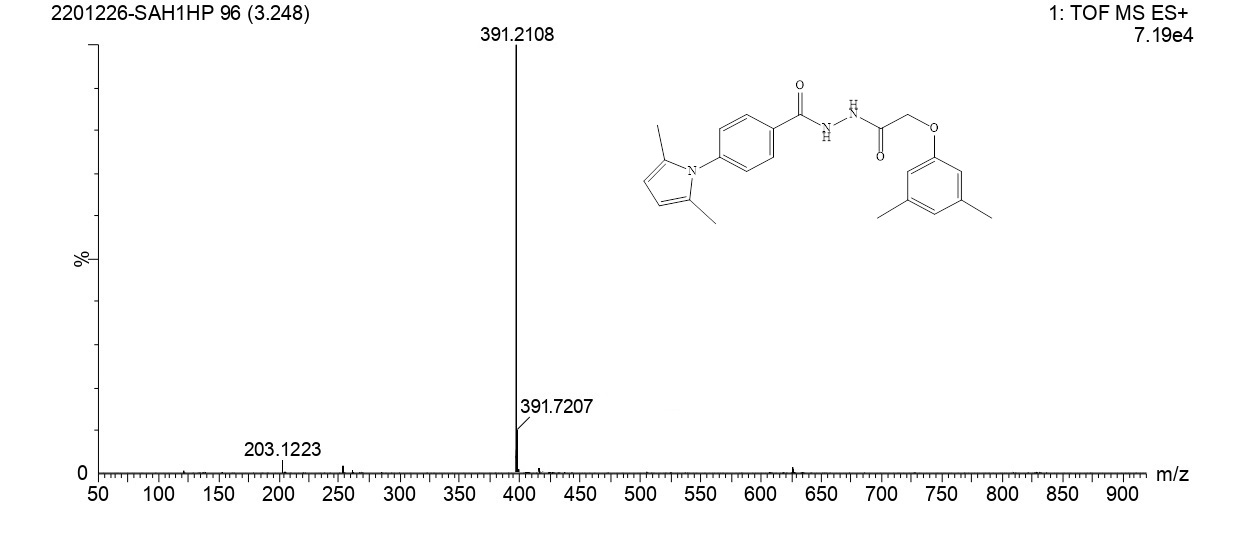


SPECTRUM 60: IR SPECTRUM OF COMPOUND 5I


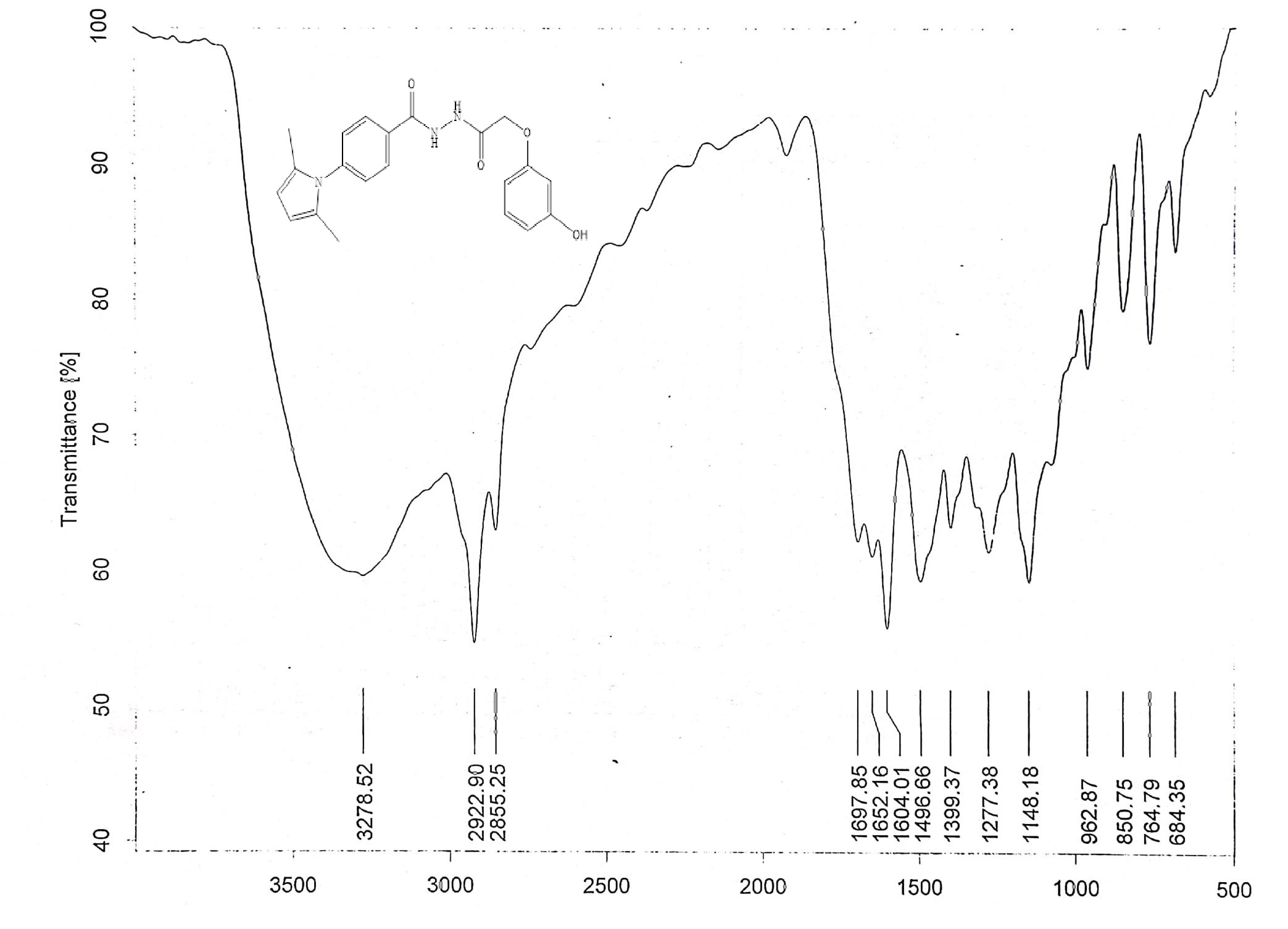


SPECTRUM 61: ^1^HNMR SPECTRUM OF COMPOUND 5I


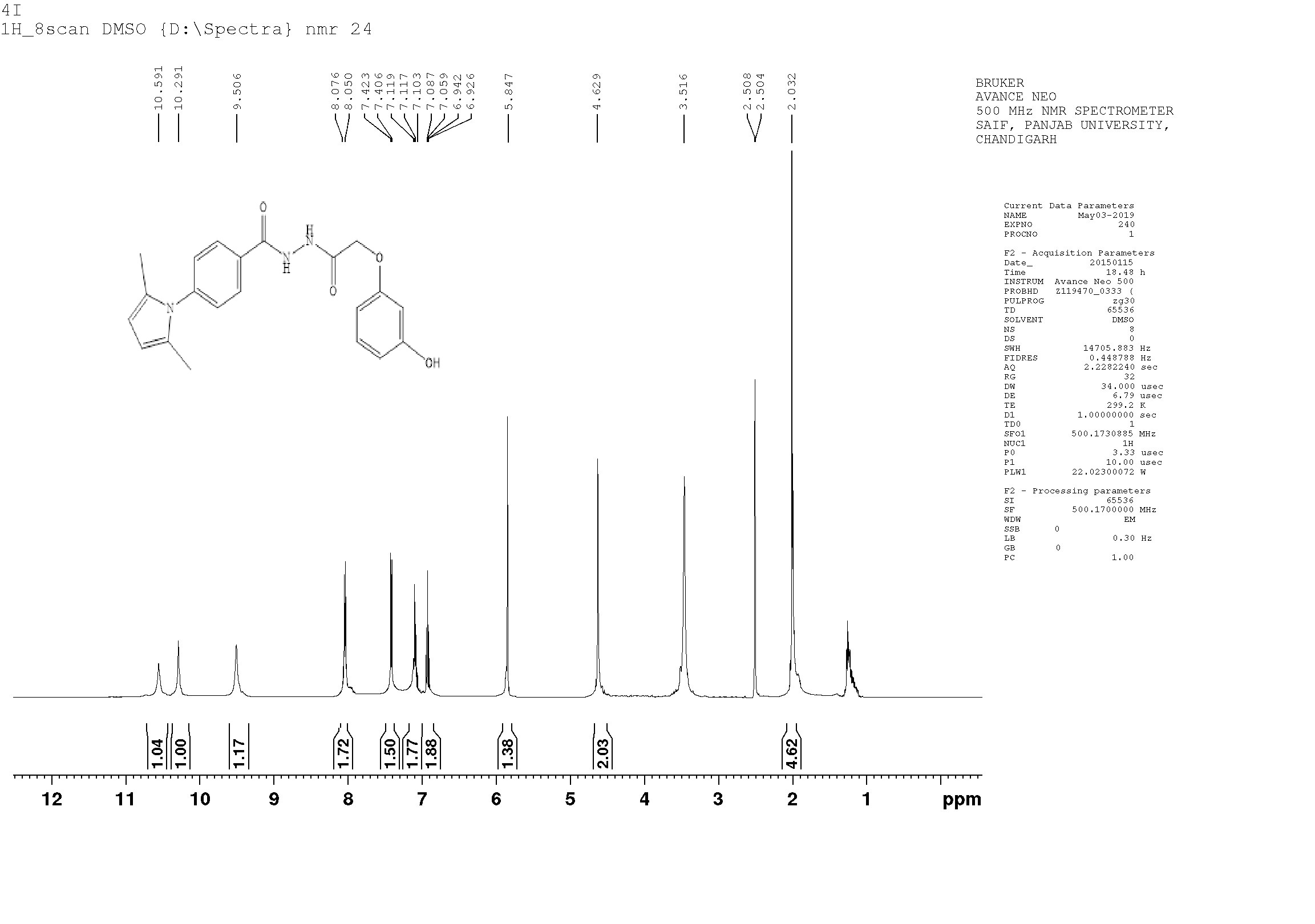


SPECTRUM 62: ^13^ CNMR SPECTRUM OF COMPOUND 5I


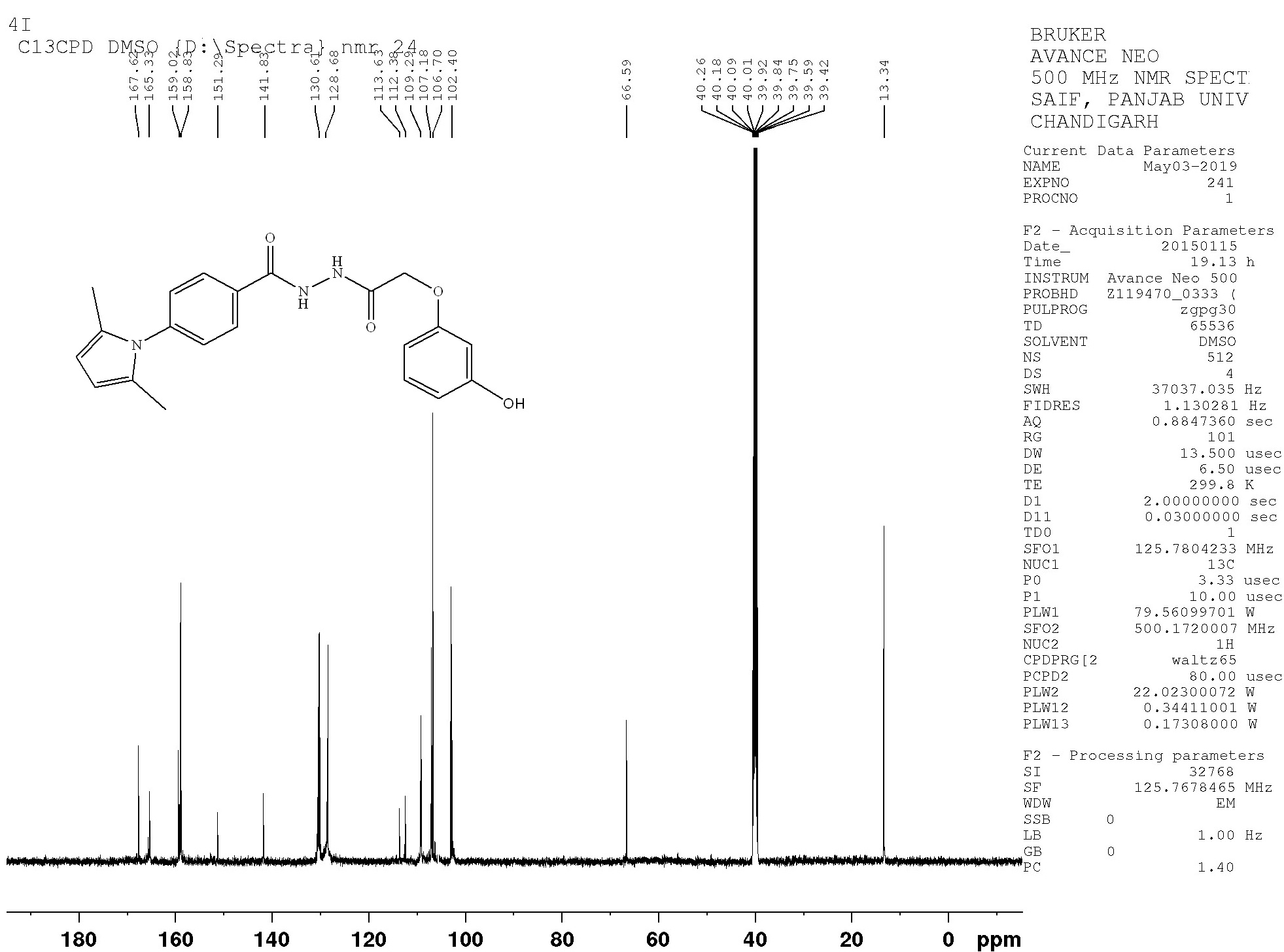


SPECTRUM 63: MASS SPECTRUM OF COMPOUND 5I


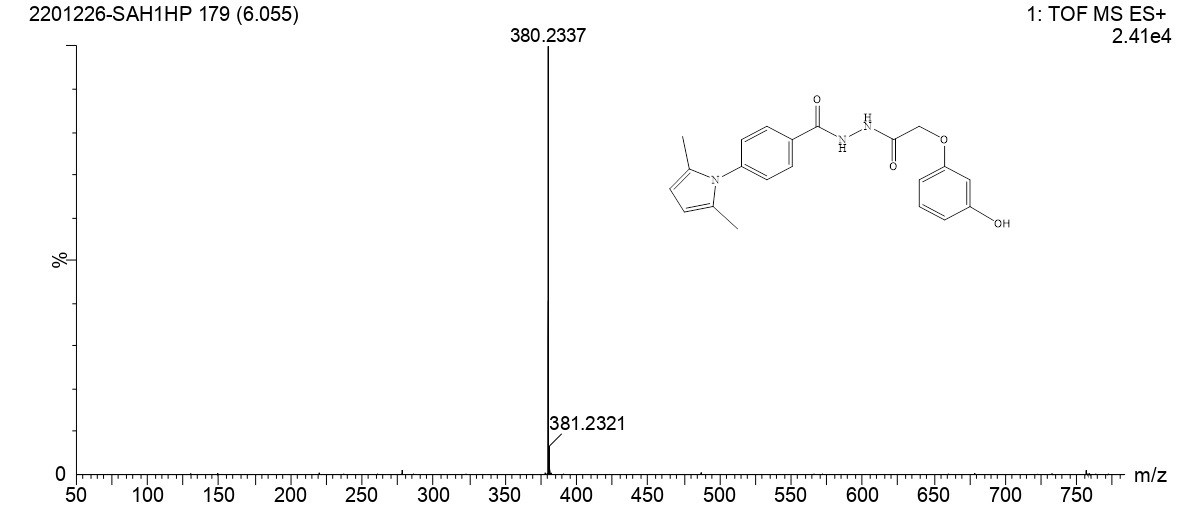


SPECTRUM 64: IR SPECTRUM OF COMPOUND 5J


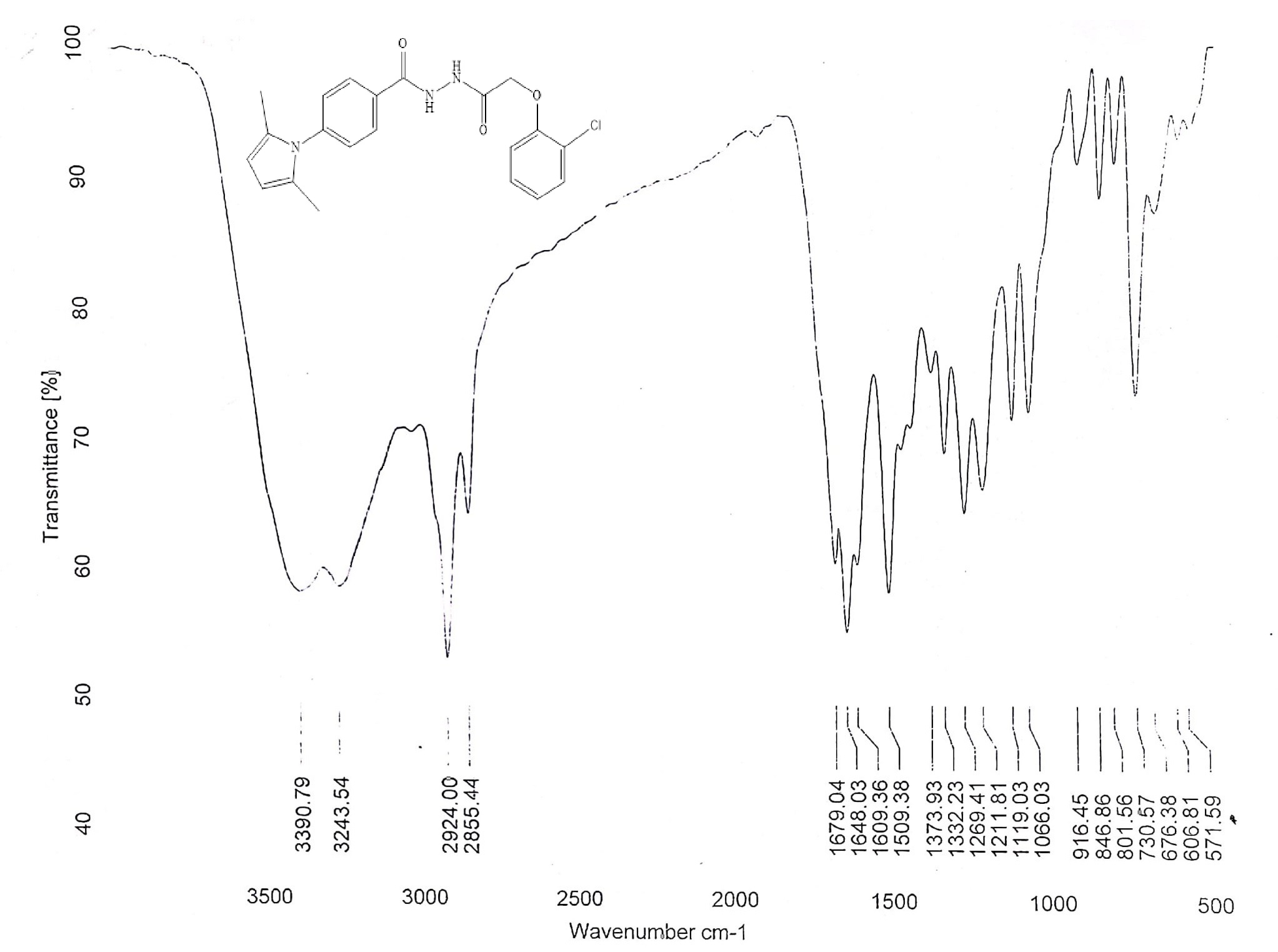


SPECTRUM 65: ^1^HNMR SPECTRUM OF COMPOUND 5J


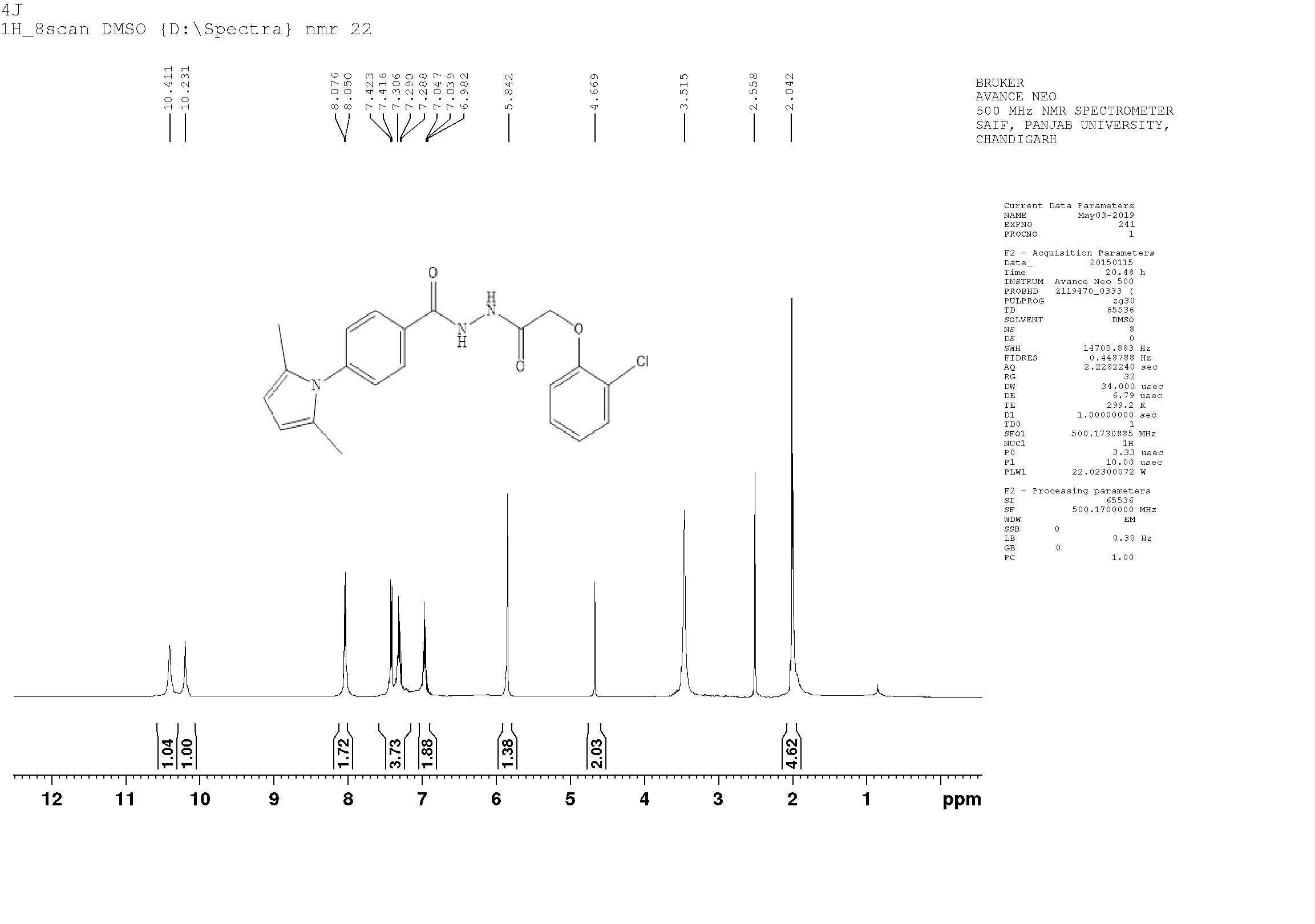


SPECTRUM 66: ^13^ CNMR SPECTRUM OF COMPOUND 5J


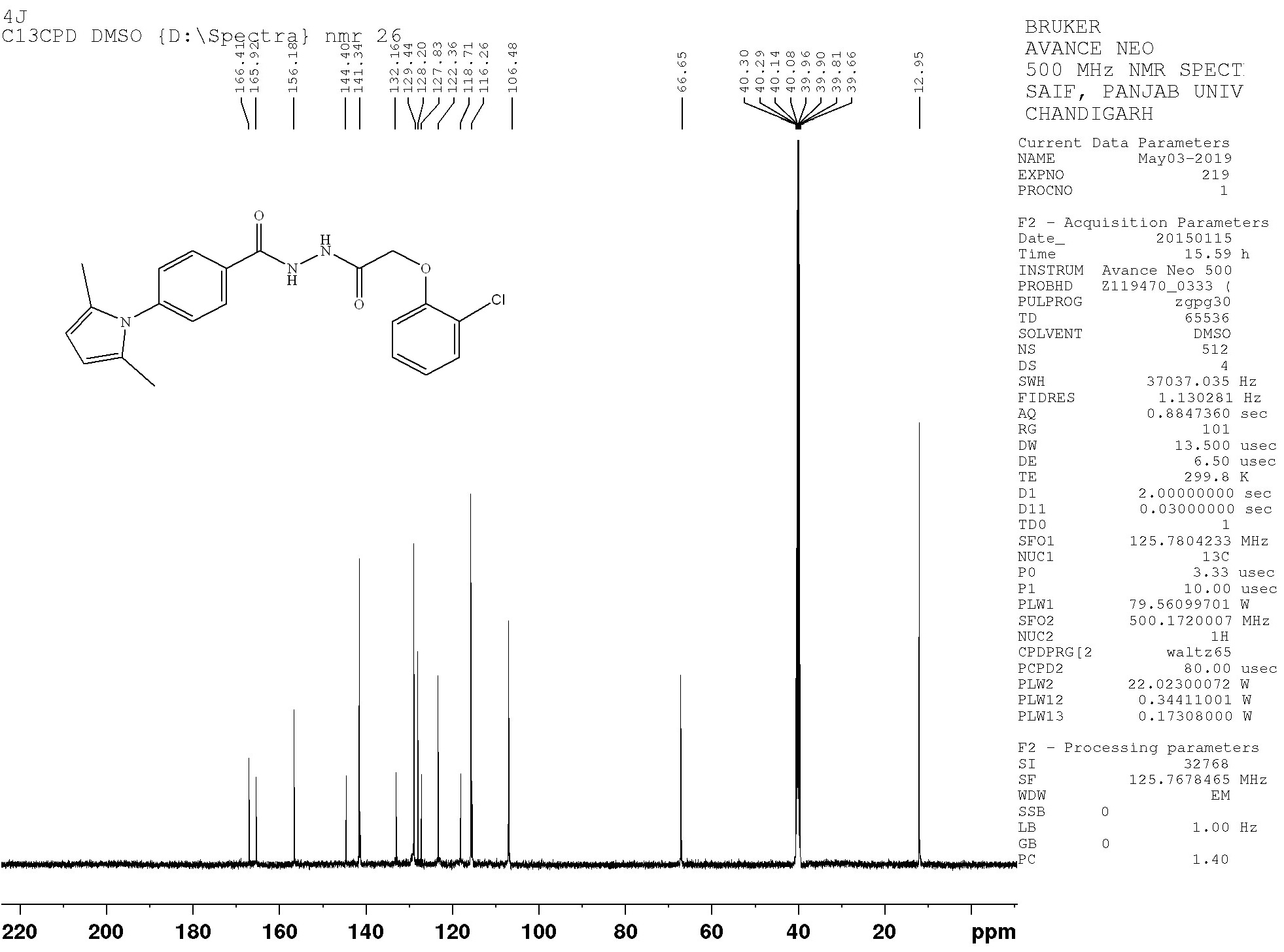


SPECTRUM 67: MASS SPECTRUM OF COMPOUND 5J


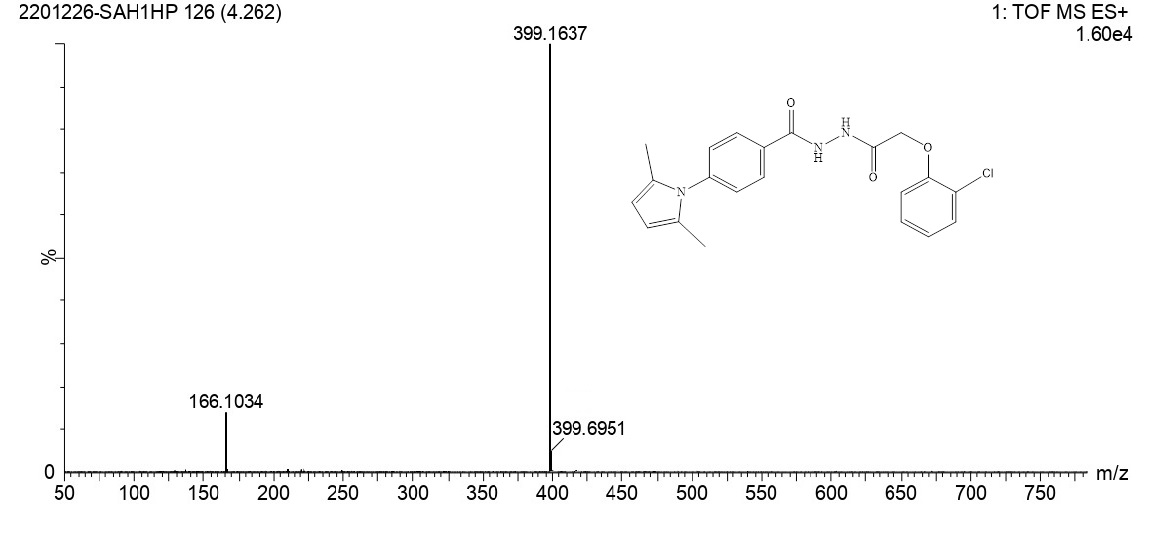

Supplement: S1 File — (DOC) [file pone.0303173.s002.doc]
